# Detection of the pathological exposure of pulp using an artificial intelligence tool: a multicentric study over periapical radiographs

**DOI:** 10.1186/s12903-023-03251-0

**Published:** 2023-08-11

**Authors:** A. Altukroni, A. Alsaeedi, C. Gonzalez-Losada, J. H. Lee, M. Alabudh, M. Mirah, S. El-Amri, O. Ezz El-Deen

**Affiliations:** 1 Ministry of Health, Medina, Saudi Arabia; 2 Department of Computer Science, College of Computer Science and Engineering, Taibah University, Medina, Saudi Arabia; 3 School of Dentistry, Complutense University of Madrid, Madrid, Spain; 4 Department of Periodontology, College of Dentistry and Institute of Oral Bioscience, Jeonbuk National University, Jeonju, Korea; 5 Department of Dental Materials, Taibah University, Medina, Saudi Arabia; 6 Ministry of Health, Ankara, Turkey; 7 Ministry of Health, Cairo, Egypt

**Keywords:** Artificial Intelligence, Caries, Deep Learning, Pulp Exposure, Periapical Radiograph, Yolov5x

## Abstract

**Background:**

Introducing artificial intelligence (AI) into the medical field proved beneficial in automating tasks and streamlining the practitioners’ lives. Hence, this study was conducted to design and evaluate an AI tool called Make Sure Caries Detector and Classifier (MSc) for detecting pathological exposure of pulp on digital periapical radiographs and to compare its performance with dentists.

**Methods:**

This study was a diagnostic, multi-centric study, with 3461 digital periapical radiographs from three countries and seven centers. MSc was built using Yolov5-x model, and it was used for exposed and unexposed pulp detection. The dataset was split into a train, validate, and test dataset; the ratio was 8–1-1 to prevent overfitting. 345 images with 752 labels were randomly allocated to test MSc. The performance metrics used to test MSc performance included mean average precision (mAP), precision, F1 score, recall, and area under receiver operating characteristic curve (AUC). The metrics used to compare the performance with that of 10 certified dentists were: right diagnosis exposed (RDE), right diagnosis not exposed (RDNE), false diagnosis exposed (FDE), false diagnosis not exposed (FDNE), missed diagnosis (MD), and over diagnosis (OD).

**Results:**

MSc achieved a performance of more than 90% in all metrics examined: an average precision of 0.928, recall of 0.918, F1-score of 0.922, and AUC of 0.956 (*P*<.05). The results showed a higher mean of 1.94 for all right (correct) diagnosis parameters in MSc group, while a higher mean of 0.64 for all wrong diagnosis parameters in the dentists group (*P*<.05).

**Conclusions:**

The designed MSc tool proved itself reliable in the detection and differentiating between exposed and unexposed pulp in the internally validated model. It also showed a better performance for the detection of exposed and unexposed pulp when compared to the 10 dentists’ consensus.

**Supplementary Information:**

The online version contains supplementary material available at 10.1186/s12903-023-03251-0.

## Background

Dental caries is a multifactorial disease caused by the demineralization of the tooth's hard tissues and the product of bacterial activity [1]. Consequently, a caries lesion develops, which may lead to pulpal exposure. It is necessary to avoid pulpal exposure since the vitality of the pulp is critical to the tooth's ability to function physiologically in the mouth. In most cases, pulpal inflammation is caused by microbial attacks. As the carious lesion reaches the pulp, the intensity of the inflammatory response rises [2]. The magnitude and severity of pulp-related issues should not be underestimated because they can result in oral sepsis. Consequently, proper diagnosis and management are critical. To determine an appropriate type of restoration and treatment planning, practitioners must have a precise understanding of the status of exposure [2]. 

Furthermore, clinical and radiographic tests are the major criteria for determining the depth and risk of dental caries to prevent pulpal exposure. In addition, the thickness of residual dentine cannot be assessed clinically, which is why a radiographic examination is required to support the clinician's decision [2]. 

However, utilizing radiographs alone to detect pulp exposure is difficult, especially for dentists who lack specialized expertise or time allocated to comprehensive examination. Numerous studies have reported that the reliability and accuracy of identifying dental caries vary greatly depending on the clinician's degree of expertise [3–6]. 

This is why it is critical to develop methods for determining the pulp status of teeth with deep caries. One of these methods is to employ Artificial Intelligence (AI) [7]. 

Today, AI is beginning to emerge in the healthcare field, reducing diagnostic mistakes in regular practice. Several studies have demonstrated that AI can match or surpass human experts in image-based diagnoses from a variety of medical specialties, including pneumonia in radiology (a convolutional neural network (CNN) trained with labeled frontal chest X-ray images outperformed radiologists in detecting pneumonia), dermatology (a CNN trained with clinical images was found to accurately identify skin lesions), and pathology (a CNN diagnosed heart attack with high accuracy) [8–11]. 

In addition, AI has piqued the interest of many dental researchers, particularly in dental radiography. Numerous well-written evaluations that presented fundamental principles or radiologist's guides of AI application have been published, attracting more dental researchers to explore its use in dentistry [12–15]. The current rapid advancement of technology has also spurred the development of numerous AI applications for dental radiography [16, 17]. 

Using AI, such as CNNs, may aid in detecting tiny anomalies in radiographic images that are otherwise difficult to identify with the naked eye. This proposes the possibility of using certain algorithms as automated tools to aid in the detection of deep caries and pulpitis [7]. 

AI can also help detect proximal caries and periapical pathologies that are frequently missed by human eyes on radiographs because of picture noise and/or low contrast [18]. Several researches revealed high-performance results in diagnosing dental caries in radiographies using various image processing approaches followed by machine leaning (ML) classifiers [18–23].

Dentist’s workflow became more efficient with automated suggestions for complex cases, better treatment planning, and prediction of diseases and outcomes [2]. 

Hence, this study was conducted to design and evaluate an AI tool called Make Sure Caries Detector and Classifier (MSc) for detecting pathological exposure of pulp on digital periapical radiographs and to compare how correct is the diagnosis between MSc and Dentists. The study was testing the hypothesis to evaluate if the designed AI tool was able to detect exposed/unexposed pulp caries correctly as compared to dentists. MSc was a trial model created by Smile with Confidence (SWC) Company to test the ability of AI to detect exposed and unexposed pulp.

## Methods

### Study design

This study was designed as a retrospective, diagnostic, and multi-centric study to develop and assess an AI tool for detecting pathological pulp exposure on digital periapical radiographs and comparing how accurate the diagnosis was between MSc and dentists. The reporting in this study follows the checklists for STARD 2015 [24] and Artificial Intelligence in Dental Research [25].

The study protocol was approved by the Institutional Review Board (IRB) in the local committee for ethics of health and scientific research in health affairs in Medina region (IRB 25/2021), Daejeon Dental Hospital, Wonkwang University College of Dentistry, and Complutense University of Madrid, Spain. Informed consent was waived by the IRB in the local committee for ethics of health and scientific research in health affairs in Medina region (IRB 25/2021) due to retrospective nature of the study. All methods were performed in accordance with the Declaration of Helsinki.

### Study population

Three thousand four hundred sixty-one anonymized labeled digital periapical radiographs with 3106 exposed pulp caries and 4612 unexposed pulp caries were selected between April 2021 and November 2021 from seven centers in three countries, including Saudi Arabia *(Specialized Dental Center, Aohd Dental Center, Alhijra Dental Center, and Faculty of Dentistry, Taibah University)*, Spain *(Faculty of Dentistry, Complutense University of Madrid)*, and Korea *(Faculty of Dentistry Daejeon Dental Hospital)*. The periapical radiographs included in this research were retrospectively selected by two certified dentists from 18,000 collected periapical radiographs.

The research included all sizes (size 0, 1, and 2 and as JPG and TXT format) of digital periapical radiographs. All carious teeth, including those with periapical or periodontal issues were included. Additionally, radiographs with noticeable caries by human eyes were included (after interrater agreement between two collaborated dentists), whether permanent or deciduous, anterior or posterior teeth, and upper or lower. Radiographs with any number of exposures or carious lesions were included.

Digital periapical radiographs with root caries, restorations (intra-coronal, crowns, and bridges), orthodontic brackets, and wires affecting the interpretation of the carious teeth were excluded. Also, radiographs with more than half of the film missing or unclear or that are difficult to discern due to extreme distortion, artificial noise, blur, and poor image quality were omitted.

### Data cleaning and labeling

The data from each hospital or dental center was cleaned and labeled internally by two collaborated, qualified endodontists with more than two years of experience before being sent to the principal investigator (PI) (Specialist of Restorative Dentistry) through an electronic cloud (Google Drive). All radiographs from restorative, endodontic, and pedodontic categories were collected. The PI then revised the cleaning and labeling processes by randomly distributing all collected labeled data to another two dental practitioners with more than two years of experience, who checked and confirmed that all radiographs included dental caries and met our eligibility criteria and then excluded any data that did not. All data was labeled and confirmed when the diagnosis was confirmed by the two dentists; any radiograph on which the interrater disagreed was excluded. At last, the qualified information was sent to the electronic cloud (Google Drive) of the PI.

All confirmed radiograph images were sent to 10 clinicians (ages 25–32, endodontists and general practitioners, two females and eight males, and from the organizations from which data was collected), who manually classified the digital periapical radiographs based on whether the pulp was exposed or not using "Labellmg (Windows_v1.8.0, tzutalin)". This was used to establish the gold standard. A different digital file was sent to each clinician. The annotation was made using boxes.

The PI received the clinicians’ responses as radiographs images in JPG format and TXT files as YOLO format, without knowing their names or contacts (only their titles were known). Clinicians in various hospitals were not given access to each other's data, so they were unfamiliar with one another. All radiographs were coded by sequential numbers. Later, during data processing, the labeled dataset was randomly divided into train (2755), validate (345) (internal validation), and test (345) datasets using Python's random package. The two programmers had full access to all labeled data.

QuestionPro testing platform was used for randomization. All data was randomized by sample randomization method and was masked.

### Data processing

**Fig. 1:**
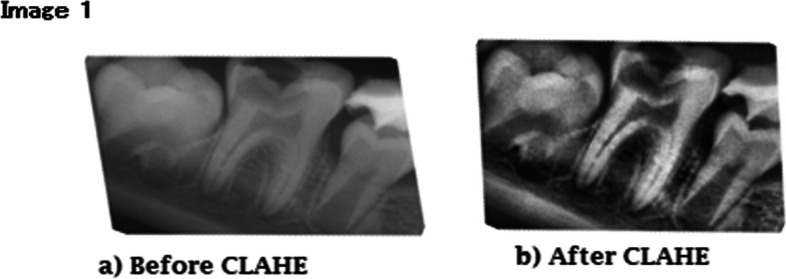
Before and After CLAHE

Pre-processing procedures were applied using CLAHE to create more contrasted black-and-white images with clipLimit = 5.0 (Fig. 1). The dataset was divided using an 8–1-1 ratio. The test dataset was used to test the MSc and compare its results to those of the other 10 dentists. The validation set, on the other hand, was solely used for validation.

**Fig. 2:**
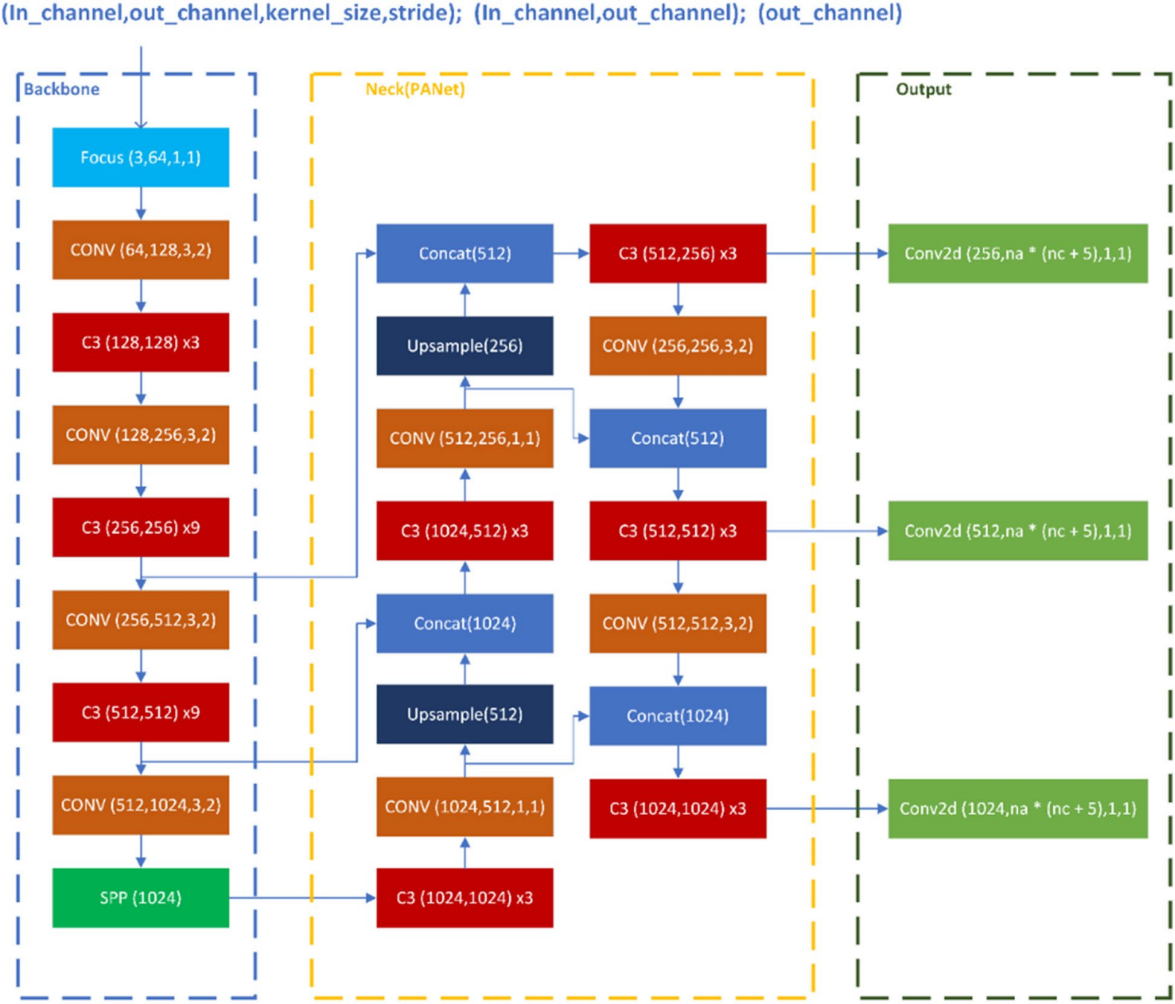
Yolov5 network architecture

The MSc model makes use of a variety of CNN algorithm optimization techniques, including auto-learning bounding box anchors, mosaic data augmentation, and the cross-stage partial network. Yolo (You Only Look Once) is an object detection method that it employs. It divided images into cells. Each cell is in charge of detecting objects within it. Yolo processes the entire image with a single neural network, then divides it into sections and predicts the bounding boxes for each section. This algorithm looks at the image once, making predictions after a single forward pass through the neural network. The identified objects are then delivered. Its architecture is primarily composed of three components (Fig. 2):

- Backbone (CSPDarknet): It is used to extract key features (rich in useful characteristics) from the input image
- Neck (PANet): A series of layers to mix and combine image features to pass them forward to prediction
- Head (Output): It is responsible for the final detection step

The data is initially sent into the backbone for feature extraction, then into PANet for feature fusion, and lastly into the Yolo layer for output.

The following parameters and hyperparameters were used in our model:

- Image size: 640
- Initial weights: yolov5s.pt
- epoch: 50
- Learning Rate: 0.001
- Momentum: 0.999
- Optimizer: SGD
- Batch Size: 8
- IoU threshold: 0.5
- Device: Running on GPU with Cuda from AWS (p3.2xlarge)

### Study groups

The randomized test dataset included 345 images with 752 labels and was used to assess MSc's performance. The data was also utilized to compare the diagnostic disparities between 10 qualified dentists with more than two years of experience and the MSc tool. The data for the dentist groups was randomly divided into 10 blocks, with each block including 34 to 35 radiographs. Data was labeled at various intervals since it was gathered at different times. The reference standards were provided to the performers when using MSc.

## Reference test

### MSc metrics

#### Primary

Mean Average Precision 0.5 (mAP@0.5): mAP, calculated by taking the mean AP (accuracy of our AI tool) over all exposed and unexposed pulp caries and/or overall 0.5 (IoU) thresholds

#### Secondary

- Precision (Specificity): The ratio of correctly predicted positive exposed/unexposed pulp caries to the total predicted exposed/unexposed pulp caries: Prec. = TP/ TP + FP
- Recall (Sensitivity): Calculates how many actual exposed/unexposed pulp caries true positives the model has captured, labeling them as positives. Recall = TP/TP + FN
- F1 Score: Defined as the function of precision and recall. It is calculated when a balance between precision and recall is needed. F1 = 2 × Precision × Recall/ Precision + Recall
- AUC: Area under the receiver operating characteristic (ROC) curve (AUC). AUC integrated from (0, 0) to (1, 1) gave the aggregate measure of all possible exposed and unexposed pulp caries detection and classification thresholds

### Diagnostics metrics

#### Primary

- Correct Diagnosis: Overall diagnostic accuracy (true negatives + true positives)

#### Secondary

- Wrong diagnosis: The sum of False Diagnosis Exposed (FDE), which is all exposed pulp caries that were diagnosed as unexposed pulp caries based on the gold standard, False Diagnosis Not Exposed (FDNE), which is all unexposed pulp caries that were diagnosed as exposed pulp caries based on the gold standard, Over Diagnosis (OD, Exposed\Unexposed), which is all non-carious objects that were diagnosed as exposed pulp caries or unexposed pulp caries based on the gold standard, and Missed Diagnosis (MD, Exposed\Unexposed), which is all exposed\unexposed pulp caries that were undiagnosed based on the gold standard.

### Sample size calculation

According to the results of Lee et al., study [3],  which aimed to estimate optimal deep CNN algorithm weight factors for training and validation dataset of both carious and non-carious molars and premolars teeth, at diagnostic accuracy 82.0%, sensitivity 81.0%, specificity 83.0%, PPV 82.7%, and NPV 81.4%, and with an alpha error of 5% and a confidence interval of 95%, a sample size of 3,000 periapical radiographs in total were chosen. To achieve higher diagnostic performance metrics, the teeth were not classified based on tooth position, and 3,445 digital periapical radiographs were selected in this study.

### Statistical analysis

The descriptive metrics of MSc model performance presented as the percentage of mAP @0.5, Precision, Recall, F1 score, and AUC of the test dataset were calculated using the Keras library on top of TensorFlow "Yolo v5" in Python. On the other hand, the mean, frequency distribution, median, and range of the diagnostics measures (RDE, FDE, RDNE, FDNE, OD, MD, correct diagnosis, and wrong diagnosis) were calculated to compare both the performance of the MSc model and 10 certified dentists.

The Wilcoxon Signed Ranks Test was used to determine the significant difference between the values (RDE, FDE, RDNE, FDNE, OV, MD, correct diagnosis, and wrong diagnosis) for both the dentists over test dataset and MSc over the test dataset. The statistical significance was set at *p* < 0.05.

## Results

**Fig. 3:**
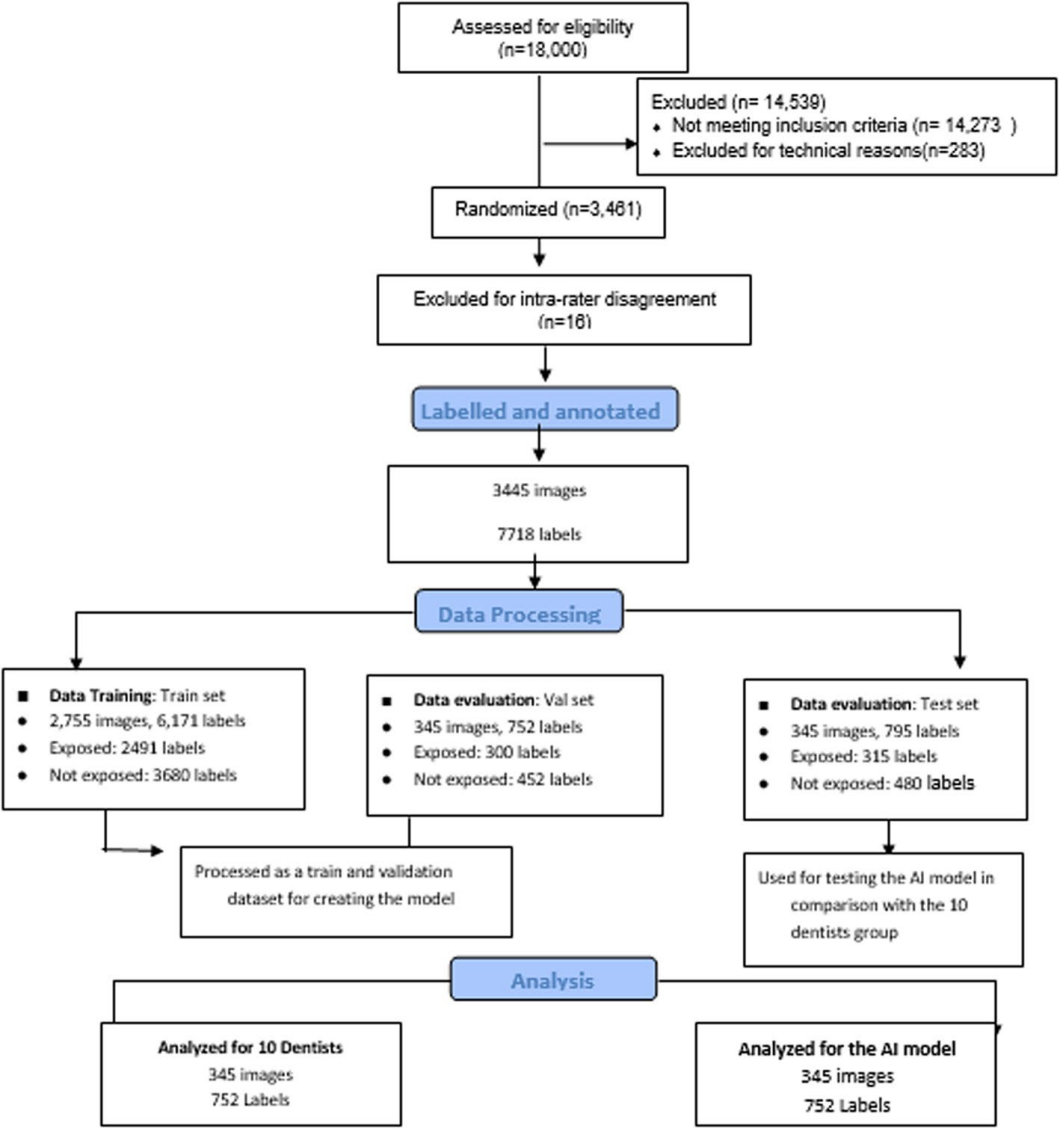
Flow Diagram

The total data assessed for eligibility consisted of 18,000 images; however, 14,539 images were excluded for not meeting the inclusion criteria and for technical reasons. Most images were excluded because they were caries free; therefore, only 3461 images were randomized. In addition, further 16 images were excluded because of inter-rater disagreement; hence, 3445 images were labeled and annotated (7718 labels in total). The data were split in a ratio of (8:1:1) for train 2491 (exposed), test (315 exposed), and validate (300 exposed), respectively. The train set consisted of 2,755 images (6,171 labels), the validation set consisted of 345 images (795 labels), and the test set comprised of 345 images (752 labels). Lastly, the test dataset was used for analyzing MSc group and dentists group. (Tables 1, 2) (Fig. 3).

**Table 1:** Data and labelled distribution

Observed Frequency	Train Set	Val Set	Test Set	Total
Images	2755	345	345	3445
Exposed Level	2491	315	300	3106
Not-Exposed Level	3680	480	452	4612

**Table 2:** Data Evaluation

IoU = 0.5	TP(labels	FP(labels)	FN(labels)	Precision	Recall	F1-score	mAP@.5(AUC)	mAP@.5.95
All	691	56	61	0.928	0.918	0.922	0.956	0.613
Exposed	277	20	23	0.93	0.92	0.925	0.97	0.665
Not exposed	41,435	35	38	0.926	0.916	0.921	0.943	0.56

### MSc performance metrics

**Fig. 4:**
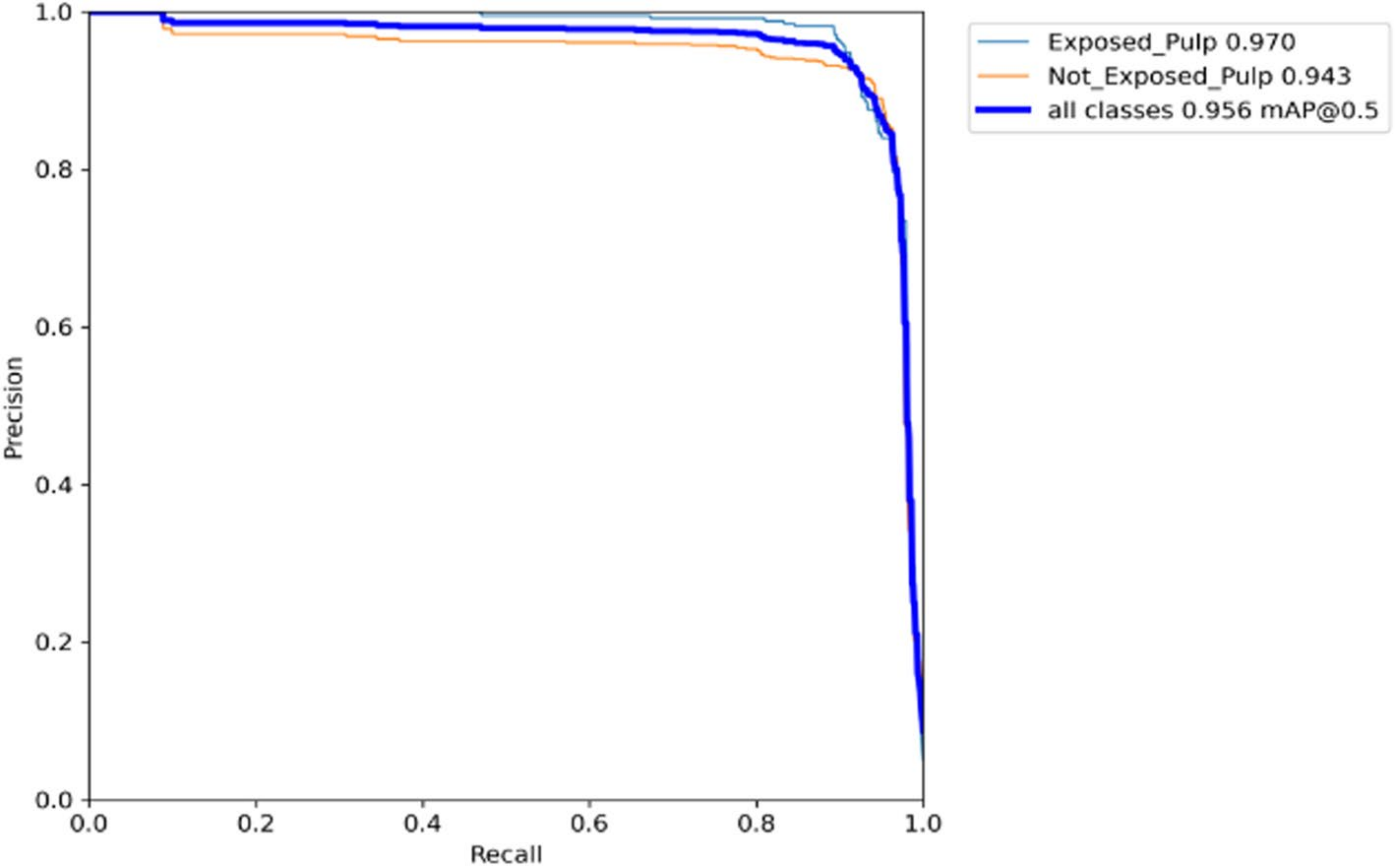
AUC for Precision-Recall curve

**Fig. 5:**
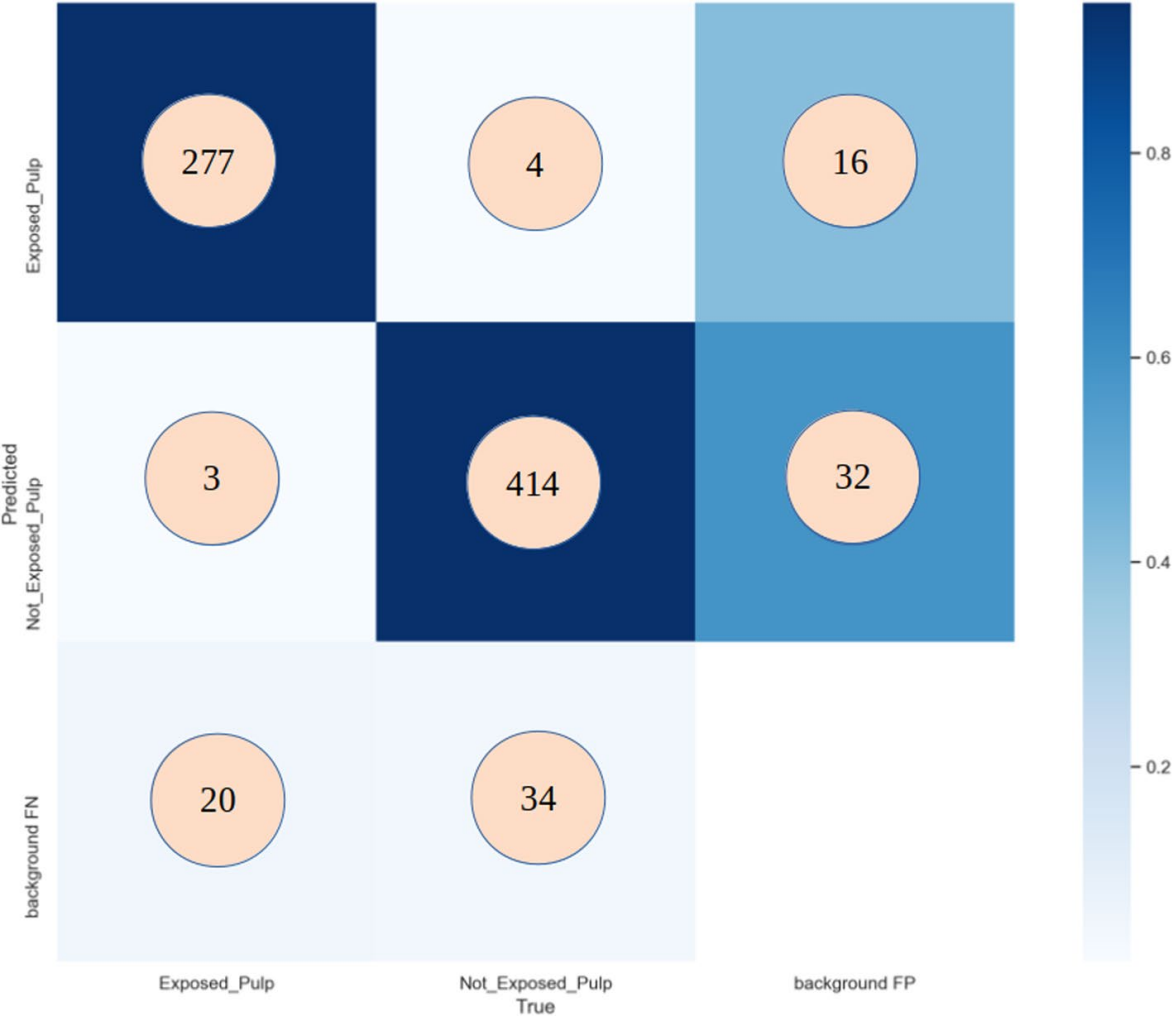
Confusion Matrix for the test dataset

The number of true positive (TP) exposed and unexposed pulp caries that were detected by MSc over the test dataset is 691 labels. The number of false positive (FP) exposed and unexposed pulp caries that were detected by MSc over the test dataset is 56 labels, and the number of false negative (FN) exposed and unexposed pulp caries that were detected by MSc over the test dataset is 61 labels. Therefore, the obtained results of our model showed an mAP < 0.5 of 95.6%, precision of 92.8%, a recall of 91.8%, and an F1 score of 92.2% (*P* > 0.05) (Table 3). The AUC value was 0.956. (*P* > 0.05) (Figs. 4 and 5).

**Table 3:** MSc performance metrics

	Precision	Recall	F1 Score	mAP(< 0.5)
All	0.928	0.918	0.922	0.956
Exposed	0.93	0.925	0.925	0.97
Not Exposed	0.926	0.921	0.921	0.943

^*^All results were statistcaly signifcant *P* < 0.05

### Diagnostics measures

The mean value of RDE, RDNE, OD, and RD for the MSc group was higher than that of the dentists group. Meanwhile, the mean value for the FDE, FDNE, MD, and wrong diagnosis for the MSc group was lower than that of the dentists group. The mean and median values are listed in (Table 4).

**Table 4:** Dentists versus Msc’s diagnosis

	Dentists Over Test Data				MSc Over Test Data				*P*
	Mean	Min	Median (Q1,Q3)	Max	Mean	Min	Median (Q1,Q3)	Max	
RD Exposed	0.64	0	1(0,1)	4	0.81	0	1(0,1)	4	.000
FD Exposed	0.05	0	0(0,0)	2	0.01	0	0(0,0)	1	.001
RD Not Exposed	0.72	0	0(0,1)	6	1.14	0	1(0,2)	6	.000
FD Not Exposed	0.04	0	0(0,0)	2	0.01	0	0(0,0)	1	.008
Over Diagnosis	0.03	0	0(0,0)	3	0.08	0	0(0,0)	2	.028
Missed Diagnosis	0.51	0	0(0,1)	6	0.21	0	0(0,0)	3	.000
Right Diagnosis	1.36	0	1(1,2)	6	1.94	0	2(1,3)	6	.000
Wrong Diagnosis	0.64	0	0(0,1)	6	0.30	0	0(0,0.5)	3	.000

## Discussion

The current study designed and analyzed an AI tool named MSc to detect pathological pulp exposure on digital periapical radiographs, indicating that MSc diagnoses exposed/unexposed pulp caries more accurately than dentists. This is considered the first study to test the use of AI in detecting exposed and unexposed pulps. It's important to note that the tool isn't commercially accessible; it's an experimental model designed to evaluate AI's ability to recognize exposed and unexposed pulp.

It is challenging to diagnose dental caries using radiographs alone, especially for the dentists who do not have enough specialized training or time devoted for detailed diagnosis; the reasons are the settings of various parameters, including brightness, shadow, and contrast [3].

Nowadays, AI is becoming essential in radiology due to its ability to detect abnormalities in radiographic images unnoticed by the naked human eye [26]. However, many people, including clinicians and scientists, are not yet familiar with the concepts and true potential of AI and the impact that it can have on our personal and professional lives [27]. 

It can help in standardizing dental caries according to caries depth [7]. Caries detection in dental radiographs might be achieved by evaluating the radiodensity of images. The enamel and dentin of normal teeth are radiopaque. Caries cause these structures to lose their mineralization and hence become radiolucent [18]. Using these characteristics, AI prediction capability enables AI tools to "learn" to analyze dental radiographs and predict pulp status.

This is why we developed our MSc tool: to compare its accuracy in detecting exposed and unexposed pulps to that of dentists. Yolov5x, an object detection method, was used in this tool. We used 10 examiners from various hospitals and locations of the world in our investigation. The dentists who participated in the comparison were all qualified specialists, and their results served as the "gold standard." The inclusion of dentists from various hospitals and places of the world assured that diverse experiences in defining the gold standard would result in a broader picture of the many MSc outcomes [28].

However, this was not in line with a systematic review [29] that showed that trials with only one examiner yielded the best results, indicating that the same caries detection criteria were always utilized [18]. Also, another study used four experts to analyze the photos, which was considered the second best [3].  Finally, the trial with two examiners produced the worst accuracy result [30, 31].

The number of images (3445) used in our study was also comparable with that of Cantu et al., who used 3292 bitewing radiographs to detect carious exposure. When these data were compared to the technique and findings of the current investigation, the number of images employed and the verified diagnostic performance were nearly similar [32].

In the present study, MSc showed a high mAP (0.956) at 50% Confidence level in detecting exposed and unexposed pulp, and this is considered outstanding. In addition, with an AUC of 0.956 for all classes in our trial, MSc’s performance is considered exceptional since an AUC value of 0.8 to 0.9 is excellent, and more is exceptional [33].

MSc also showed statistically significant outcomes in detecting exposed and unexposed pulp caries accurately and with less wrong detections in comparison with the dentists group. This means that the model yielded better scores than the dentists. Moreover, the model seemed to be more effective and reliable than the dentists, since the 10 experienced dentists did not show good consistency and stability, and the model was much faster and more accurate in lesion classification. However, further research needs to be conducted with a larger dataset and different experienced dentists for more reliable results.

This was in line with a study [3] that used deep CNN-based computer-vision for dental caries detection, and the results were 89.0%, 88.0%, and 82.0% for diagnostic accuracies of premolar, molar, and both premolar and molar models, respectively. The deep CNN algorithm achieved an AUC of 0.917 on premolar, an AUC of 0.890 on molar, and an AUC of 0.845 on both premolar and molar models. In another study conducted by Kühnisch et al., the CNN accurately detected cavities in 92.5% of instances (SE, 89.6; SP, 94.3; AUC, 0.964). This was also similar to the finding in other studies [28, 32].

Another study in India evaluated the diagnostic performance of an algorithm where a neural network is developed to detect dental caries in digital radiographs. The system gave an accuracy of 97.1%, FP rate of 2.8%, ROC area of 0.987 and precision recall curve (PRC) area of 0.987. This is considered high accuracy compared to our study, but the authors only employed 105 photos and performed tenfold cross-validation; their dataset wasn’t enough [18]. 

Other accuracy comparisons with studies that used different AI methods were as follows: Singh et al. suggested a caries detection method based on Radon Transformation and DCT employing dental X-ray images [23].  Selected characteristics are retrieved using the PCA approach and used to the Random Forest classifier, yielding an accuracy of 86%.

During another study, [21] a caries detection system utilized SVM and got 86.15% accuracy for the training dataset and 77.34%accuracy for the test dataset.

A study [34] developed CNN-based categorization of major dental illnesses, which achieved an accuracy of 87.5% in detecting dental caries.

Additionally, a similar study [35] assessed the detection of carious exposure using AI on bitewings, and the neural network had an accuracy of 80%, while dentists had a considerably lower mean accuracy of 71%. The neural network demonstrated significant sensitivities of 70% or higher for both initial and advanced lesions. Dentists' sensitivities for initial lesions were generally low while those for advanced lesions ranged between 40 and 75%. Authors suggested that this was due to the dentists being prudent in their decisions, unlike the AI tool. When these data were compared to the technique and findings of the current investigation with MSc, the number of images employed and the verified diagnostic performance were nearly comparable. Nevertheless, our MSc model had higher mean in the over diagnosis variable, which means higher probability for detecting non carious objects as exposed or unexposed pulp caries. That can be viewed as limitation for AI, as it can consider some radiographic artificial changes as carious lesions. This is why it is important to note that the final diagnosis needs to be confirmed by the clinician as our tool is only a decision support system.

We also had an overfitting problem during our study. Overfitting is a crucial aspect of AI. "The essence of overfitting," according to Burnham and Anderson, "is to have unwittingly retrieved part of the residual variation as if that variation reflected underlying model structure" [36]. When a model is overfitted, it is so particular to the original data that applying it to data obtained in the future might result in problematic or incorrect outcomes, and so less-than-optimal judgments [37]. However, we tried to resolve this by increasing the number of training dataset.

There were also more limitations in our study. Clinical parameters were not included, which is an aspect that should be taken into account to have a more accurate diagnosis. Also, neural networks, in general, including our tool MSc, are black boxes that cannot explain machine learning characteristics and the grounds for making decisions based on that learning. The limitations of the digital periapical radiographs, such as image magnification and distortion and the lack of three-dimensional information, may lower the MSc tool's diagnostic accuracy. This is because it is challenging to diagnose critical cases, such as nearly exposed pulp with a small layer of dentine. Therefore, it is necessary to take clinical assessment into consideration. Also, the dataset was not divergent regarding the age and sex because it was collected without prior knowledge of the patients’ details. We also did not categorize the teeth according to their types, which may have affected the accuracy of the results according to the teeth type. Finally, further research needs to be conducted with a larger dataset and different experienced dentists for more reliable results.

Future initiatives for improving AI-based caries diagnosis on intraoral pictures should involve image segmentation as an alternate option, which should be carried out by well-trained and calibrated dental practitioners under the supervision of senior specialists. To accomplish this, caries lesions must be marked pixel by pixel on each accessible image and the diagnosis accuracy must be reassessed. In comparison to the currently utilized classification methodology, this more precise but otherwise time- and resource-intensive approach provides thorough caries localization.

## Conclusions

From the above results and discussion, it is concluded that:

1. The designed AI model proved itself reliable in the detection of pathological pulp exposure, and in the differentiation between exposed and unexposed pulp caries on digital periapical radiographs.
2. The designed AI model detected pathological pulp exposure on digital periapical radiographs more correctly and effectively than the10 dentists.

### Supplementary Information

**Additional file 1: **Test Dataset 1: Digital radiograph of upper posterior teeth. Test Dataset 2: Digital radiograph of upper posterior teeth, Test Dataset 3: Digital radiograph of upper posterior teeth, Test Dataset 4: Digital radiograph of upper posterior teeth, Test Dataset 5: Digital radiograph of upper anterior teeth, Test Dataset 6: Digital radiograph of upper anterior teeth, Test Dataset 7: Digital radiograph of lower posterior teeth, Test Dataset 8: Digital radiograph of upper posterior teeth, Test Dataset 9: Digital radiograph of lower anterior teeth, Test Dataset 10: Digital radiograph of lower anterior teeth, Test Dataset 11: Digital radiograph of lower posterior teeth, Test Dataset 12: Digital radiograph of lower anterior teeth, Test Dataset 13: Digital radiograph of upper posterior teeth, Test Dataset 14: Digital radiograph of lower teeth, Test Dataset 15: Digital radiograph of lower deciduous teeth, Test Dataset 16: Digital radiograph of lower deciduous teeth, Test Dataset 17: Digital radiograph of lower posterior teeth, Test Dataset 18: Digital radiograph of lower deciduous posterior teeth, Test Dataset 19: Digital radiograph of upper posterior teeth, Test Dataset 20: Digital radiograph of lower posterior teeth, Test Dataset 21: Digital radiograph of lower posterior teeth, Test Dataset 22: Digital radiograph of upper posterior teeth, Test Dataset 23: Digital radiograph of upper posterior teeth, Test Dataset 24: Digital radiograph of lower posterior teeth, Test Dataset 25: Digital radiograph of upper posterior teeth, Test Dataset 26: Digital radiograph of lower deciduous posterior teeth, Test Dataset 27: Digital radiograph of lower deciduous posterior teeth, Test Dataset 28: Digital radiograph of lower posterior teeth, Test Dataset 29: Digital radiograph of lower posterior teeth, Test Dataset 30: Digital radiograph of upper deciduous posterior teeth, Test Dataset 31: Digital radiograph of upper anterior teeth, Test Dataset 32: Digital radiograph of lower deciduous posterior teeth, Test Dataset 33: Digital radiograph of upper anterior teeth, Test Dataset 34: Digital radiograph of lower deciduous posterior teeth, Test Dataset 35: Digital radiograph of lower posterior teeth, Test Dataset 36: Digital radiograph of lower deciduous posterior teeth, Test Dataset 37: Digital radiograph of lower deciduous posterior teeth, Test Dataset 38: Digital radiograph of lower posterior teeth, Test Dataset 39: Digital radiograph of upper posterior teeth, Test Dataset 40: Digital radiograph of lower anterior teeth, Test Dataset 41: Digital radiograph of upper anterior teeth, Test Dataset 42: Digital radiograph of lower posterior teeth, Test Dataset 43: Digital radiograph of lower posterior teeth, Test Dataset 44: Digital radiograph of upper posterior teeth, Test Dataset 45: Digital radiograph of upper anterior teeth, Test Dataset 46: Digital radiograph of upper posterior teeth, Test Dataset 47: Digital radiograph of lower deciduous posterior teeth, Test Dataset 48: Digital radiograph of upper deciduous posterior teeth, Test Dataset 49: Digital radiograph of lower posterior teeth, Test Dataset 50: Digital radiograph of lower deciduous posterior teeth. 

**Additional file 2: **STARD 2015 Guidelines.

**Additional file 3: **Artificial intelligence in dental research: Checklist for authors, reviewers, readers.

**Additional file 4.**

## Data Availability

Part of the test dataset is included in the supplementary files to allow the replication of the results. MSc was a trial model created by Smile with Confidence (SWC) Company to test the ability of AI to detect exposed and unexposed pulp. We are showing the results of a tool that is not available commercially to show that AI could be used to support the work of dentists. As a company specialized in AI, we were testing the power of this technology.
