# Supplementary figures and images for: Detection of the pathological exposure of pulp using an artificial intelligence tool: a multicentric study over periapical radiographs
Source: BMC Oral Health. 2023 Aug 11;23:553. doi: 10.1186/s12903-023-03251-0 (PMC10416487; doi:10.1186/s12903-023-03251-0)

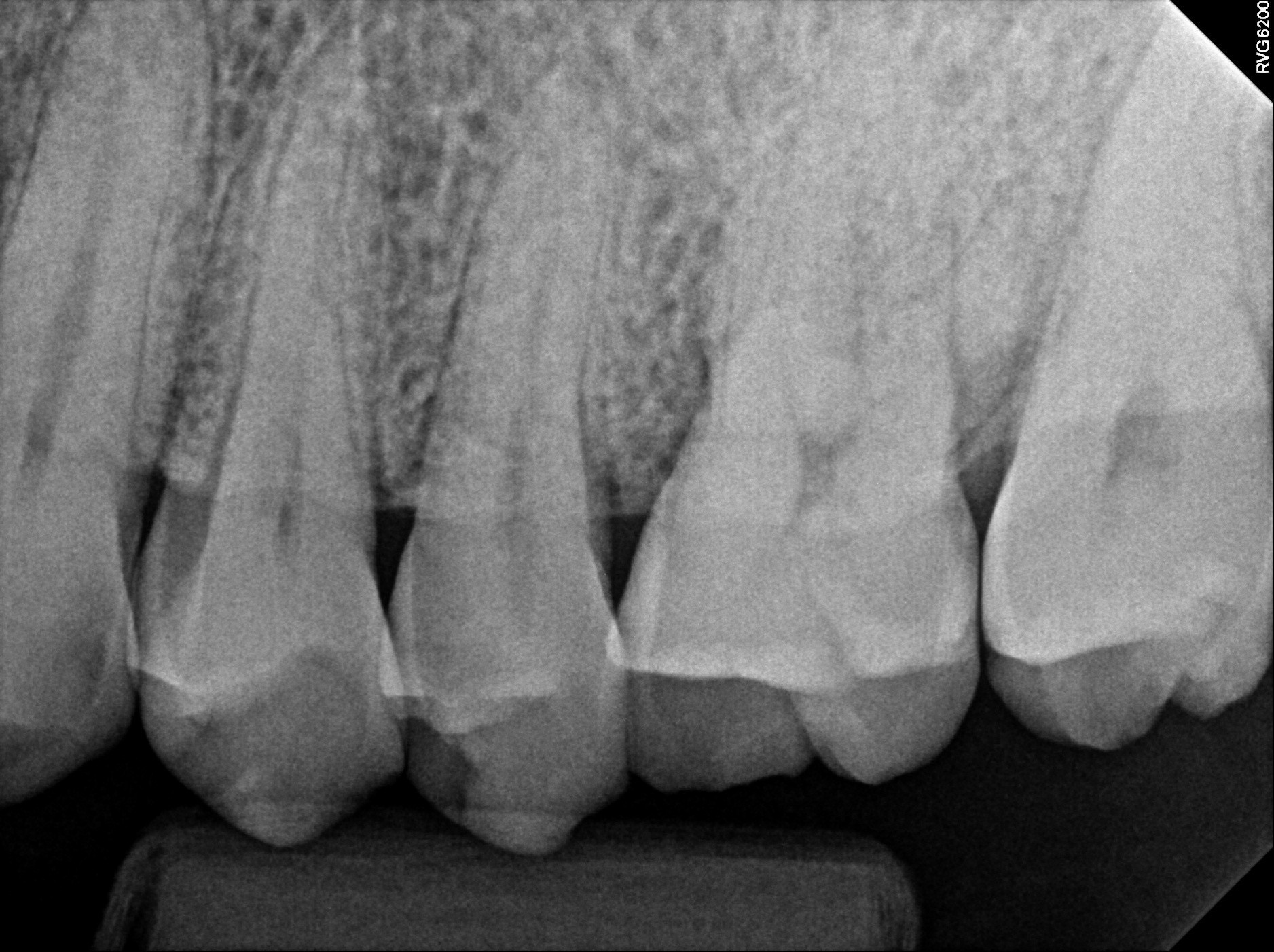

Supplement: Supplementary file 1 — Additional file 1: Test Dataset 1: Digital radiograph of upper posterior teeth. Test Dataset 2: Digital radiograph of upper posterior teeth, Test Dataset 3: Digital radiograph of upper posterior teeth, Test Dataset 4: Digital radiograph of upper posterior teeth, Test Dataset 5: Digital radiograph of upper anterior teeth, Test Dataset 6: Digital radiograph of upper anterior teeth, Test Dataset 7: Digital radiograph of lower posterior teeth, Test Dataset 8: Digital radiograph of upper posterior teeth, Test Dataset 9: Digital radiograph of lower anterior teeth, Test Dataset 10: Digital radiograph of lower anterior teeth, Test Dataset 11: Digital radiograph of lower posterior teeth, Test Dataset 12: Digital radiograph of lower anterior teeth, Test Dataset 13: Digital radiograph of upper posterior teeth, Test Dataset 14: Digital radiograph of lower teeth, Test Dataset 15: Digital radiograph of lower deciduous teeth, Test Dataset 16: Digital radiograph of lower deciduous teeth, Test Dataset 17: Digital radiograph of lower posterior teeth, Test Dataset 18: Digital radiograph of lower deciduous posterior teeth, Test Dataset 19: Digital radiograph of upper posterior teeth, Test Dataset 20: Digital radiograph of lower posterior teeth, Test Dataset 21: Digital radiograph of lower posterior teeth, Test Dataset 22: Digital radiograph of upper posterior teeth, Test Dataset 23: Digital radiograph of upper posterior teeth, Test Dataset 24: Digital radiograph of lower posterior teeth, Test Dataset 25: Digital radiograph of upper posterior teeth, Test Dataset 26: Digital radiograph of lower deciduous posterior teeth, Test Dataset 27: Digital radiograph of lower deciduous posterior teeth, Test Dataset 28: Digital radiograph of lower posterior teeth, Test Dataset 29: Digital radiograph of lower posterior teeth, Test Dataset 30: Digital radiograph of upper deciduous posterior teeth, Test Dataset 31: Digital radiograph of upper anterior teeth, Test Dataset 32: Digital radiograph of lower [file 12903_2023_3251_MOESM1_ESM.zip › Test Dataset 22.jpg]

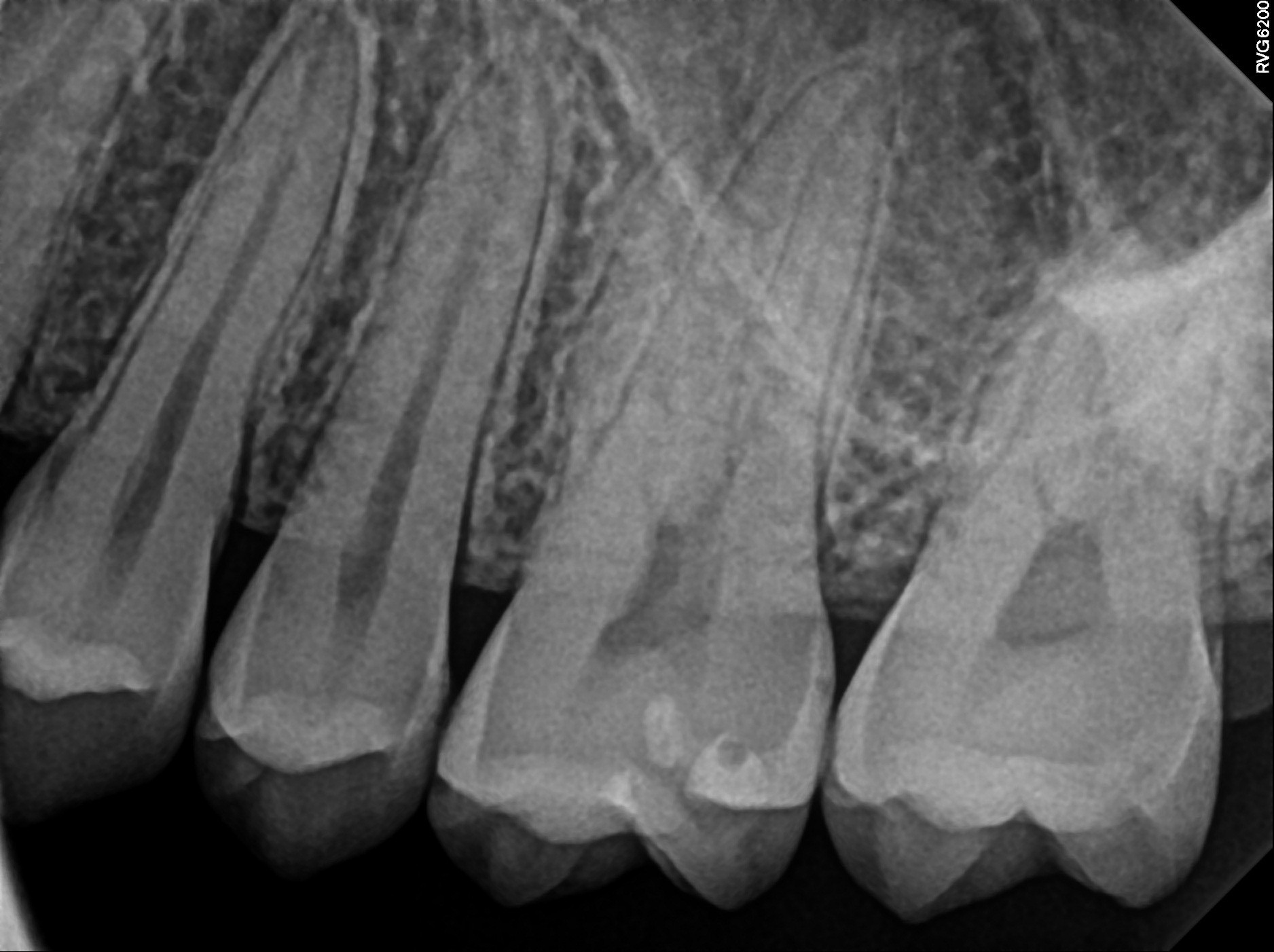

Supplement: Supplementary file 1 — Additional file 1: Test Dataset 1: Digital radiograph of upper posterior teeth. Test Dataset 2: Digital radiograph of upper posterior teeth, Test Dataset 3: Digital radiograph of upper posterior teeth, Test Dataset 4: Digital radiograph of upper posterior teeth, Test Dataset 5: Digital radiograph of upper anterior teeth, Test Dataset 6: Digital radiograph of upper anterior teeth, Test Dataset 7: Digital radiograph of lower posterior teeth, Test Dataset 8: Digital radiograph of upper posterior teeth, Test Dataset 9: Digital radiograph of lower anterior teeth, Test Dataset 10: Digital radiograph of lower anterior teeth, Test Dataset 11: Digital radiograph of lower posterior teeth, Test Dataset 12: Digital radiograph of lower anterior teeth, Test Dataset 13: Digital radiograph of upper posterior teeth, Test Dataset 14: Digital radiograph of lower teeth, Test Dataset 15: Digital radiograph of lower deciduous teeth, Test Dataset 16: Digital radiograph of lower deciduous teeth, Test Dataset 17: Digital radiograph of lower posterior teeth, Test Dataset 18: Digital radiograph of lower deciduous posterior teeth, Test Dataset 19: Digital radiograph of upper posterior teeth, Test Dataset 20: Digital radiograph of lower posterior teeth, Test Dataset 21: Digital radiograph of lower posterior teeth, Test Dataset 22: Digital radiograph of upper posterior teeth, Test Dataset 23: Digital radiograph of upper posterior teeth, Test Dataset 24: Digital radiograph of lower posterior teeth, Test Dataset 25: Digital radiograph of upper posterior teeth, Test Dataset 26: Digital radiograph of lower deciduous posterior teeth, Test Dataset 27: Digital radiograph of lower deciduous posterior teeth, Test Dataset 28: Digital radiograph of lower posterior teeth, Test Dataset 29: Digital radiograph of lower posterior teeth, Test Dataset 30: Digital radiograph of upper deciduous posterior teeth, Test Dataset 31: Digital radiograph of upper anterior teeth, Test Dataset 32: Digital radiograph of lower [file 12903_2023_3251_MOESM1_ESM.zip › Test Dataset 46.jpg]

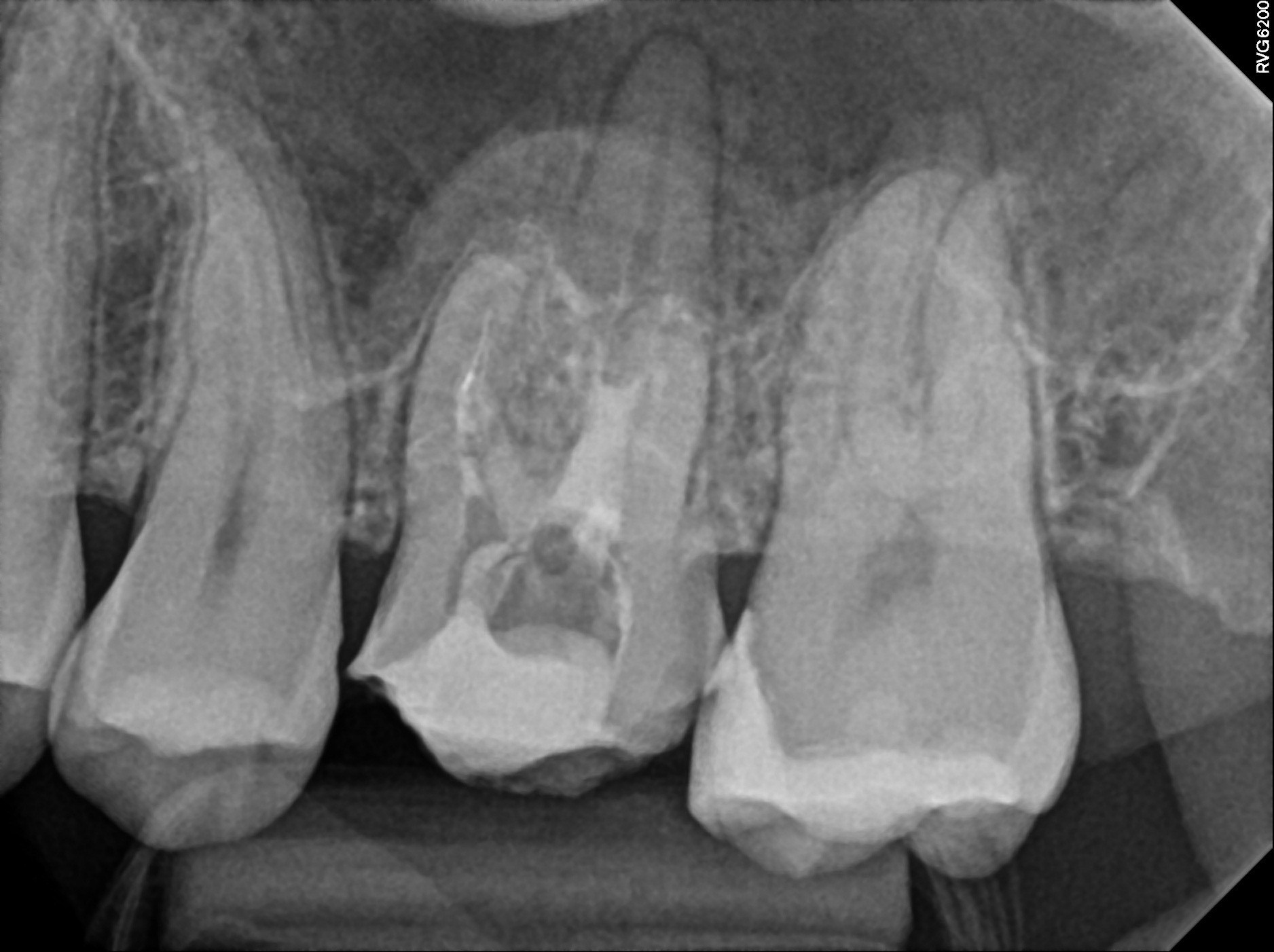

Supplement: Supplementary file 1 — Additional file 1: Test Dataset 1: Digital radiograph of upper posterior teeth. Test Dataset 2: Digital radiograph of upper posterior teeth, Test Dataset 3: Digital radiograph of upper posterior teeth, Test Dataset 4: Digital radiograph of upper posterior teeth, Test Dataset 5: Digital radiograph of upper anterior teeth, Test Dataset 6: Digital radiograph of upper anterior teeth, Test Dataset 7: Digital radiograph of lower posterior teeth, Test Dataset 8: Digital radiograph of upper posterior teeth, Test Dataset 9: Digital radiograph of lower anterior teeth, Test Dataset 10: Digital radiograph of lower anterior teeth, Test Dataset 11: Digital radiograph of lower posterior teeth, Test Dataset 12: Digital radiograph of lower anterior teeth, Test Dataset 13: Digital radiograph of upper posterior teeth, Test Dataset 14: Digital radiograph of lower teeth, Test Dataset 15: Digital radiograph of lower deciduous teeth, Test Dataset 16: Digital radiograph of lower deciduous teeth, Test Dataset 17: Digital radiograph of lower posterior teeth, Test Dataset 18: Digital radiograph of lower deciduous posterior teeth, Test Dataset 19: Digital radiograph of upper posterior teeth, Test Dataset 20: Digital radiograph of lower posterior teeth, Test Dataset 21: Digital radiograph of lower posterior teeth, Test Dataset 22: Digital radiograph of upper posterior teeth, Test Dataset 23: Digital radiograph of upper posterior teeth, Test Dataset 24: Digital radiograph of lower posterior teeth, Test Dataset 25: Digital radiograph of upper posterior teeth, Test Dataset 26: Digital radiograph of lower deciduous posterior teeth, Test Dataset 27: Digital radiograph of lower deciduous posterior teeth, Test Dataset 28: Digital radiograph of lower posterior teeth, Test Dataset 29: Digital radiograph of lower posterior teeth, Test Dataset 30: Digital radiograph of upper deciduous posterior teeth, Test Dataset 31: Digital radiograph of upper anterior teeth, Test Dataset 32: Digital radiograph of lower [file 12903_2023_3251_MOESM1_ESM.zip › Test Dataset 1.jpg]

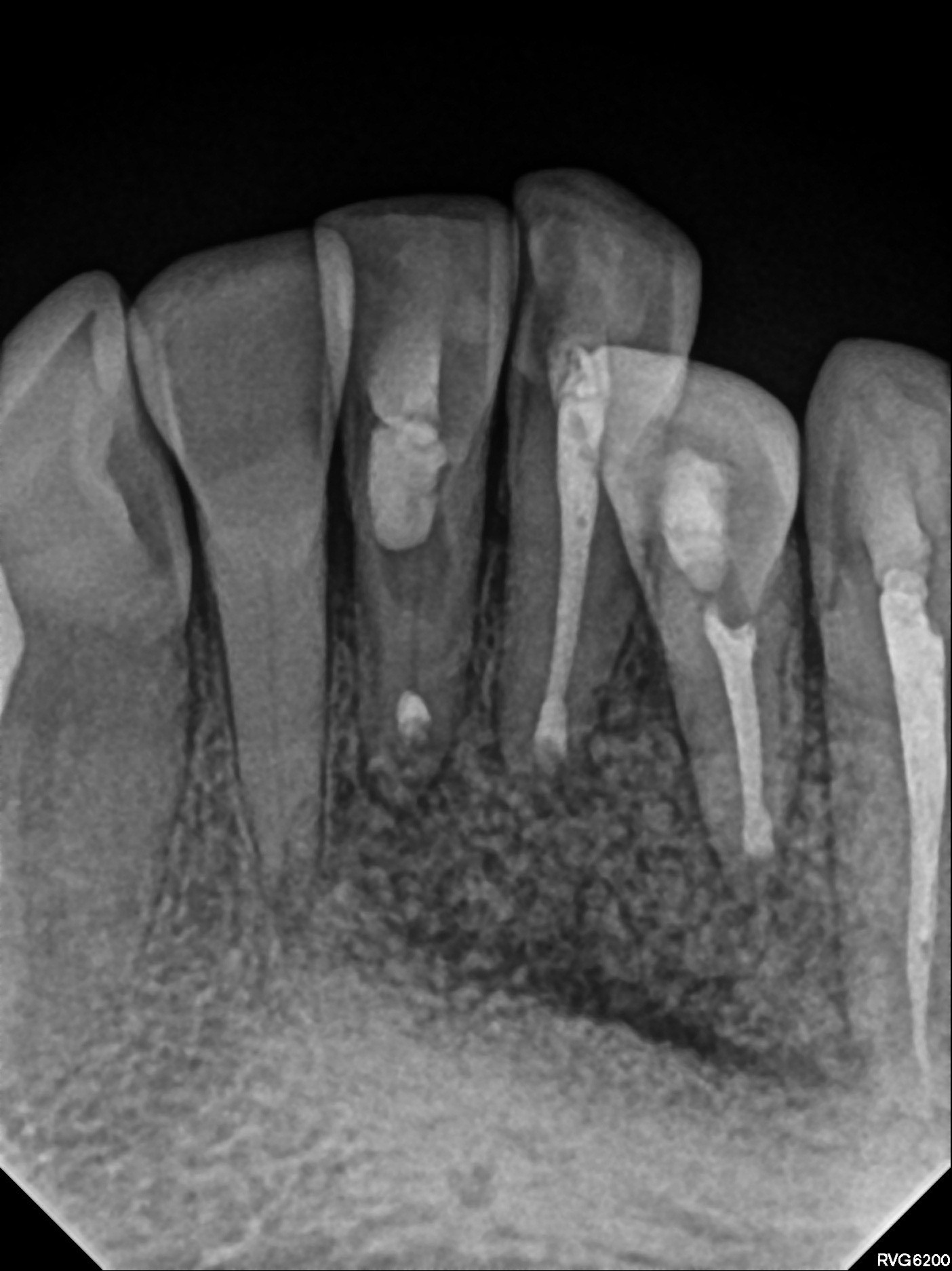

Supplement: Supplementary file 1 — Additional file 1: Test Dataset 1: Digital radiograph of upper posterior teeth. Test Dataset 2: Digital radiograph of upper posterior teeth, Test Dataset 3: Digital radiograph of upper posterior teeth, Test Dataset 4: Digital radiograph of upper posterior teeth, Test Dataset 5: Digital radiograph of upper anterior teeth, Test Dataset 6: Digital radiograph of upper anterior teeth, Test Dataset 7: Digital radiograph of lower posterior teeth, Test Dataset 8: Digital radiograph of upper posterior teeth, Test Dataset 9: Digital radiograph of lower anterior teeth, Test Dataset 10: Digital radiograph of lower anterior teeth, Test Dataset 11: Digital radiograph of lower posterior teeth, Test Dataset 12: Digital radiograph of lower anterior teeth, Test Dataset 13: Digital radiograph of upper posterior teeth, Test Dataset 14: Digital radiograph of lower teeth, Test Dataset 15: Digital radiograph of lower deciduous teeth, Test Dataset 16: Digital radiograph of lower deciduous teeth, Test Dataset 17: Digital radiograph of lower posterior teeth, Test Dataset 18: Digital radiograph of lower deciduous posterior teeth, Test Dataset 19: Digital radiograph of upper posterior teeth, Test Dataset 20: Digital radiograph of lower posterior teeth, Test Dataset 21: Digital radiograph of lower posterior teeth, Test Dataset 22: Digital radiograph of upper posterior teeth, Test Dataset 23: Digital radiograph of upper posterior teeth, Test Dataset 24: Digital radiograph of lower posterior teeth, Test Dataset 25: Digital radiograph of upper posterior teeth, Test Dataset 26: Digital radiograph of lower deciduous posterior teeth, Test Dataset 27: Digital radiograph of lower deciduous posterior teeth, Test Dataset 28: Digital radiograph of lower posterior teeth, Test Dataset 29: Digital radiograph of lower posterior teeth, Test Dataset 30: Digital radiograph of upper deciduous posterior teeth, Test Dataset 31: Digital radiograph of upper anterior teeth, Test Dataset 32: Digital radiograph of lower [file 12903_2023_3251_MOESM1_ESM.zip › Test Dataset 10.jpg]

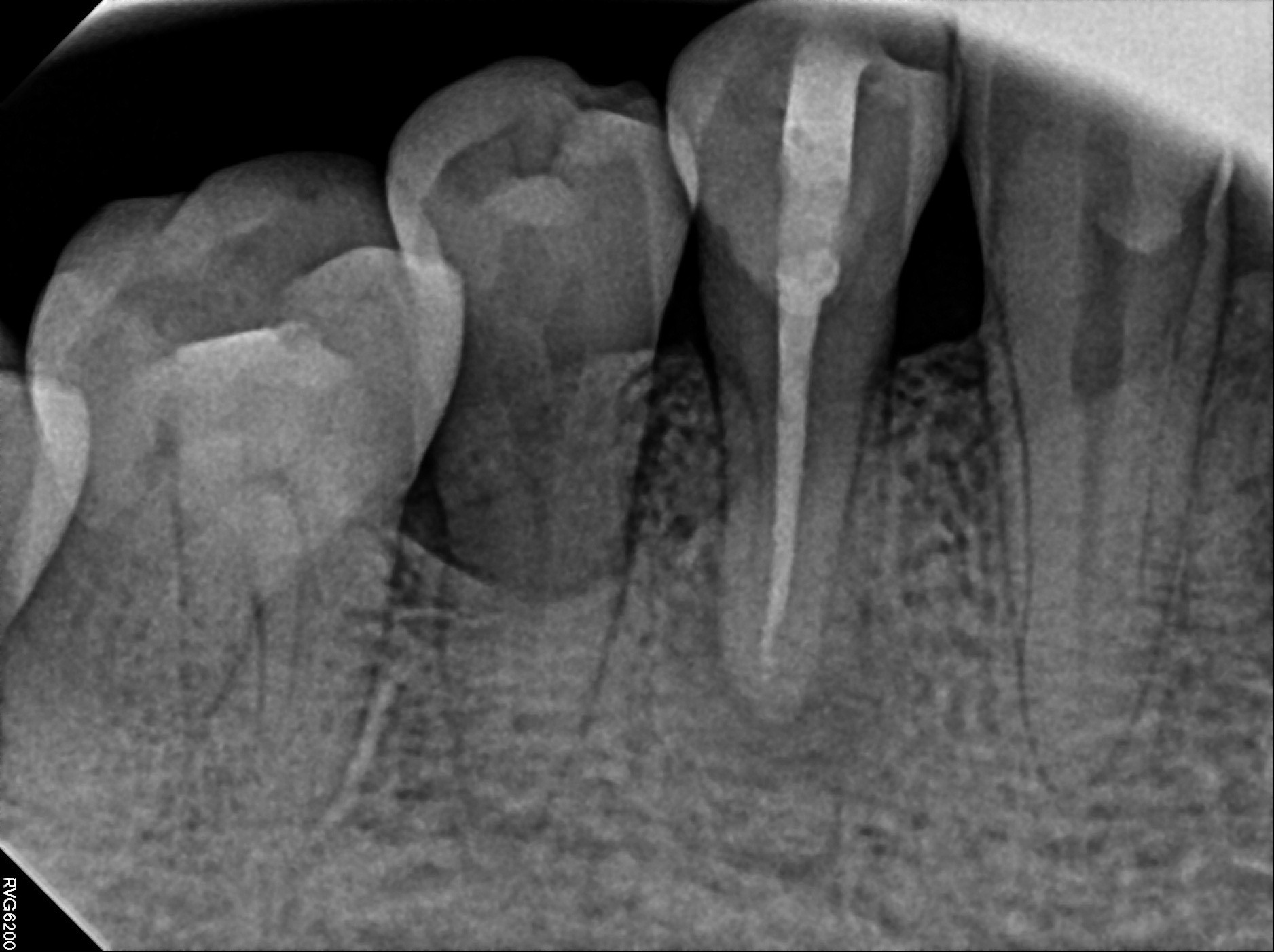

Supplement: Supplementary file 1 — Additional file 1: Test Dataset 1: Digital radiograph of upper posterior teeth. Test Dataset 2: Digital radiograph of upper posterior teeth, Test Dataset 3: Digital radiograph of upper posterior teeth, Test Dataset 4: Digital radiograph of upper posterior teeth, Test Dataset 5: Digital radiograph of upper anterior teeth, Test Dataset 6: Digital radiograph of upper anterior teeth, Test Dataset 7: Digital radiograph of lower posterior teeth, Test Dataset 8: Digital radiograph of upper posterior teeth, Test Dataset 9: Digital radiograph of lower anterior teeth, Test Dataset 10: Digital radiograph of lower anterior teeth, Test Dataset 11: Digital radiograph of lower posterior teeth, Test Dataset 12: Digital radiograph of lower anterior teeth, Test Dataset 13: Digital radiograph of upper posterior teeth, Test Dataset 14: Digital radiograph of lower teeth, Test Dataset 15: Digital radiograph of lower deciduous teeth, Test Dataset 16: Digital radiograph of lower deciduous teeth, Test Dataset 17: Digital radiograph of lower posterior teeth, Test Dataset 18: Digital radiograph of lower deciduous posterior teeth, Test Dataset 19: Digital radiograph of upper posterior teeth, Test Dataset 20: Digital radiograph of lower posterior teeth, Test Dataset 21: Digital radiograph of lower posterior teeth, Test Dataset 22: Digital radiograph of upper posterior teeth, Test Dataset 23: Digital radiograph of upper posterior teeth, Test Dataset 24: Digital radiograph of lower posterior teeth, Test Dataset 25: Digital radiograph of upper posterior teeth, Test Dataset 26: Digital radiograph of lower deciduous posterior teeth, Test Dataset 27: Digital radiograph of lower deciduous posterior teeth, Test Dataset 28: Digital radiograph of lower posterior teeth, Test Dataset 29: Digital radiograph of lower posterior teeth, Test Dataset 30: Digital radiograph of upper deciduous posterior teeth, Test Dataset 31: Digital radiograph of upper anterior teeth, Test Dataset 32: Digital radiograph of lower [file 12903_2023_3251_MOESM1_ESM.zip › Test Dataset 11.jpg]

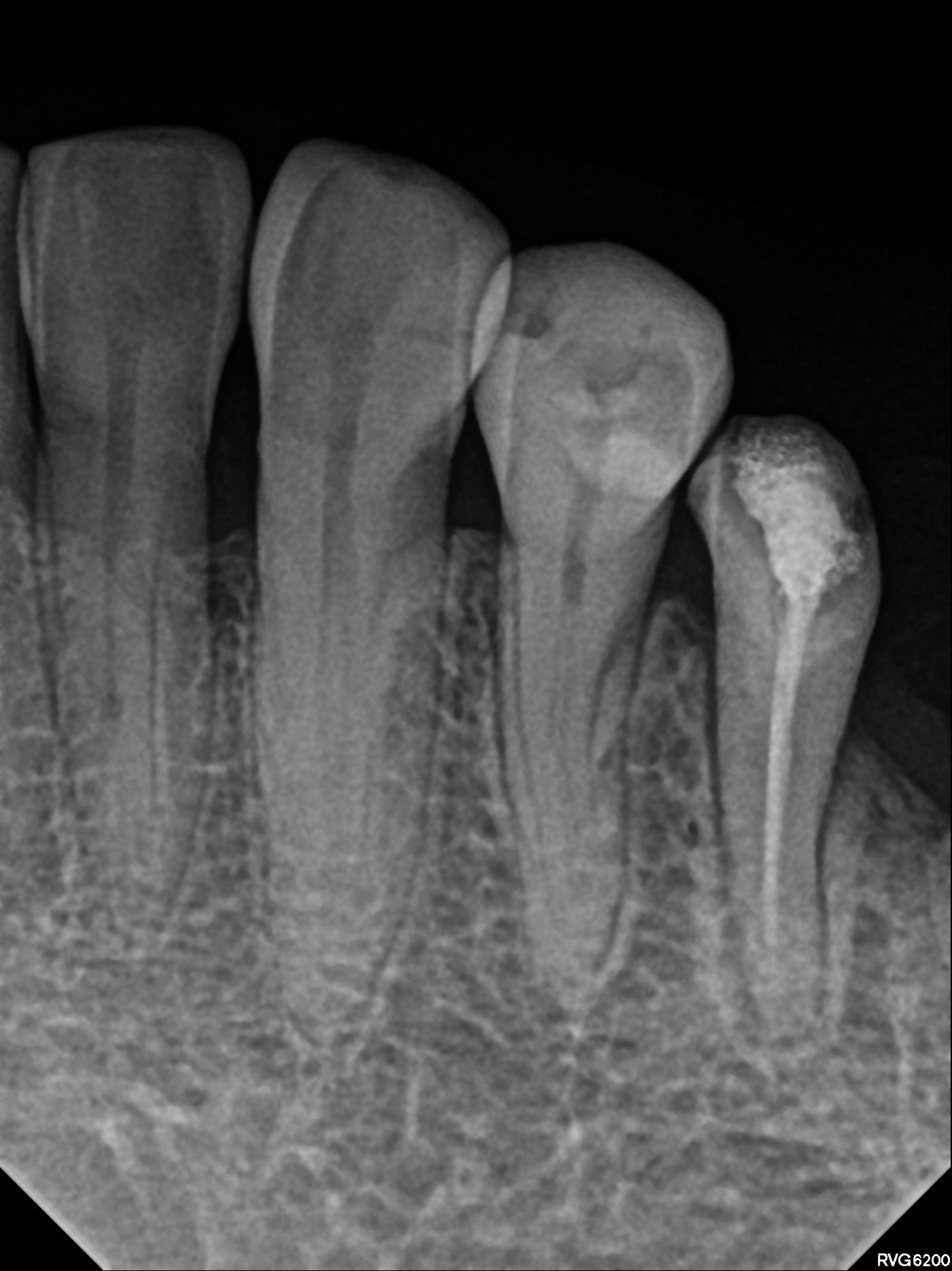

Supplement: Supplementary file 1 — Additional file 1: Test Dataset 1: Digital radiograph of upper posterior teeth. Test Dataset 2: Digital radiograph of upper posterior teeth, Test Dataset 3: Digital radiograph of upper posterior teeth, Test Dataset 4: Digital radiograph of upper posterior teeth, Test Dataset 5: Digital radiograph of upper anterior teeth, Test Dataset 6: Digital radiograph of upper anterior teeth, Test Dataset 7: Digital radiograph of lower posterior teeth, Test Dataset 8: Digital radiograph of upper posterior teeth, Test Dataset 9: Digital radiograph of lower anterior teeth, Test Dataset 10: Digital radiograph of lower anterior teeth, Test Dataset 11: Digital radiograph of lower posterior teeth, Test Dataset 12: Digital radiograph of lower anterior teeth, Test Dataset 13: Digital radiograph of upper posterior teeth, Test Dataset 14: Digital radiograph of lower teeth, Test Dataset 15: Digital radiograph of lower deciduous teeth, Test Dataset 16: Digital radiograph of lower deciduous teeth, Test Dataset 17: Digital radiograph of lower posterior teeth, Test Dataset 18: Digital radiograph of lower deciduous posterior teeth, Test Dataset 19: Digital radiograph of upper posterior teeth, Test Dataset 20: Digital radiograph of lower posterior teeth, Test Dataset 21: Digital radiograph of lower posterior teeth, Test Dataset 22: Digital radiograph of upper posterior teeth, Test Dataset 23: Digital radiograph of upper posterior teeth, Test Dataset 24: Digital radiograph of lower posterior teeth, Test Dataset 25: Digital radiograph of upper posterior teeth, Test Dataset 26: Digital radiograph of lower deciduous posterior teeth, Test Dataset 27: Digital radiograph of lower deciduous posterior teeth, Test Dataset 28: Digital radiograph of lower posterior teeth, Test Dataset 29: Digital radiograph of lower posterior teeth, Test Dataset 30: Digital radiograph of upper deciduous posterior teeth, Test Dataset 31: Digital radiograph of upper anterior teeth, Test Dataset 32: Digital radiograph of lower [file 12903_2023_3251_MOESM1_ESM.zip › Test Dataset 12.jpg]

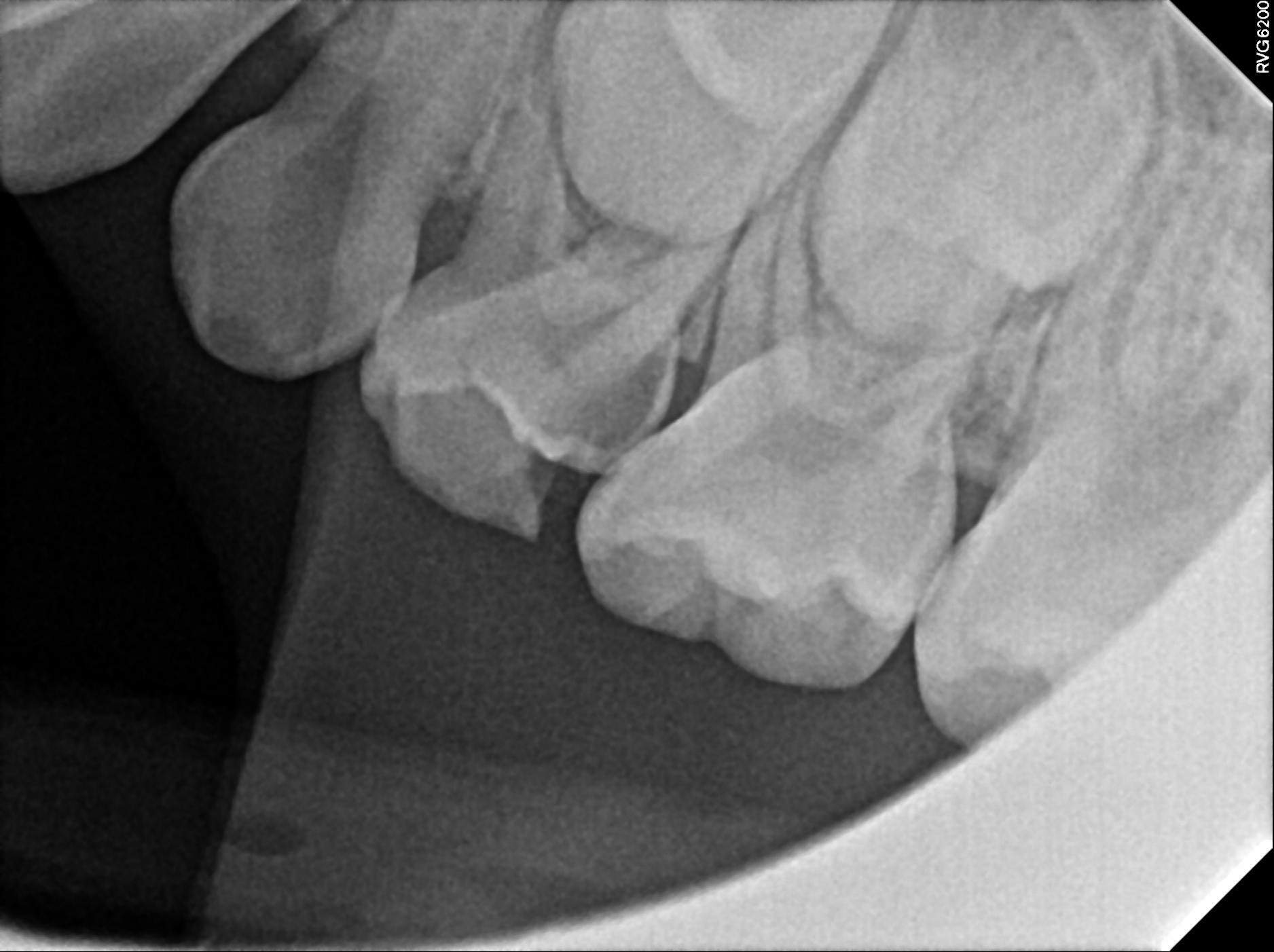

Supplement: Supplementary file 1 — Additional file 1: Test Dataset 1: Digital radiograph of upper posterior teeth. Test Dataset 2: Digital radiograph of upper posterior teeth, Test Dataset 3: Digital radiograph of upper posterior teeth, Test Dataset 4: Digital radiograph of upper posterior teeth, Test Dataset 5: Digital radiograph of upper anterior teeth, Test Dataset 6: Digital radiograph of upper anterior teeth, Test Dataset 7: Digital radiograph of lower posterior teeth, Test Dataset 8: Digital radiograph of upper posterior teeth, Test Dataset 9: Digital radiograph of lower anterior teeth, Test Dataset 10: Digital radiograph of lower anterior teeth, Test Dataset 11: Digital radiograph of lower posterior teeth, Test Dataset 12: Digital radiograph of lower anterior teeth, Test Dataset 13: Digital radiograph of upper posterior teeth, Test Dataset 14: Digital radiograph of lower teeth, Test Dataset 15: Digital radiograph of lower deciduous teeth, Test Dataset 16: Digital radiograph of lower deciduous teeth, Test Dataset 17: Digital radiograph of lower posterior teeth, Test Dataset 18: Digital radiograph of lower deciduous posterior teeth, Test Dataset 19: Digital radiograph of upper posterior teeth, Test Dataset 20: Digital radiograph of lower posterior teeth, Test Dataset 21: Digital radiograph of lower posterior teeth, Test Dataset 22: Digital radiograph of upper posterior teeth, Test Dataset 23: Digital radiograph of upper posterior teeth, Test Dataset 24: Digital radiograph of lower posterior teeth, Test Dataset 25: Digital radiograph of upper posterior teeth, Test Dataset 26: Digital radiograph of lower deciduous posterior teeth, Test Dataset 27: Digital radiograph of lower deciduous posterior teeth, Test Dataset 28: Digital radiograph of lower posterior teeth, Test Dataset 29: Digital radiograph of lower posterior teeth, Test Dataset 30: Digital radiograph of upper deciduous posterior teeth, Test Dataset 31: Digital radiograph of upper anterior teeth, Test Dataset 32: Digital radiograph of lower [file 12903_2023_3251_MOESM1_ESM.zip › Test Dataset 13.jpg]

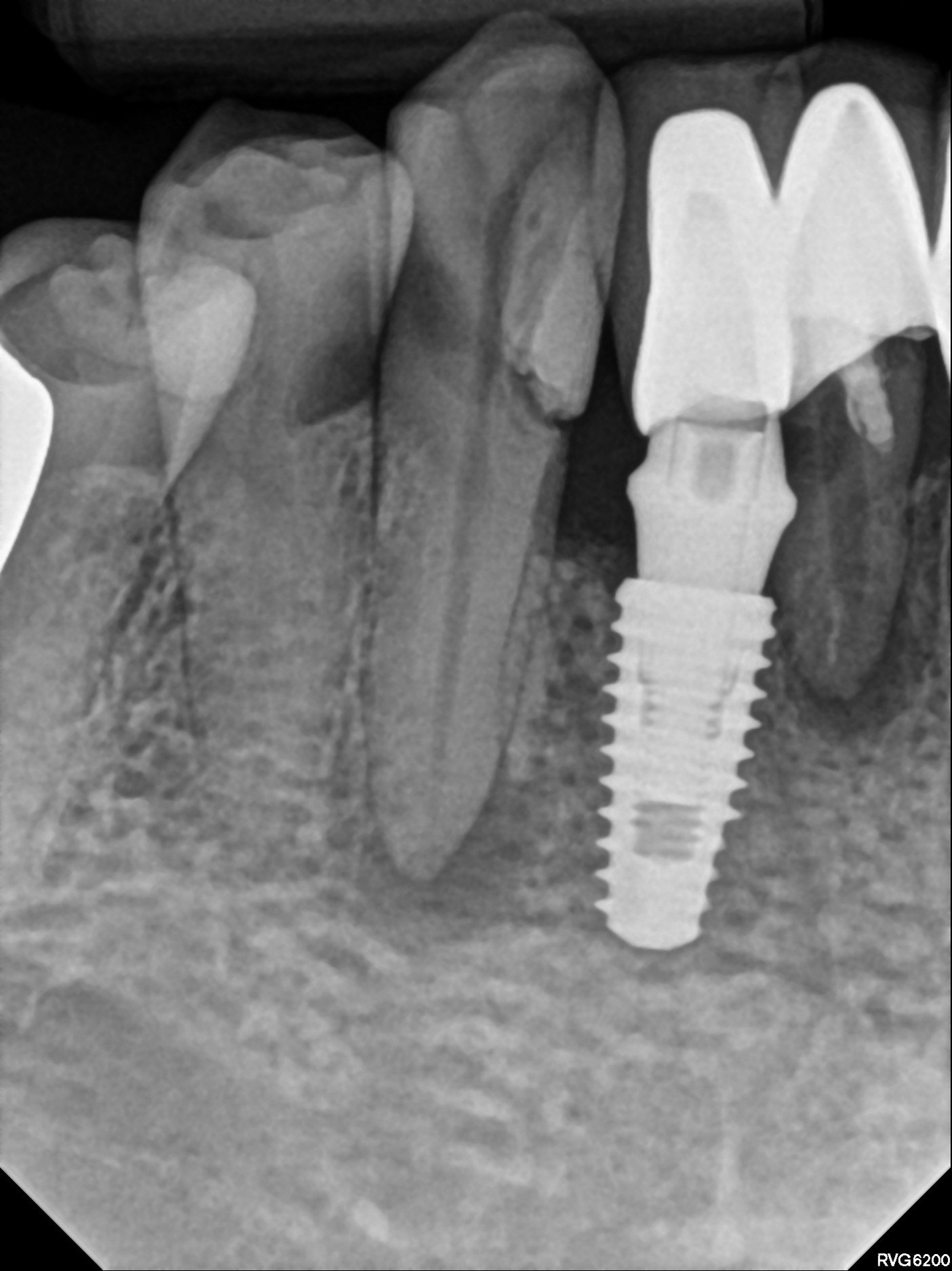

Supplement: Supplementary file 1 — Additional file 1: Test Dataset 1: Digital radiograph of upper posterior teeth. Test Dataset 2: Digital radiograph of upper posterior teeth, Test Dataset 3: Digital radiograph of upper posterior teeth, Test Dataset 4: Digital radiograph of upper posterior teeth, Test Dataset 5: Digital radiograph of upper anterior teeth, Test Dataset 6: Digital radiograph of upper anterior teeth, Test Dataset 7: Digital radiograph of lower posterior teeth, Test Dataset 8: Digital radiograph of upper posterior teeth, Test Dataset 9: Digital radiograph of lower anterior teeth, Test Dataset 10: Digital radiograph of lower anterior teeth, Test Dataset 11: Digital radiograph of lower posterior teeth, Test Dataset 12: Digital radiograph of lower anterior teeth, Test Dataset 13: Digital radiograph of upper posterior teeth, Test Dataset 14: Digital radiograph of lower teeth, Test Dataset 15: Digital radiograph of lower deciduous teeth, Test Dataset 16: Digital radiograph of lower deciduous teeth, Test Dataset 17: Digital radiograph of lower posterior teeth, Test Dataset 18: Digital radiograph of lower deciduous posterior teeth, Test Dataset 19: Digital radiograph of upper posterior teeth, Test Dataset 20: Digital radiograph of lower posterior teeth, Test Dataset 21: Digital radiograph of lower posterior teeth, Test Dataset 22: Digital radiograph of upper posterior teeth, Test Dataset 23: Digital radiograph of upper posterior teeth, Test Dataset 24: Digital radiograph of lower posterior teeth, Test Dataset 25: Digital radiograph of upper posterior teeth, Test Dataset 26: Digital radiograph of lower deciduous posterior teeth, Test Dataset 27: Digital radiograph of lower deciduous posterior teeth, Test Dataset 28: Digital radiograph of lower posterior teeth, Test Dataset 29: Digital radiograph of lower posterior teeth, Test Dataset 30: Digital radiograph of upper deciduous posterior teeth, Test Dataset 31: Digital radiograph of upper anterior teeth, Test Dataset 32: Digital radiograph of lower [file 12903_2023_3251_MOESM1_ESM.zip › Test Dataset 14.jpg]

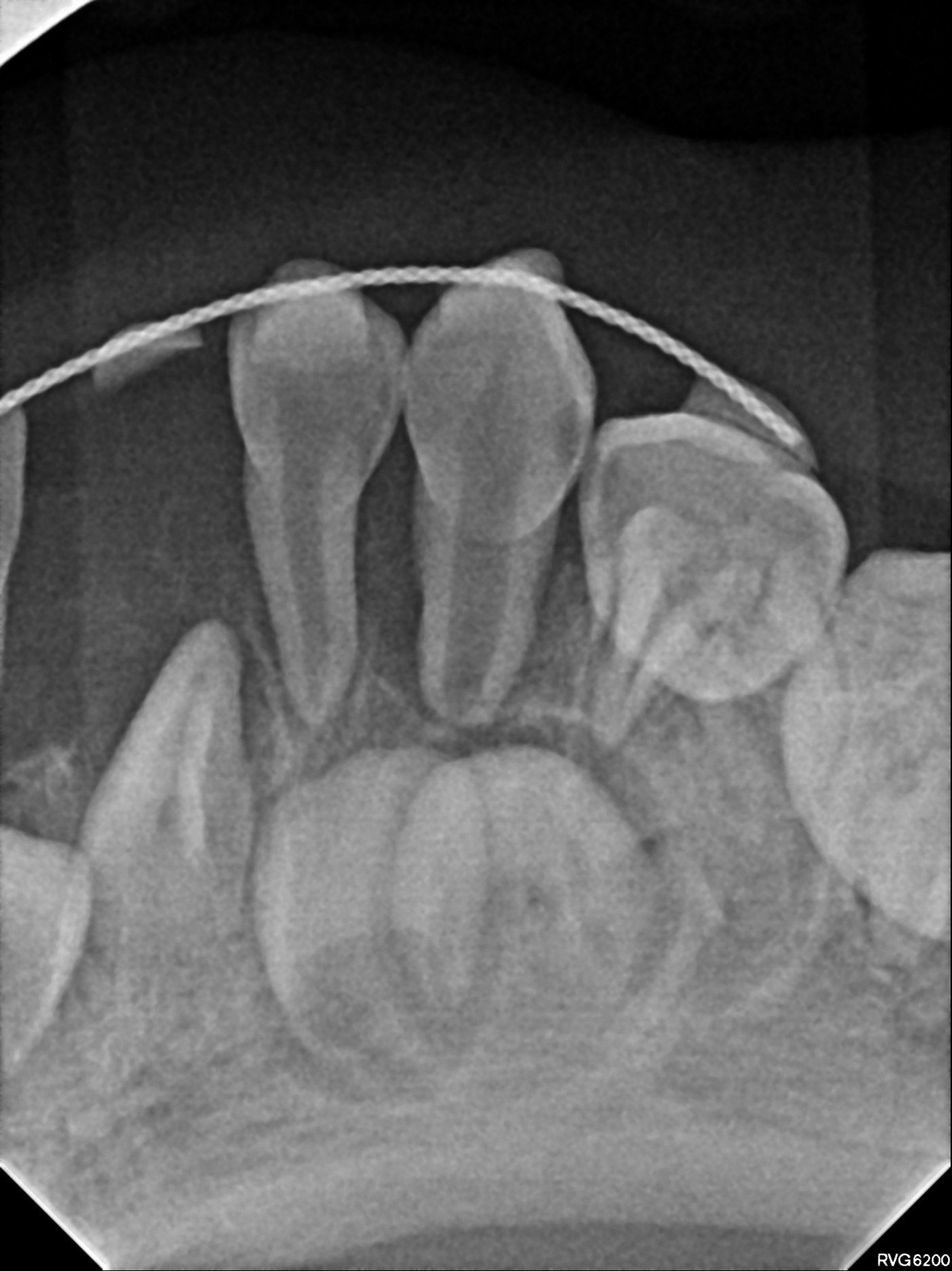

Supplement: Supplementary file 1 — Additional file 1: Test Dataset 1: Digital radiograph of upper posterior teeth. Test Dataset 2: Digital radiograph of upper posterior teeth, Test Dataset 3: Digital radiograph of upper posterior teeth, Test Dataset 4: Digital radiograph of upper posterior teeth, Test Dataset 5: Digital radiograph of upper anterior teeth, Test Dataset 6: Digital radiograph of upper anterior teeth, Test Dataset 7: Digital radiograph of lower posterior teeth, Test Dataset 8: Digital radiograph of upper posterior teeth, Test Dataset 9: Digital radiograph of lower anterior teeth, Test Dataset 10: Digital radiograph of lower anterior teeth, Test Dataset 11: Digital radiograph of lower posterior teeth, Test Dataset 12: Digital radiograph of lower anterior teeth, Test Dataset 13: Digital radiograph of upper posterior teeth, Test Dataset 14: Digital radiograph of lower teeth, Test Dataset 15: Digital radiograph of lower deciduous teeth, Test Dataset 16: Digital radiograph of lower deciduous teeth, Test Dataset 17: Digital radiograph of lower posterior teeth, Test Dataset 18: Digital radiograph of lower deciduous posterior teeth, Test Dataset 19: Digital radiograph of upper posterior teeth, Test Dataset 20: Digital radiograph of lower posterior teeth, Test Dataset 21: Digital radiograph of lower posterior teeth, Test Dataset 22: Digital radiograph of upper posterior teeth, Test Dataset 23: Digital radiograph of upper posterior teeth, Test Dataset 24: Digital radiograph of lower posterior teeth, Test Dataset 25: Digital radiograph of upper posterior teeth, Test Dataset 26: Digital radiograph of lower deciduous posterior teeth, Test Dataset 27: Digital radiograph of lower deciduous posterior teeth, Test Dataset 28: Digital radiograph of lower posterior teeth, Test Dataset 29: Digital radiograph of lower posterior teeth, Test Dataset 30: Digital radiograph of upper deciduous posterior teeth, Test Dataset 31: Digital radiograph of upper anterior teeth, Test Dataset 32: Digital radiograph of lower [file 12903_2023_3251_MOESM1_ESM.zip › Test Dataset 15.jpg]

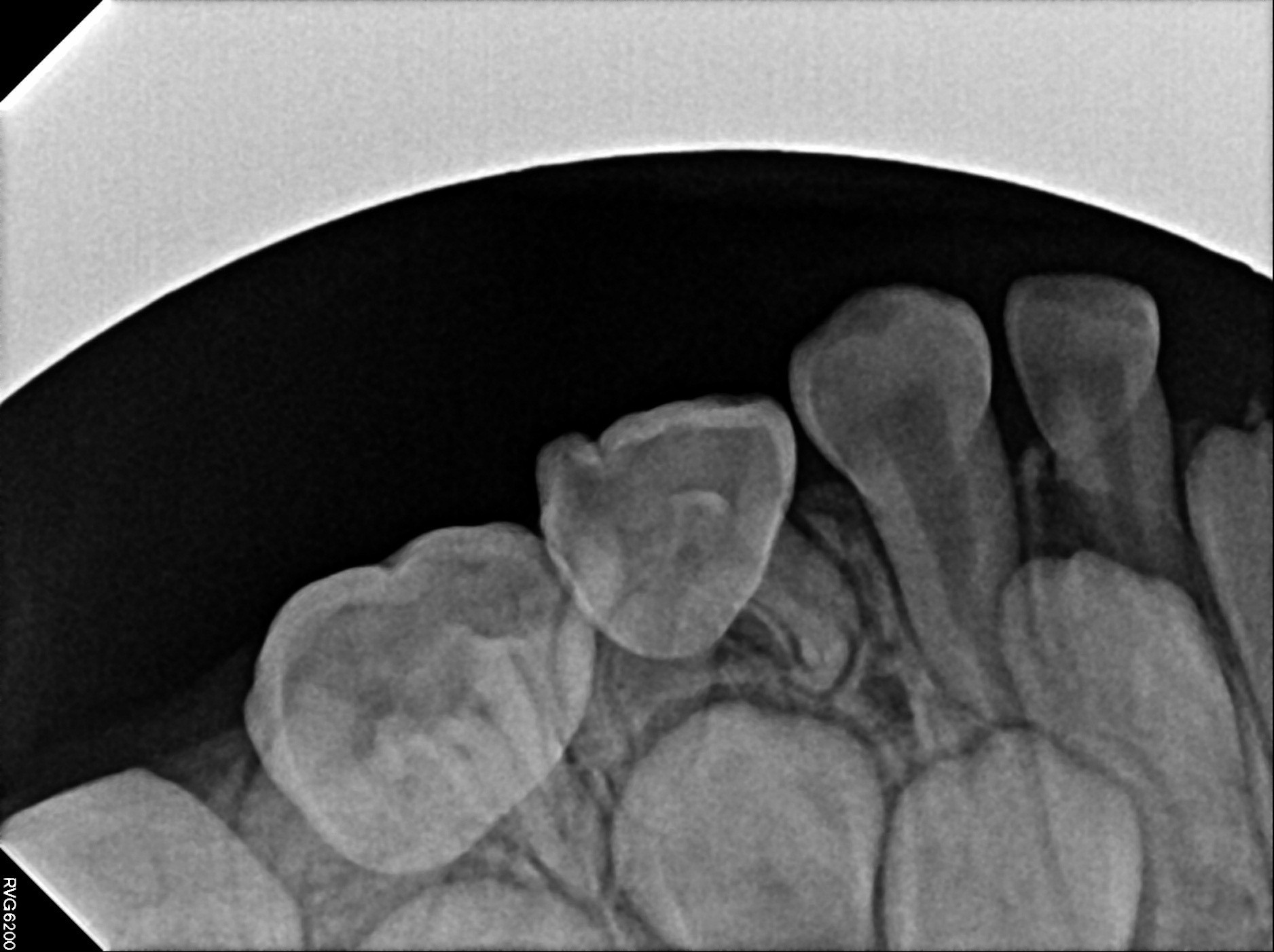

Supplement: Supplementary file 1 — Additional file 1: Test Dataset 1: Digital radiograph of upper posterior teeth. Test Dataset 2: Digital radiograph of upper posterior teeth, Test Dataset 3: Digital radiograph of upper posterior teeth, Test Dataset 4: Digital radiograph of upper posterior teeth, Test Dataset 5: Digital radiograph of upper anterior teeth, Test Dataset 6: Digital radiograph of upper anterior teeth, Test Dataset 7: Digital radiograph of lower posterior teeth, Test Dataset 8: Digital radiograph of upper posterior teeth, Test Dataset 9: Digital radiograph of lower anterior teeth, Test Dataset 10: Digital radiograph of lower anterior teeth, Test Dataset 11: Digital radiograph of lower posterior teeth, Test Dataset 12: Digital radiograph of lower anterior teeth, Test Dataset 13: Digital radiograph of upper posterior teeth, Test Dataset 14: Digital radiograph of lower teeth, Test Dataset 15: Digital radiograph of lower deciduous teeth, Test Dataset 16: Digital radiograph of lower deciduous teeth, Test Dataset 17: Digital radiograph of lower posterior teeth, Test Dataset 18: Digital radiograph of lower deciduous posterior teeth, Test Dataset 19: Digital radiograph of upper posterior teeth, Test Dataset 20: Digital radiograph of lower posterior teeth, Test Dataset 21: Digital radiograph of lower posterior teeth, Test Dataset 22: Digital radiograph of upper posterior teeth, Test Dataset 23: Digital radiograph of upper posterior teeth, Test Dataset 24: Digital radiograph of lower posterior teeth, Test Dataset 25: Digital radiograph of upper posterior teeth, Test Dataset 26: Digital radiograph of lower deciduous posterior teeth, Test Dataset 27: Digital radiograph of lower deciduous posterior teeth, Test Dataset 28: Digital radiograph of lower posterior teeth, Test Dataset 29: Digital radiograph of lower posterior teeth, Test Dataset 30: Digital radiograph of upper deciduous posterior teeth, Test Dataset 31: Digital radiograph of upper anterior teeth, Test Dataset 32: Digital radiograph of lower [file 12903_2023_3251_MOESM1_ESM.zip › Test Dataset 16.jpg]

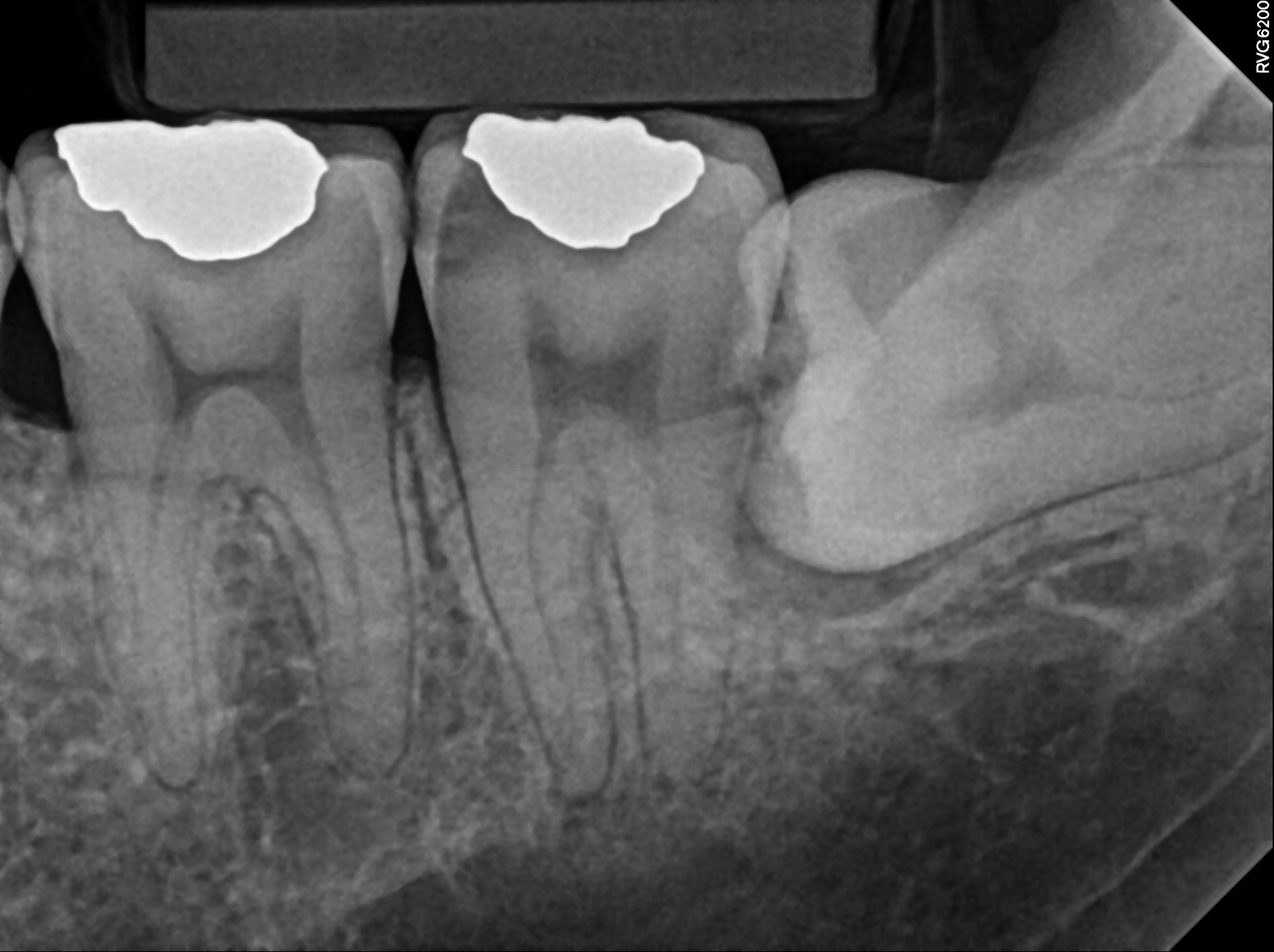

Supplement: Supplementary file 1 — Additional file 1: Test Dataset 1: Digital radiograph of upper posterior teeth. Test Dataset 2: Digital radiograph of upper posterior teeth, Test Dataset 3: Digital radiograph of upper posterior teeth, Test Dataset 4: Digital radiograph of upper posterior teeth, Test Dataset 5: Digital radiograph of upper anterior teeth, Test Dataset 6: Digital radiograph of upper anterior teeth, Test Dataset 7: Digital radiograph of lower posterior teeth, Test Dataset 8: Digital radiograph of upper posterior teeth, Test Dataset 9: Digital radiograph of lower anterior teeth, Test Dataset 10: Digital radiograph of lower anterior teeth, Test Dataset 11: Digital radiograph of lower posterior teeth, Test Dataset 12: Digital radiograph of lower anterior teeth, Test Dataset 13: Digital radiograph of upper posterior teeth, Test Dataset 14: Digital radiograph of lower teeth, Test Dataset 15: Digital radiograph of lower deciduous teeth, Test Dataset 16: Digital radiograph of lower deciduous teeth, Test Dataset 17: Digital radiograph of lower posterior teeth, Test Dataset 18: Digital radiograph of lower deciduous posterior teeth, Test Dataset 19: Digital radiograph of upper posterior teeth, Test Dataset 20: Digital radiograph of lower posterior teeth, Test Dataset 21: Digital radiograph of lower posterior teeth, Test Dataset 22: Digital radiograph of upper posterior teeth, Test Dataset 23: Digital radiograph of upper posterior teeth, Test Dataset 24: Digital radiograph of lower posterior teeth, Test Dataset 25: Digital radiograph of upper posterior teeth, Test Dataset 26: Digital radiograph of lower deciduous posterior teeth, Test Dataset 27: Digital radiograph of lower deciduous posterior teeth, Test Dataset 28: Digital radiograph of lower posterior teeth, Test Dataset 29: Digital radiograph of lower posterior teeth, Test Dataset 30: Digital radiograph of upper deciduous posterior teeth, Test Dataset 31: Digital radiograph of upper anterior teeth, Test Dataset 32: Digital radiograph of lower [file 12903_2023_3251_MOESM1_ESM.zip › Test Dataset 17.jpg]

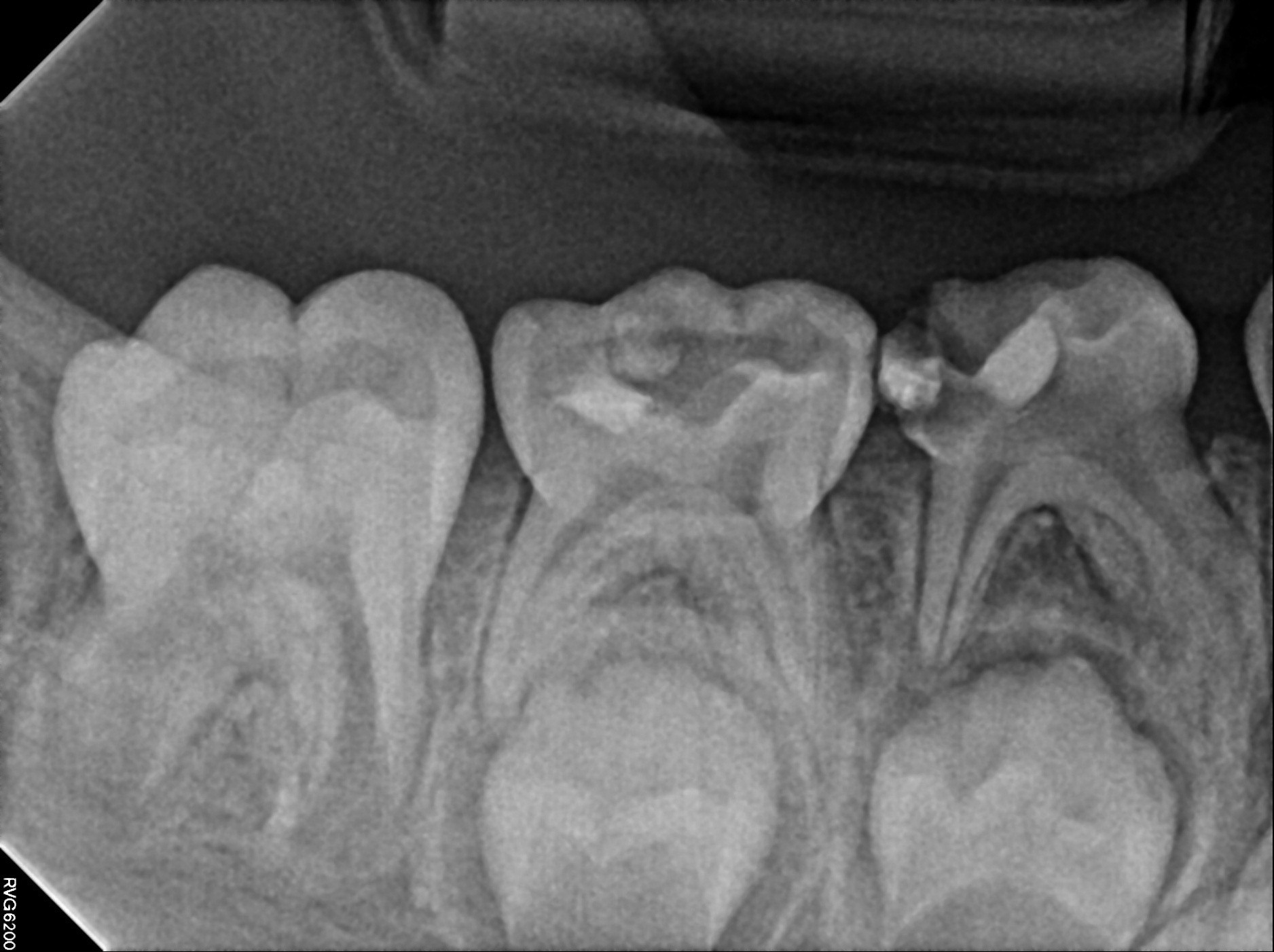

Supplement: Supplementary file 1 — Additional file 1: Test Dataset 1: Digital radiograph of upper posterior teeth. Test Dataset 2: Digital radiograph of upper posterior teeth, Test Dataset 3: Digital radiograph of upper posterior teeth, Test Dataset 4: Digital radiograph of upper posterior teeth, Test Dataset 5: Digital radiograph of upper anterior teeth, Test Dataset 6: Digital radiograph of upper anterior teeth, Test Dataset 7: Digital radiograph of lower posterior teeth, Test Dataset 8: Digital radiograph of upper posterior teeth, Test Dataset 9: Digital radiograph of lower anterior teeth, Test Dataset 10: Digital radiograph of lower anterior teeth, Test Dataset 11: Digital radiograph of lower posterior teeth, Test Dataset 12: Digital radiograph of lower anterior teeth, Test Dataset 13: Digital radiograph of upper posterior teeth, Test Dataset 14: Digital radiograph of lower teeth, Test Dataset 15: Digital radiograph of lower deciduous teeth, Test Dataset 16: Digital radiograph of lower deciduous teeth, Test Dataset 17: Digital radiograph of lower posterior teeth, Test Dataset 18: Digital radiograph of lower deciduous posterior teeth, Test Dataset 19: Digital radiograph of upper posterior teeth, Test Dataset 20: Digital radiograph of lower posterior teeth, Test Dataset 21: Digital radiograph of lower posterior teeth, Test Dataset 22: Digital radiograph of upper posterior teeth, Test Dataset 23: Digital radiograph of upper posterior teeth, Test Dataset 24: Digital radiograph of lower posterior teeth, Test Dataset 25: Digital radiograph of upper posterior teeth, Test Dataset 26: Digital radiograph of lower deciduous posterior teeth, Test Dataset 27: Digital radiograph of lower deciduous posterior teeth, Test Dataset 28: Digital radiograph of lower posterior teeth, Test Dataset 29: Digital radiograph of lower posterior teeth, Test Dataset 30: Digital radiograph of upper deciduous posterior teeth, Test Dataset 31: Digital radiograph of upper anterior teeth, Test Dataset 32: Digital radiograph of lower [file 12903_2023_3251_MOESM1_ESM.zip › Test Dataset 18.jpg]

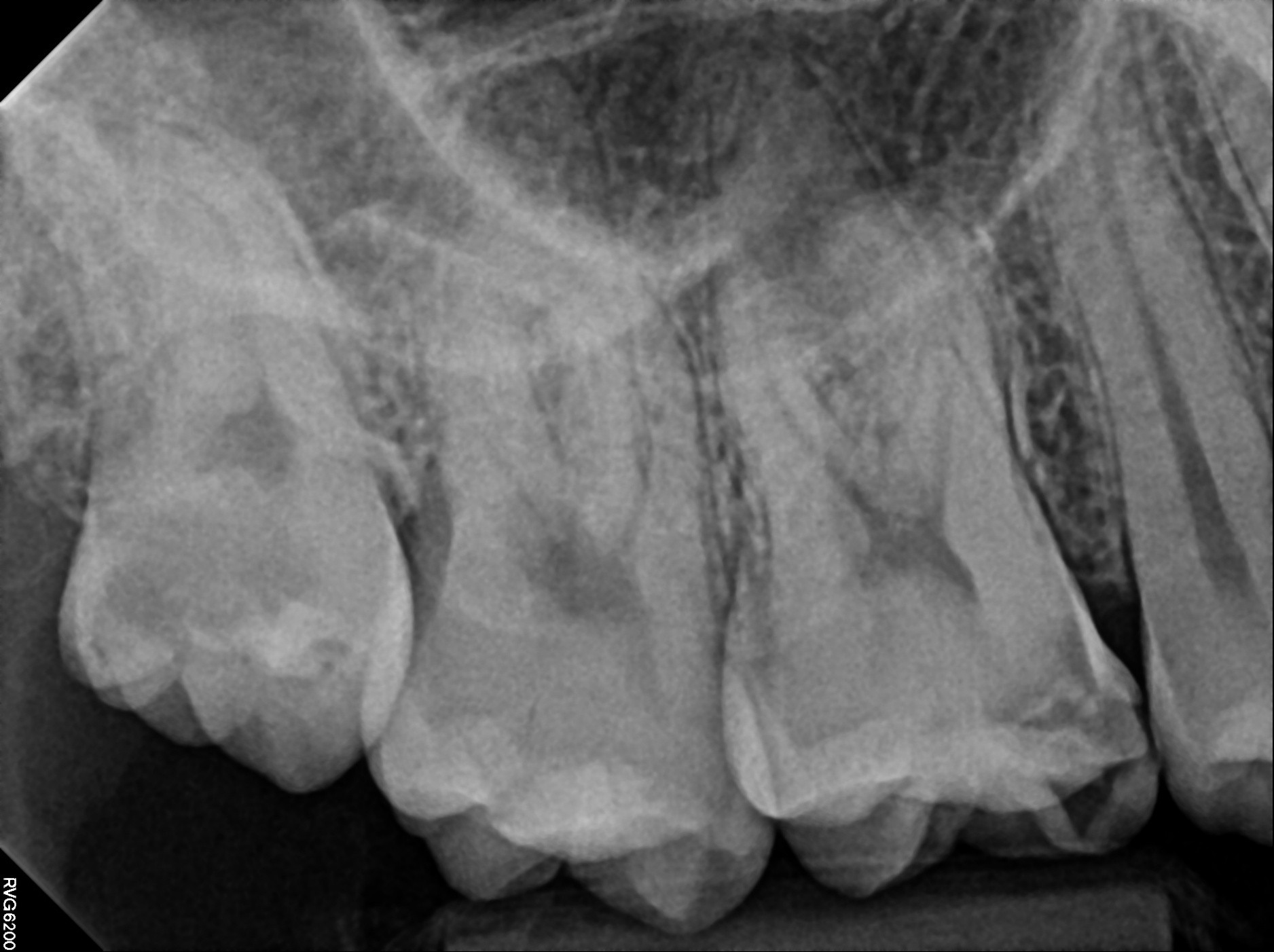

Supplement: Supplementary file 1 — Additional file 1: Test Dataset 1: Digital radiograph of upper posterior teeth. Test Dataset 2: Digital radiograph of upper posterior teeth, Test Dataset 3: Digital radiograph of upper posterior teeth, Test Dataset 4: Digital radiograph of upper posterior teeth, Test Dataset 5: Digital radiograph of upper anterior teeth, Test Dataset 6: Digital radiograph of upper anterior teeth, Test Dataset 7: Digital radiograph of lower posterior teeth, Test Dataset 8: Digital radiograph of upper posterior teeth, Test Dataset 9: Digital radiograph of lower anterior teeth, Test Dataset 10: Digital radiograph of lower anterior teeth, Test Dataset 11: Digital radiograph of lower posterior teeth, Test Dataset 12: Digital radiograph of lower anterior teeth, Test Dataset 13: Digital radiograph of upper posterior teeth, Test Dataset 14: Digital radiograph of lower teeth, Test Dataset 15: Digital radiograph of lower deciduous teeth, Test Dataset 16: Digital radiograph of lower deciduous teeth, Test Dataset 17: Digital radiograph of lower posterior teeth, Test Dataset 18: Digital radiograph of lower deciduous posterior teeth, Test Dataset 19: Digital radiograph of upper posterior teeth, Test Dataset 20: Digital radiograph of lower posterior teeth, Test Dataset 21: Digital radiograph of lower posterior teeth, Test Dataset 22: Digital radiograph of upper posterior teeth, Test Dataset 23: Digital radiograph of upper posterior teeth, Test Dataset 24: Digital radiograph of lower posterior teeth, Test Dataset 25: Digital radiograph of upper posterior teeth, Test Dataset 26: Digital radiograph of lower deciduous posterior teeth, Test Dataset 27: Digital radiograph of lower deciduous posterior teeth, Test Dataset 28: Digital radiograph of lower posterior teeth, Test Dataset 29: Digital radiograph of lower posterior teeth, Test Dataset 30: Digital radiograph of upper deciduous posterior teeth, Test Dataset 31: Digital radiograph of upper anterior teeth, Test Dataset 32: Digital radiograph of lower [file 12903_2023_3251_MOESM1_ESM.zip › Test Dataset 19.jpg]

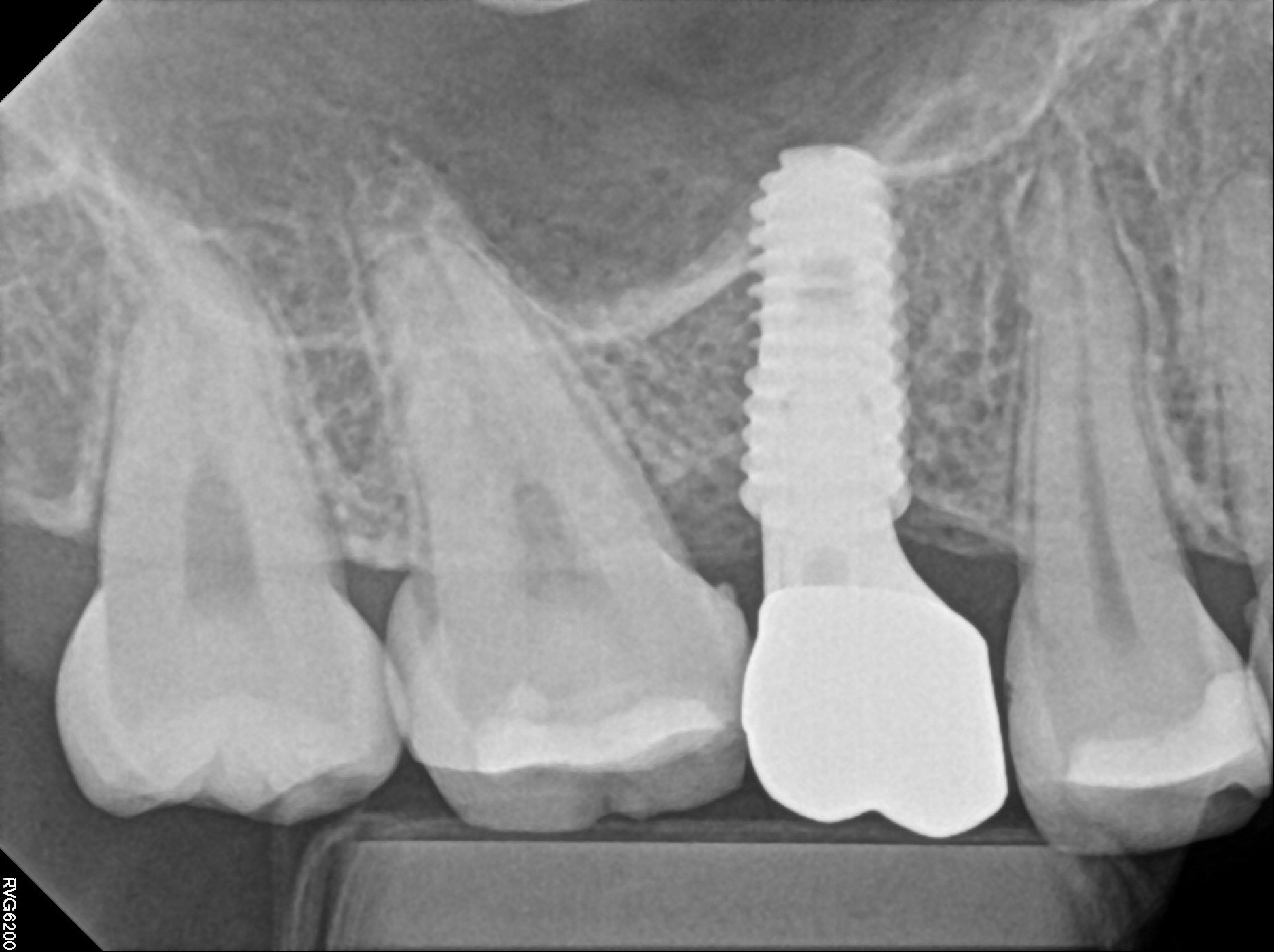

Supplement: Supplementary file 1 — Additional file 1: Test Dataset 1: Digital radiograph of upper posterior teeth. Test Dataset 2: Digital radiograph of upper posterior teeth, Test Dataset 3: Digital radiograph of upper posterior teeth, Test Dataset 4: Digital radiograph of upper posterior teeth, Test Dataset 5: Digital radiograph of upper anterior teeth, Test Dataset 6: Digital radiograph of upper anterior teeth, Test Dataset 7: Digital radiograph of lower posterior teeth, Test Dataset 8: Digital radiograph of upper posterior teeth, Test Dataset 9: Digital radiograph of lower anterior teeth, Test Dataset 10: Digital radiograph of lower anterior teeth, Test Dataset 11: Digital radiograph of lower posterior teeth, Test Dataset 12: Digital radiograph of lower anterior teeth, Test Dataset 13: Digital radiograph of upper posterior teeth, Test Dataset 14: Digital radiograph of lower teeth, Test Dataset 15: Digital radiograph of lower deciduous teeth, Test Dataset 16: Digital radiograph of lower deciduous teeth, Test Dataset 17: Digital radiograph of lower posterior teeth, Test Dataset 18: Digital radiograph of lower deciduous posterior teeth, Test Dataset 19: Digital radiograph of upper posterior teeth, Test Dataset 20: Digital radiograph of lower posterior teeth, Test Dataset 21: Digital radiograph of lower posterior teeth, Test Dataset 22: Digital radiograph of upper posterior teeth, Test Dataset 23: Digital radiograph of upper posterior teeth, Test Dataset 24: Digital radiograph of lower posterior teeth, Test Dataset 25: Digital radiograph of upper posterior teeth, Test Dataset 26: Digital radiograph of lower deciduous posterior teeth, Test Dataset 27: Digital radiograph of lower deciduous posterior teeth, Test Dataset 28: Digital radiograph of lower posterior teeth, Test Dataset 29: Digital radiograph of lower posterior teeth, Test Dataset 30: Digital radiograph of upper deciduous posterior teeth, Test Dataset 31: Digital radiograph of upper anterior teeth, Test Dataset 32: Digital radiograph of lower [file 12903_2023_3251_MOESM1_ESM.zip › Test Dataset 2.jpg]

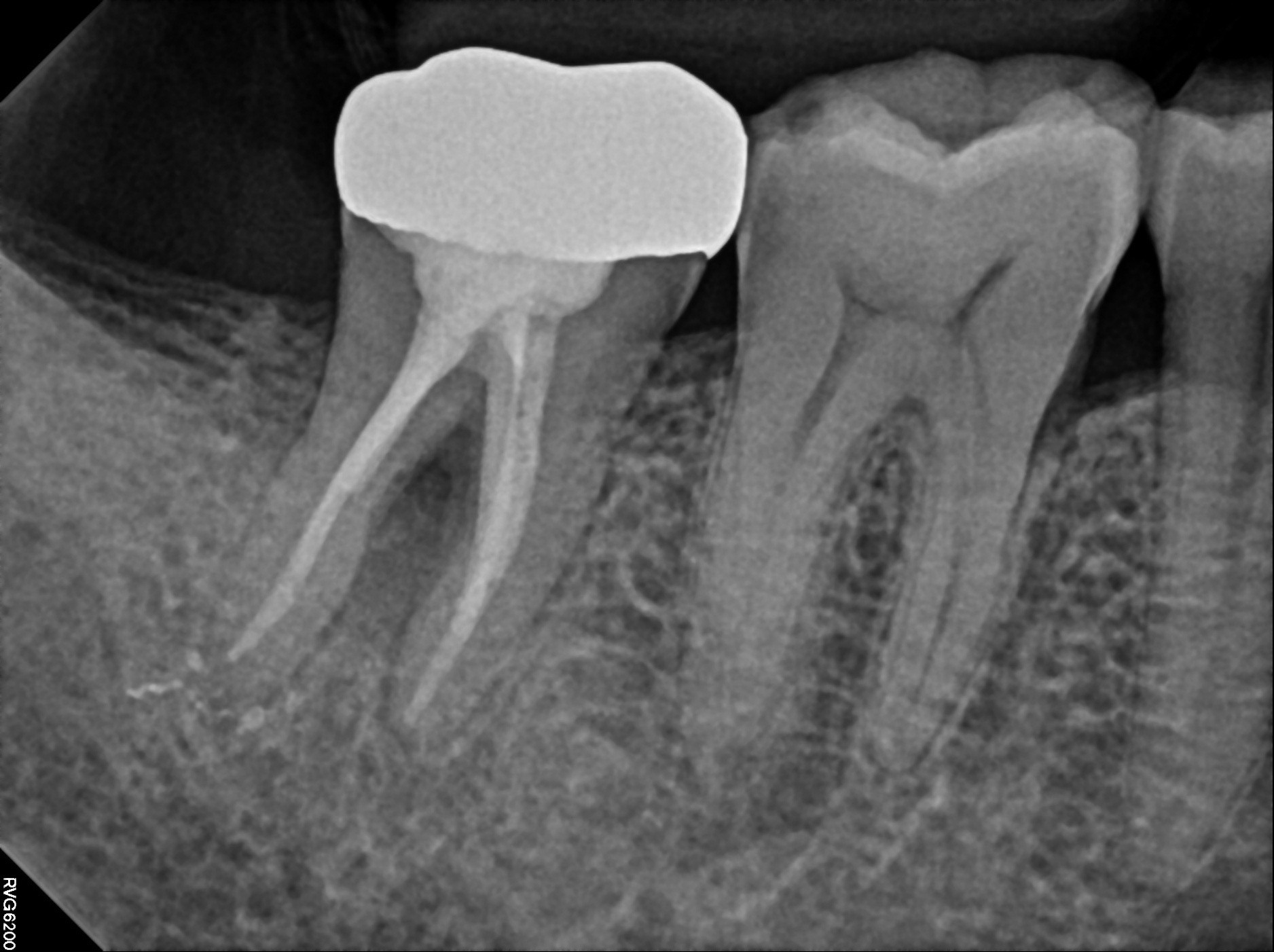

Supplement: Supplementary file 1 — Additional file 1: Test Dataset 1: Digital radiograph of upper posterior teeth. Test Dataset 2: Digital radiograph of upper posterior teeth, Test Dataset 3: Digital radiograph of upper posterior teeth, Test Dataset 4: Digital radiograph of upper posterior teeth, Test Dataset 5: Digital radiograph of upper anterior teeth, Test Dataset 6: Digital radiograph of upper anterior teeth, Test Dataset 7: Digital radiograph of lower posterior teeth, Test Dataset 8: Digital radiograph of upper posterior teeth, Test Dataset 9: Digital radiograph of lower anterior teeth, Test Dataset 10: Digital radiograph of lower anterior teeth, Test Dataset 11: Digital radiograph of lower posterior teeth, Test Dataset 12: Digital radiograph of lower anterior teeth, Test Dataset 13: Digital radiograph of upper posterior teeth, Test Dataset 14: Digital radiograph of lower teeth, Test Dataset 15: Digital radiograph of lower deciduous teeth, Test Dataset 16: Digital radiograph of lower deciduous teeth, Test Dataset 17: Digital radiograph of lower posterior teeth, Test Dataset 18: Digital radiograph of lower deciduous posterior teeth, Test Dataset 19: Digital radiograph of upper posterior teeth, Test Dataset 20: Digital radiograph of lower posterior teeth, Test Dataset 21: Digital radiograph of lower posterior teeth, Test Dataset 22: Digital radiograph of upper posterior teeth, Test Dataset 23: Digital radiograph of upper posterior teeth, Test Dataset 24: Digital radiograph of lower posterior teeth, Test Dataset 25: Digital radiograph of upper posterior teeth, Test Dataset 26: Digital radiograph of lower deciduous posterior teeth, Test Dataset 27: Digital radiograph of lower deciduous posterior teeth, Test Dataset 28: Digital radiograph of lower posterior teeth, Test Dataset 29: Digital radiograph of lower posterior teeth, Test Dataset 30: Digital radiograph of upper deciduous posterior teeth, Test Dataset 31: Digital radiograph of upper anterior teeth, Test Dataset 32: Digital radiograph of lower [file 12903_2023_3251_MOESM1_ESM.zip › Test Dataset 20.jpg]

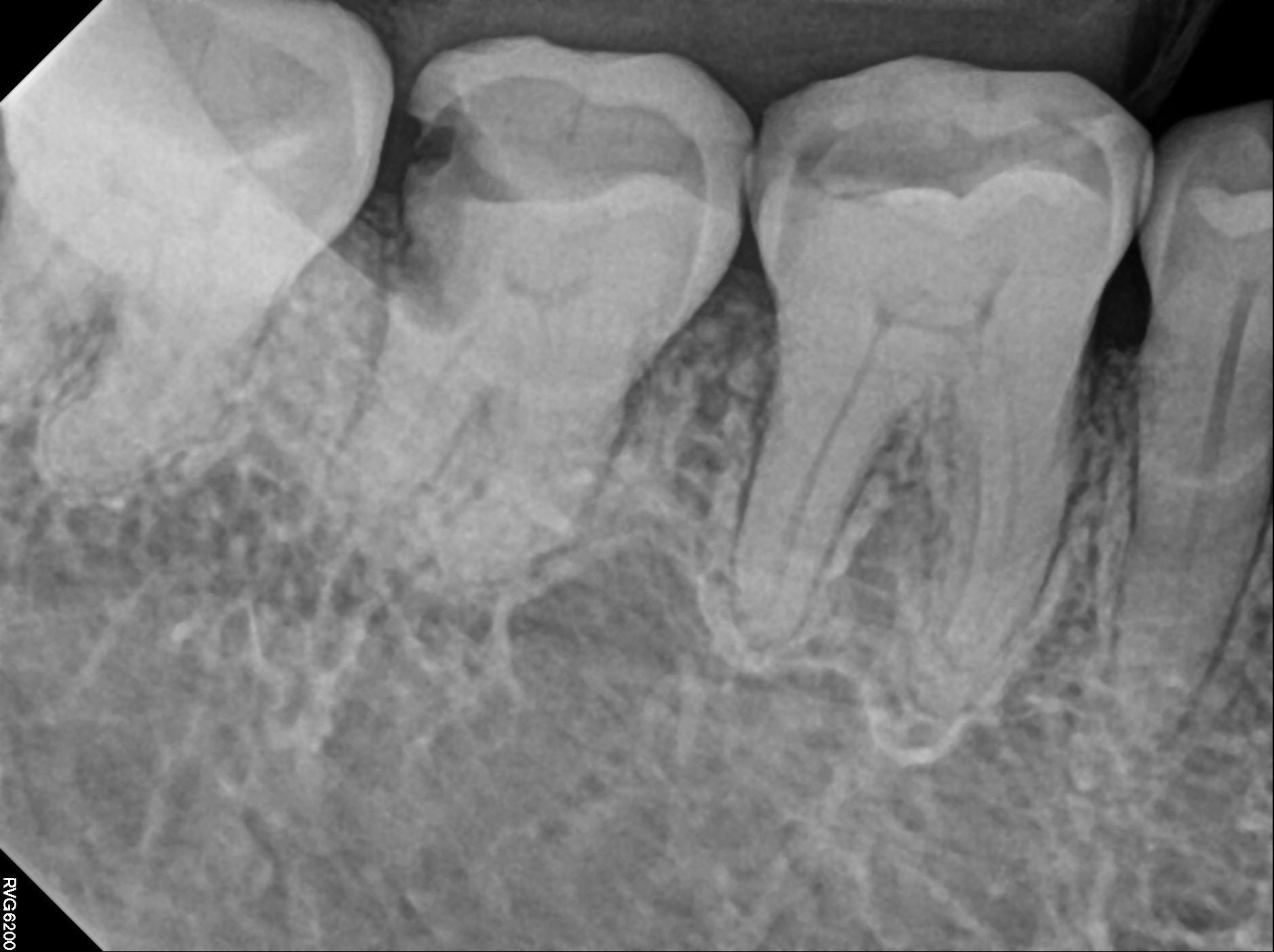

Supplement: Supplementary file 1 — Additional file 1: Test Dataset 1: Digital radiograph of upper posterior teeth. Test Dataset 2: Digital radiograph of upper posterior teeth, Test Dataset 3: Digital radiograph of upper posterior teeth, Test Dataset 4: Digital radiograph of upper posterior teeth, Test Dataset 5: Digital radiograph of upper anterior teeth, Test Dataset 6: Digital radiograph of upper anterior teeth, Test Dataset 7: Digital radiograph of lower posterior teeth, Test Dataset 8: Digital radiograph of upper posterior teeth, Test Dataset 9: Digital radiograph of lower anterior teeth, Test Dataset 10: Digital radiograph of lower anterior teeth, Test Dataset 11: Digital radiograph of lower posterior teeth, Test Dataset 12: Digital radiograph of lower anterior teeth, Test Dataset 13: Digital radiograph of upper posterior teeth, Test Dataset 14: Digital radiograph of lower teeth, Test Dataset 15: Digital radiograph of lower deciduous teeth, Test Dataset 16: Digital radiograph of lower deciduous teeth, Test Dataset 17: Digital radiograph of lower posterior teeth, Test Dataset 18: Digital radiograph of lower deciduous posterior teeth, Test Dataset 19: Digital radiograph of upper posterior teeth, Test Dataset 20: Digital radiograph of lower posterior teeth, Test Dataset 21: Digital radiograph of lower posterior teeth, Test Dataset 22: Digital radiograph of upper posterior teeth, Test Dataset 23: Digital radiograph of upper posterior teeth, Test Dataset 24: Digital radiograph of lower posterior teeth, Test Dataset 25: Digital radiograph of upper posterior teeth, Test Dataset 26: Digital radiograph of lower deciduous posterior teeth, Test Dataset 27: Digital radiograph of lower deciduous posterior teeth, Test Dataset 28: Digital radiograph of lower posterior teeth, Test Dataset 29: Digital radiograph of lower posterior teeth, Test Dataset 30: Digital radiograph of upper deciduous posterior teeth, Test Dataset 31: Digital radiograph of upper anterior teeth, Test Dataset 32: Digital radiograph of lower [file 12903_2023_3251_MOESM1_ESM.zip › Test Dataset 21.jpg]

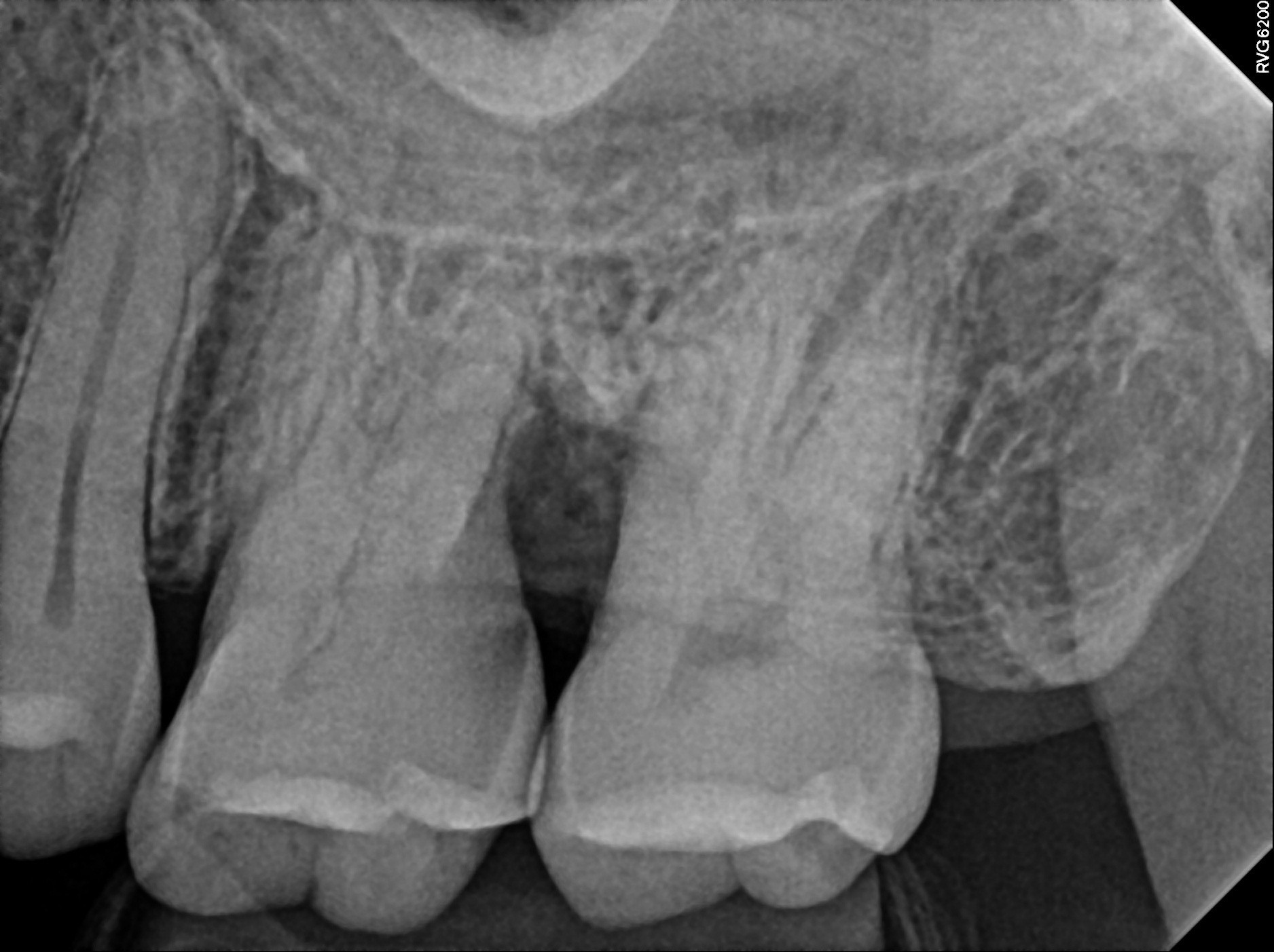

Supplement: Supplementary file 1 — Additional file 1: Test Dataset 1: Digital radiograph of upper posterior teeth. Test Dataset 2: Digital radiograph of upper posterior teeth, Test Dataset 3: Digital radiograph of upper posterior teeth, Test Dataset 4: Digital radiograph of upper posterior teeth, Test Dataset 5: Digital radiograph of upper anterior teeth, Test Dataset 6: Digital radiograph of upper anterior teeth, Test Dataset 7: Digital radiograph of lower posterior teeth, Test Dataset 8: Digital radiograph of upper posterior teeth, Test Dataset 9: Digital radiograph of lower anterior teeth, Test Dataset 10: Digital radiograph of lower anterior teeth, Test Dataset 11: Digital radiograph of lower posterior teeth, Test Dataset 12: Digital radiograph of lower anterior teeth, Test Dataset 13: Digital radiograph of upper posterior teeth, Test Dataset 14: Digital radiograph of lower teeth, Test Dataset 15: Digital radiograph of lower deciduous teeth, Test Dataset 16: Digital radiograph of lower deciduous teeth, Test Dataset 17: Digital radiograph of lower posterior teeth, Test Dataset 18: Digital radiograph of lower deciduous posterior teeth, Test Dataset 19: Digital radiograph of upper posterior teeth, Test Dataset 20: Digital radiograph of lower posterior teeth, Test Dataset 21: Digital radiograph of lower posterior teeth, Test Dataset 22: Digital radiograph of upper posterior teeth, Test Dataset 23: Digital radiograph of upper posterior teeth, Test Dataset 24: Digital radiograph of lower posterior teeth, Test Dataset 25: Digital radiograph of upper posterior teeth, Test Dataset 26: Digital radiograph of lower deciduous posterior teeth, Test Dataset 27: Digital radiograph of lower deciduous posterior teeth, Test Dataset 28: Digital radiograph of lower posterior teeth, Test Dataset 29: Digital radiograph of lower posterior teeth, Test Dataset 30: Digital radiograph of upper deciduous posterior teeth, Test Dataset 31: Digital radiograph of upper anterior teeth, Test Dataset 32: Digital radiograph of lower [file 12903_2023_3251_MOESM1_ESM.zip › Test Dataset 23.jpg]

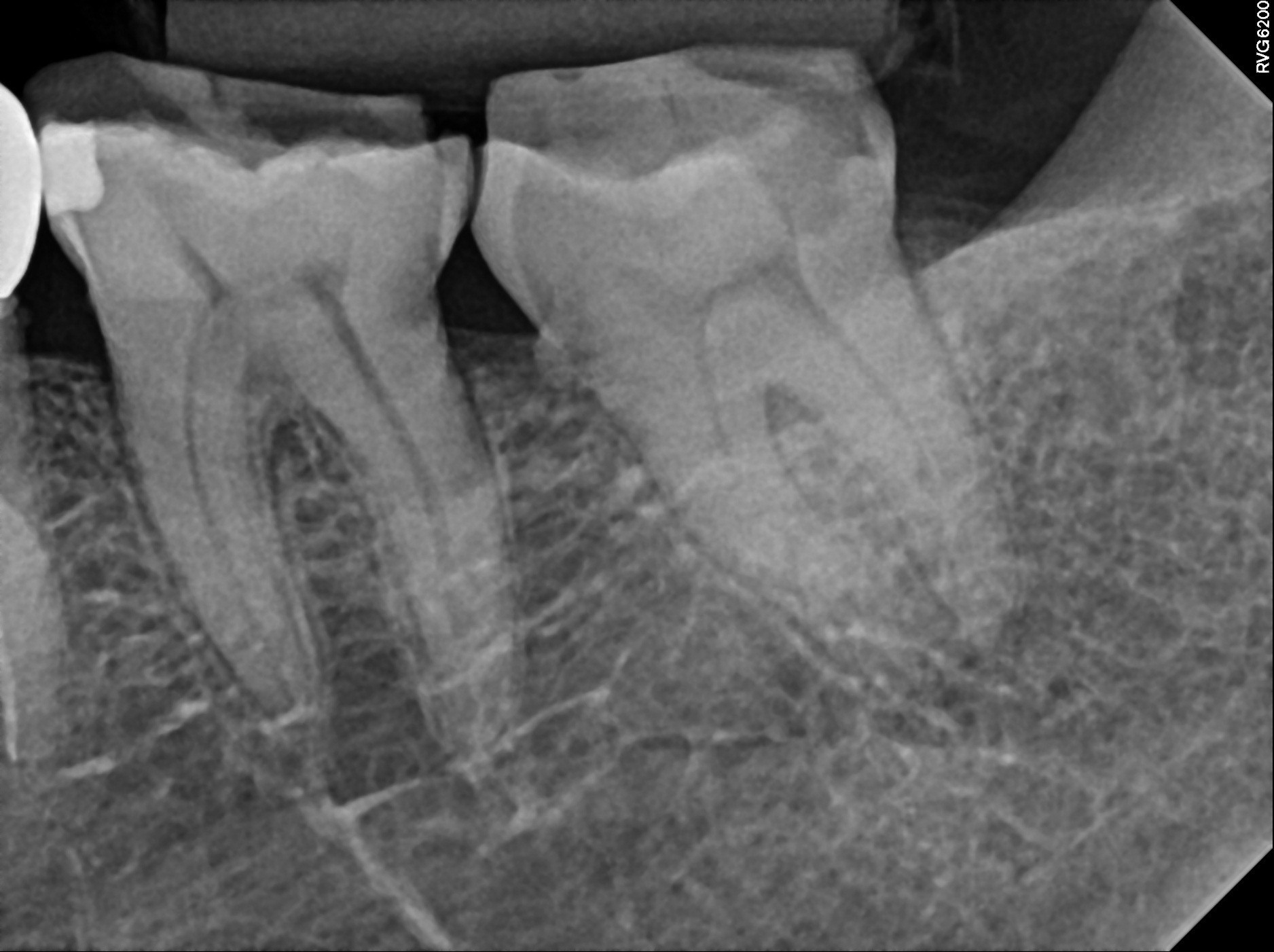

Supplement: Supplementary file 1 — Additional file 1: Test Dataset 1: Digital radiograph of upper posterior teeth. Test Dataset 2: Digital radiograph of upper posterior teeth, Test Dataset 3: Digital radiograph of upper posterior teeth, Test Dataset 4: Digital radiograph of upper posterior teeth, Test Dataset 5: Digital radiograph of upper anterior teeth, Test Dataset 6: Digital radiograph of upper anterior teeth, Test Dataset 7: Digital radiograph of lower posterior teeth, Test Dataset 8: Digital radiograph of upper posterior teeth, Test Dataset 9: Digital radiograph of lower anterior teeth, Test Dataset 10: Digital radiograph of lower anterior teeth, Test Dataset 11: Digital radiograph of lower posterior teeth, Test Dataset 12: Digital radiograph of lower anterior teeth, Test Dataset 13: Digital radiograph of upper posterior teeth, Test Dataset 14: Digital radiograph of lower teeth, Test Dataset 15: Digital radiograph of lower deciduous teeth, Test Dataset 16: Digital radiograph of lower deciduous teeth, Test Dataset 17: Digital radiograph of lower posterior teeth, Test Dataset 18: Digital radiograph of lower deciduous posterior teeth, Test Dataset 19: Digital radiograph of upper posterior teeth, Test Dataset 20: Digital radiograph of lower posterior teeth, Test Dataset 21: Digital radiograph of lower posterior teeth, Test Dataset 22: Digital radiograph of upper posterior teeth, Test Dataset 23: Digital radiograph of upper posterior teeth, Test Dataset 24: Digital radiograph of lower posterior teeth, Test Dataset 25: Digital radiograph of upper posterior teeth, Test Dataset 26: Digital radiograph of lower deciduous posterior teeth, Test Dataset 27: Digital radiograph of lower deciduous posterior teeth, Test Dataset 28: Digital radiograph of lower posterior teeth, Test Dataset 29: Digital radiograph of lower posterior teeth, Test Dataset 30: Digital radiograph of upper deciduous posterior teeth, Test Dataset 31: Digital radiograph of upper anterior teeth, Test Dataset 32: Digital radiograph of lower [file 12903_2023_3251_MOESM1_ESM.zip › Test Dataset 24.jpg]

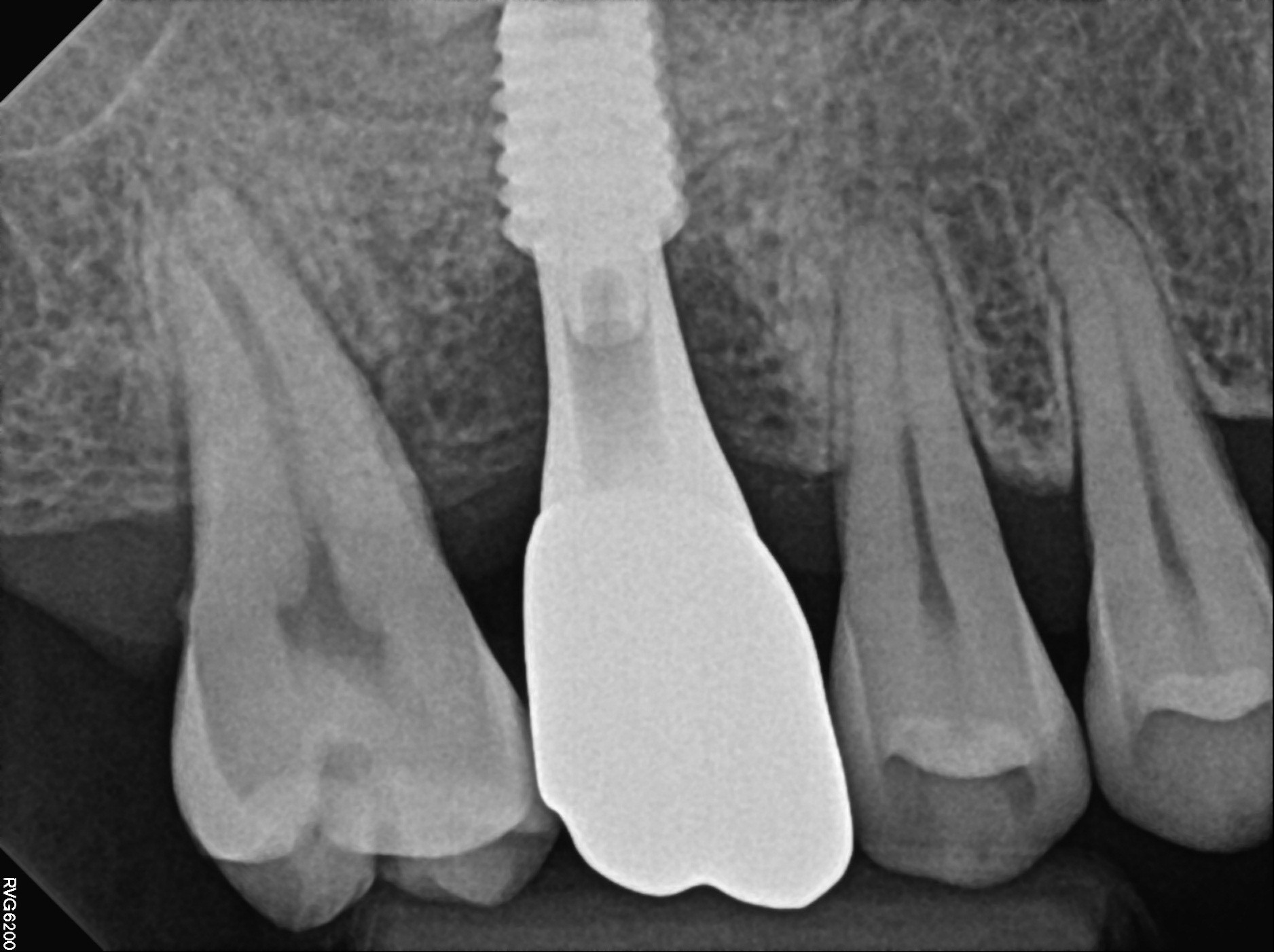

Supplement: Supplementary file 1 — Additional file 1: Test Dataset 1: Digital radiograph of upper posterior teeth. Test Dataset 2: Digital radiograph of upper posterior teeth, Test Dataset 3: Digital radiograph of upper posterior teeth, Test Dataset 4: Digital radiograph of upper posterior teeth, Test Dataset 5: Digital radiograph of upper anterior teeth, Test Dataset 6: Digital radiograph of upper anterior teeth, Test Dataset 7: Digital radiograph of lower posterior teeth, Test Dataset 8: Digital radiograph of upper posterior teeth, Test Dataset 9: Digital radiograph of lower anterior teeth, Test Dataset 10: Digital radiograph of lower anterior teeth, Test Dataset 11: Digital radiograph of lower posterior teeth, Test Dataset 12: Digital radiograph of lower anterior teeth, Test Dataset 13: Digital radiograph of upper posterior teeth, Test Dataset 14: Digital radiograph of lower teeth, Test Dataset 15: Digital radiograph of lower deciduous teeth, Test Dataset 16: Digital radiograph of lower deciduous teeth, Test Dataset 17: Digital radiograph of lower posterior teeth, Test Dataset 18: Digital radiograph of lower deciduous posterior teeth, Test Dataset 19: Digital radiograph of upper posterior teeth, Test Dataset 20: Digital radiograph of lower posterior teeth, Test Dataset 21: Digital radiograph of lower posterior teeth, Test Dataset 22: Digital radiograph of upper posterior teeth, Test Dataset 23: Digital radiograph of upper posterior teeth, Test Dataset 24: Digital radiograph of lower posterior teeth, Test Dataset 25: Digital radiograph of upper posterior teeth, Test Dataset 26: Digital radiograph of lower deciduous posterior teeth, Test Dataset 27: Digital radiograph of lower deciduous posterior teeth, Test Dataset 28: Digital radiograph of lower posterior teeth, Test Dataset 29: Digital radiograph of lower posterior teeth, Test Dataset 30: Digital radiograph of upper deciduous posterior teeth, Test Dataset 31: Digital radiograph of upper anterior teeth, Test Dataset 32: Digital radiograph of lower [file 12903_2023_3251_MOESM1_ESM.zip › Test Dataset 25.jpg]

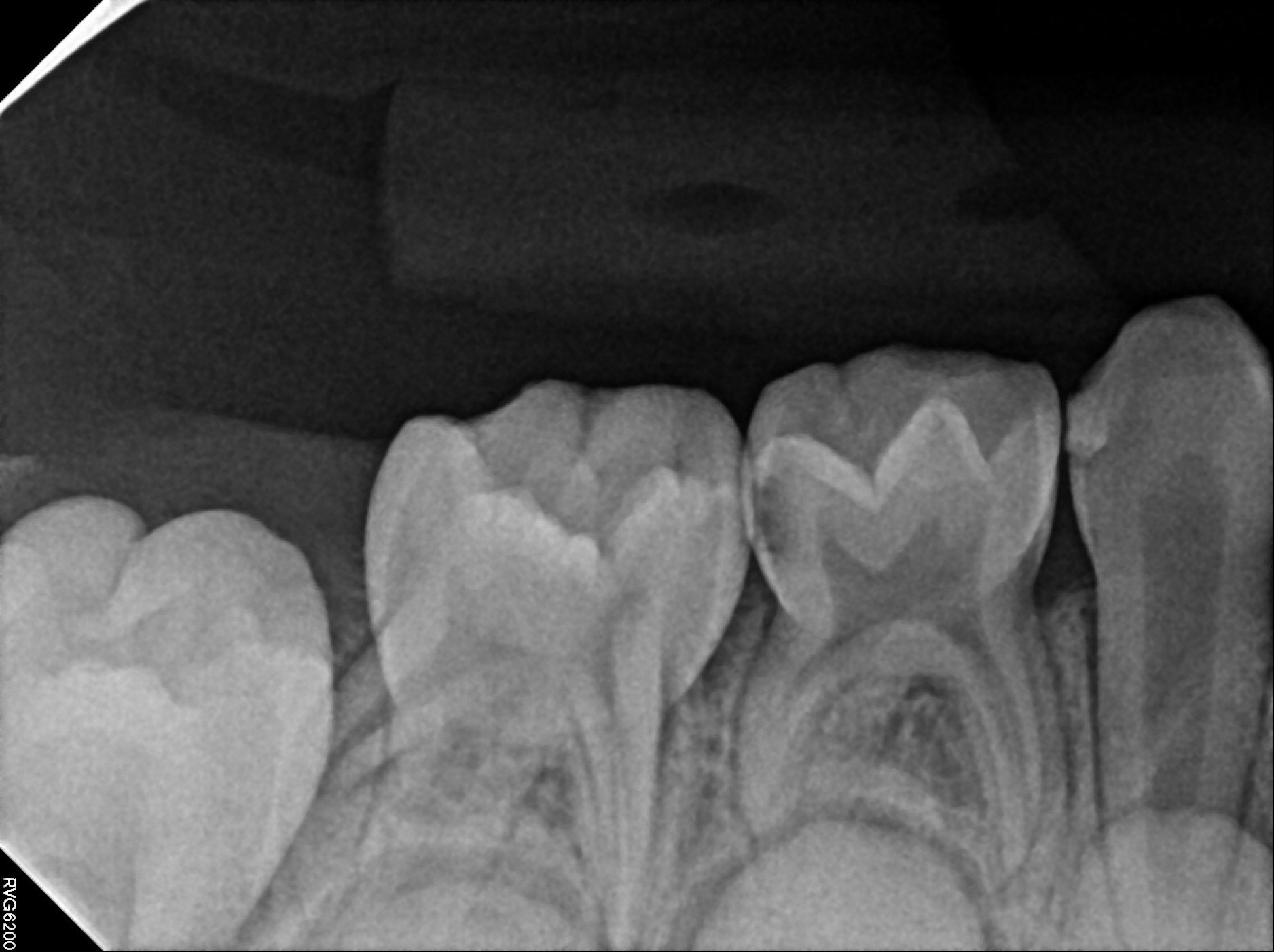

Supplement: Supplementary file 1 — Additional file 1: Test Dataset 1: Digital radiograph of upper posterior teeth. Test Dataset 2: Digital radiograph of upper posterior teeth, Test Dataset 3: Digital radiograph of upper posterior teeth, Test Dataset 4: Digital radiograph of upper posterior teeth, Test Dataset 5: Digital radiograph of upper anterior teeth, Test Dataset 6: Digital radiograph of upper anterior teeth, Test Dataset 7: Digital radiograph of lower posterior teeth, Test Dataset 8: Digital radiograph of upper posterior teeth, Test Dataset 9: Digital radiograph of lower anterior teeth, Test Dataset 10: Digital radiograph of lower anterior teeth, Test Dataset 11: Digital radiograph of lower posterior teeth, Test Dataset 12: Digital radiograph of lower anterior teeth, Test Dataset 13: Digital radiograph of upper posterior teeth, Test Dataset 14: Digital radiograph of lower teeth, Test Dataset 15: Digital radiograph of lower deciduous teeth, Test Dataset 16: Digital radiograph of lower deciduous teeth, Test Dataset 17: Digital radiograph of lower posterior teeth, Test Dataset 18: Digital radiograph of lower deciduous posterior teeth, Test Dataset 19: Digital radiograph of upper posterior teeth, Test Dataset 20: Digital radiograph of lower posterior teeth, Test Dataset 21: Digital radiograph of lower posterior teeth, Test Dataset 22: Digital radiograph of upper posterior teeth, Test Dataset 23: Digital radiograph of upper posterior teeth, Test Dataset 24: Digital radiograph of lower posterior teeth, Test Dataset 25: Digital radiograph of upper posterior teeth, Test Dataset 26: Digital radiograph of lower deciduous posterior teeth, Test Dataset 27: Digital radiograph of lower deciduous posterior teeth, Test Dataset 28: Digital radiograph of lower posterior teeth, Test Dataset 29: Digital radiograph of lower posterior teeth, Test Dataset 30: Digital radiograph of upper deciduous posterior teeth, Test Dataset 31: Digital radiograph of upper anterior teeth, Test Dataset 32: Digital radiograph of lower [file 12903_2023_3251_MOESM1_ESM.zip › Test Dataset 26.jpg]

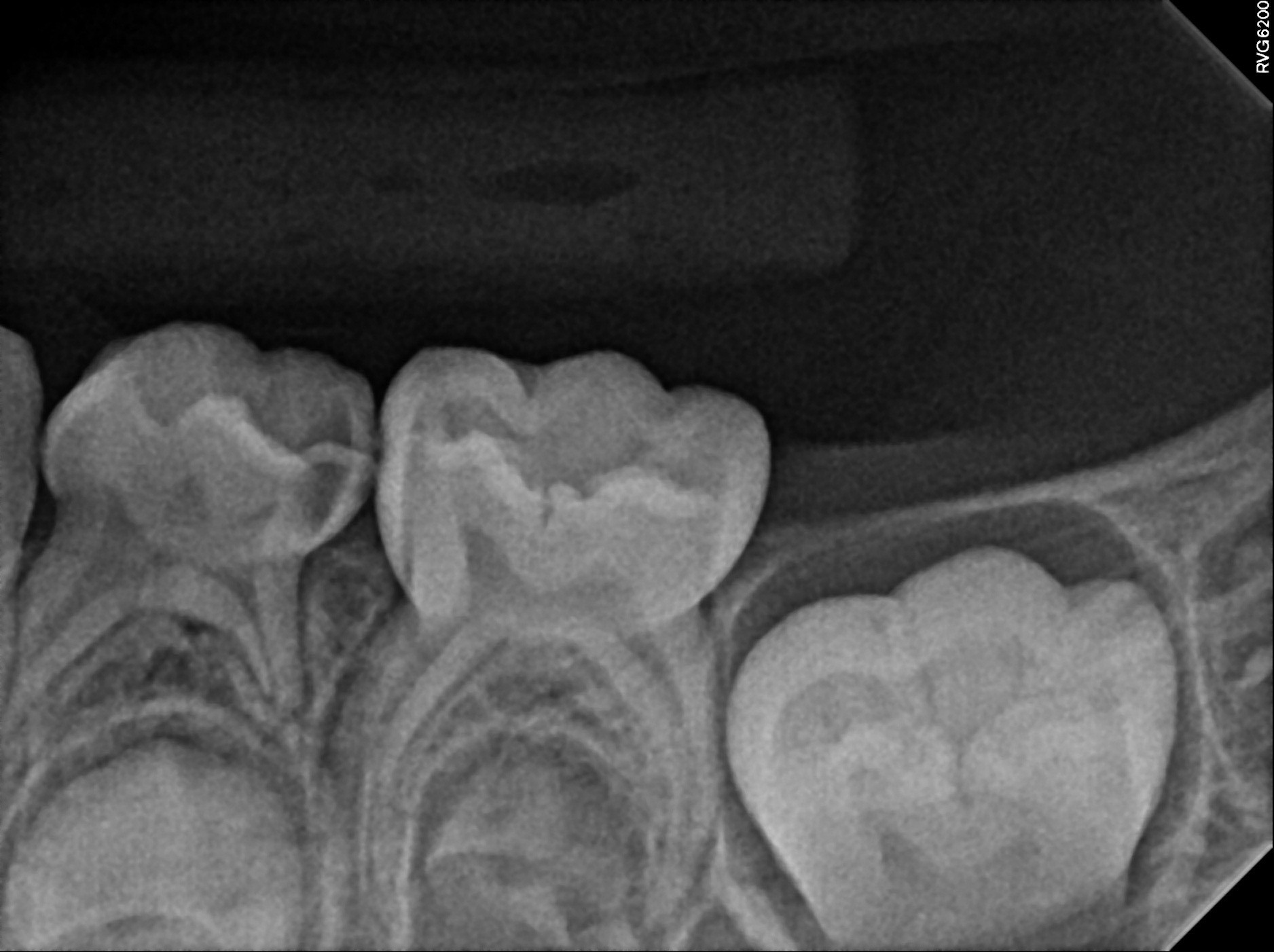

Supplement: Supplementary file 1 — Additional file 1: Test Dataset 1: Digital radiograph of upper posterior teeth. Test Dataset 2: Digital radiograph of upper posterior teeth, Test Dataset 3: Digital radiograph of upper posterior teeth, Test Dataset 4: Digital radiograph of upper posterior teeth, Test Dataset 5: Digital radiograph of upper anterior teeth, Test Dataset 6: Digital radiograph of upper anterior teeth, Test Dataset 7: Digital radiograph of lower posterior teeth, Test Dataset 8: Digital radiograph of upper posterior teeth, Test Dataset 9: Digital radiograph of lower anterior teeth, Test Dataset 10: Digital radiograph of lower anterior teeth, Test Dataset 11: Digital radiograph of lower posterior teeth, Test Dataset 12: Digital radiograph of lower anterior teeth, Test Dataset 13: Digital radiograph of upper posterior teeth, Test Dataset 14: Digital radiograph of lower teeth, Test Dataset 15: Digital radiograph of lower deciduous teeth, Test Dataset 16: Digital radiograph of lower deciduous teeth, Test Dataset 17: Digital radiograph of lower posterior teeth, Test Dataset 18: Digital radiograph of lower deciduous posterior teeth, Test Dataset 19: Digital radiograph of upper posterior teeth, Test Dataset 20: Digital radiograph of lower posterior teeth, Test Dataset 21: Digital radiograph of lower posterior teeth, Test Dataset 22: Digital radiograph of upper posterior teeth, Test Dataset 23: Digital radiograph of upper posterior teeth, Test Dataset 24: Digital radiograph of lower posterior teeth, Test Dataset 25: Digital radiograph of upper posterior teeth, Test Dataset 26: Digital radiograph of lower deciduous posterior teeth, Test Dataset 27: Digital radiograph of lower deciduous posterior teeth, Test Dataset 28: Digital radiograph of lower posterior teeth, Test Dataset 29: Digital radiograph of lower posterior teeth, Test Dataset 30: Digital radiograph of upper deciduous posterior teeth, Test Dataset 31: Digital radiograph of upper anterior teeth, Test Dataset 32: Digital radiograph of lower [file 12903_2023_3251_MOESM1_ESM.zip › Test Dataset 27.jpg]

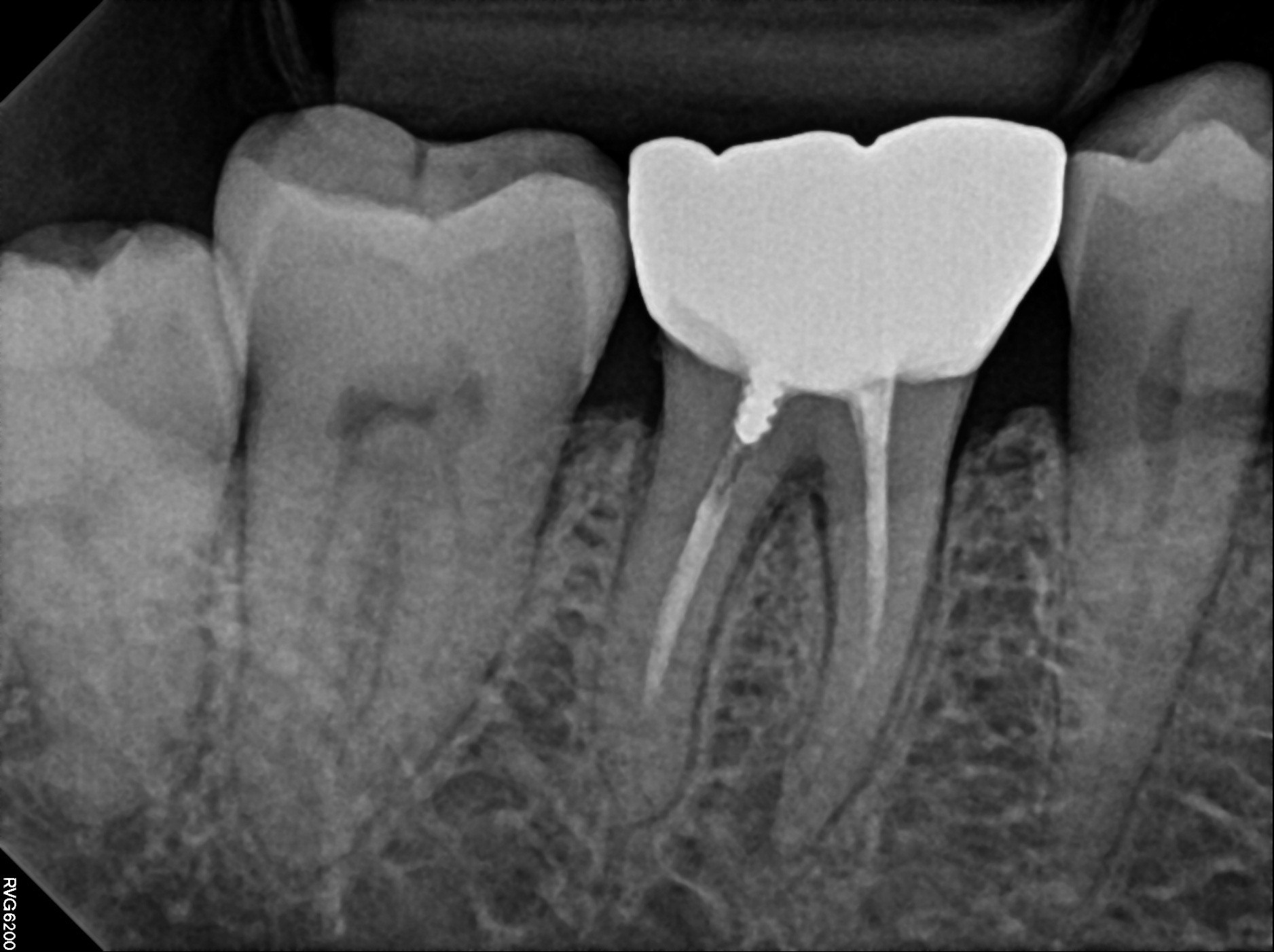

Supplement: Supplementary file 1 — Additional file 1: Test Dataset 1: Digital radiograph of upper posterior teeth. Test Dataset 2: Digital radiograph of upper posterior teeth, Test Dataset 3: Digital radiograph of upper posterior teeth, Test Dataset 4: Digital radiograph of upper posterior teeth, Test Dataset 5: Digital radiograph of upper anterior teeth, Test Dataset 6: Digital radiograph of upper anterior teeth, Test Dataset 7: Digital radiograph of lower posterior teeth, Test Dataset 8: Digital radiograph of upper posterior teeth, Test Dataset 9: Digital radiograph of lower anterior teeth, Test Dataset 10: Digital radiograph of lower anterior teeth, Test Dataset 11: Digital radiograph of lower posterior teeth, Test Dataset 12: Digital radiograph of lower anterior teeth, Test Dataset 13: Digital radiograph of upper posterior teeth, Test Dataset 14: Digital radiograph of lower teeth, Test Dataset 15: Digital radiograph of lower deciduous teeth, Test Dataset 16: Digital radiograph of lower deciduous teeth, Test Dataset 17: Digital radiograph of lower posterior teeth, Test Dataset 18: Digital radiograph of lower deciduous posterior teeth, Test Dataset 19: Digital radiograph of upper posterior teeth, Test Dataset 20: Digital radiograph of lower posterior teeth, Test Dataset 21: Digital radiograph of lower posterior teeth, Test Dataset 22: Digital radiograph of upper posterior teeth, Test Dataset 23: Digital radiograph of upper posterior teeth, Test Dataset 24: Digital radiograph of lower posterior teeth, Test Dataset 25: Digital radiograph of upper posterior teeth, Test Dataset 26: Digital radiograph of lower deciduous posterior teeth, Test Dataset 27: Digital radiograph of lower deciduous posterior teeth, Test Dataset 28: Digital radiograph of lower posterior teeth, Test Dataset 29: Digital radiograph of lower posterior teeth, Test Dataset 30: Digital radiograph of upper deciduous posterior teeth, Test Dataset 31: Digital radiograph of upper anterior teeth, Test Dataset 32: Digital radiograph of lower [file 12903_2023_3251_MOESM1_ESM.zip › Test Dataset 28.jpg]

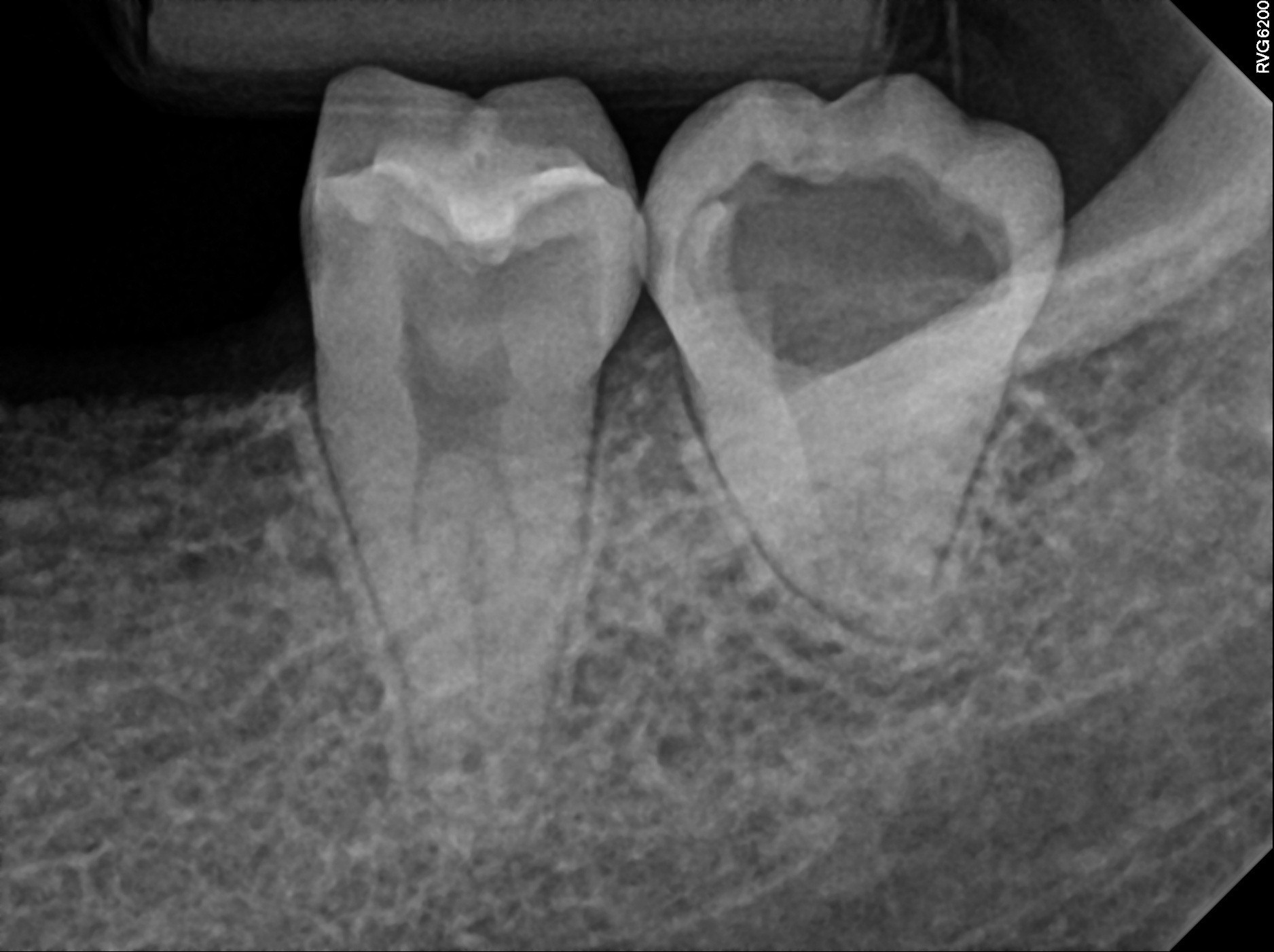

Supplement: Supplementary file 1 — Additional file 1: Test Dataset 1: Digital radiograph of upper posterior teeth. Test Dataset 2: Digital radiograph of upper posterior teeth, Test Dataset 3: Digital radiograph of upper posterior teeth, Test Dataset 4: Digital radiograph of upper posterior teeth, Test Dataset 5: Digital radiograph of upper anterior teeth, Test Dataset 6: Digital radiograph of upper anterior teeth, Test Dataset 7: Digital radiograph of lower posterior teeth, Test Dataset 8: Digital radiograph of upper posterior teeth, Test Dataset 9: Digital radiograph of lower anterior teeth, Test Dataset 10: Digital radiograph of lower anterior teeth, Test Dataset 11: Digital radiograph of lower posterior teeth, Test Dataset 12: Digital radiograph of lower anterior teeth, Test Dataset 13: Digital radiograph of upper posterior teeth, Test Dataset 14: Digital radiograph of lower teeth, Test Dataset 15: Digital radiograph of lower deciduous teeth, Test Dataset 16: Digital radiograph of lower deciduous teeth, Test Dataset 17: Digital radiograph of lower posterior teeth, Test Dataset 18: Digital radiograph of lower deciduous posterior teeth, Test Dataset 19: Digital radiograph of upper posterior teeth, Test Dataset 20: Digital radiograph of lower posterior teeth, Test Dataset 21: Digital radiograph of lower posterior teeth, Test Dataset 22: Digital radiograph of upper posterior teeth, Test Dataset 23: Digital radiograph of upper posterior teeth, Test Dataset 24: Digital radiograph of lower posterior teeth, Test Dataset 25: Digital radiograph of upper posterior teeth, Test Dataset 26: Digital radiograph of lower deciduous posterior teeth, Test Dataset 27: Digital radiograph of lower deciduous posterior teeth, Test Dataset 28: Digital radiograph of lower posterior teeth, Test Dataset 29: Digital radiograph of lower posterior teeth, Test Dataset 30: Digital radiograph of upper deciduous posterior teeth, Test Dataset 31: Digital radiograph of upper anterior teeth, Test Dataset 32: Digital radiograph of lower [file 12903_2023_3251_MOESM1_ESM.zip › Test Dataset 29.jpg]

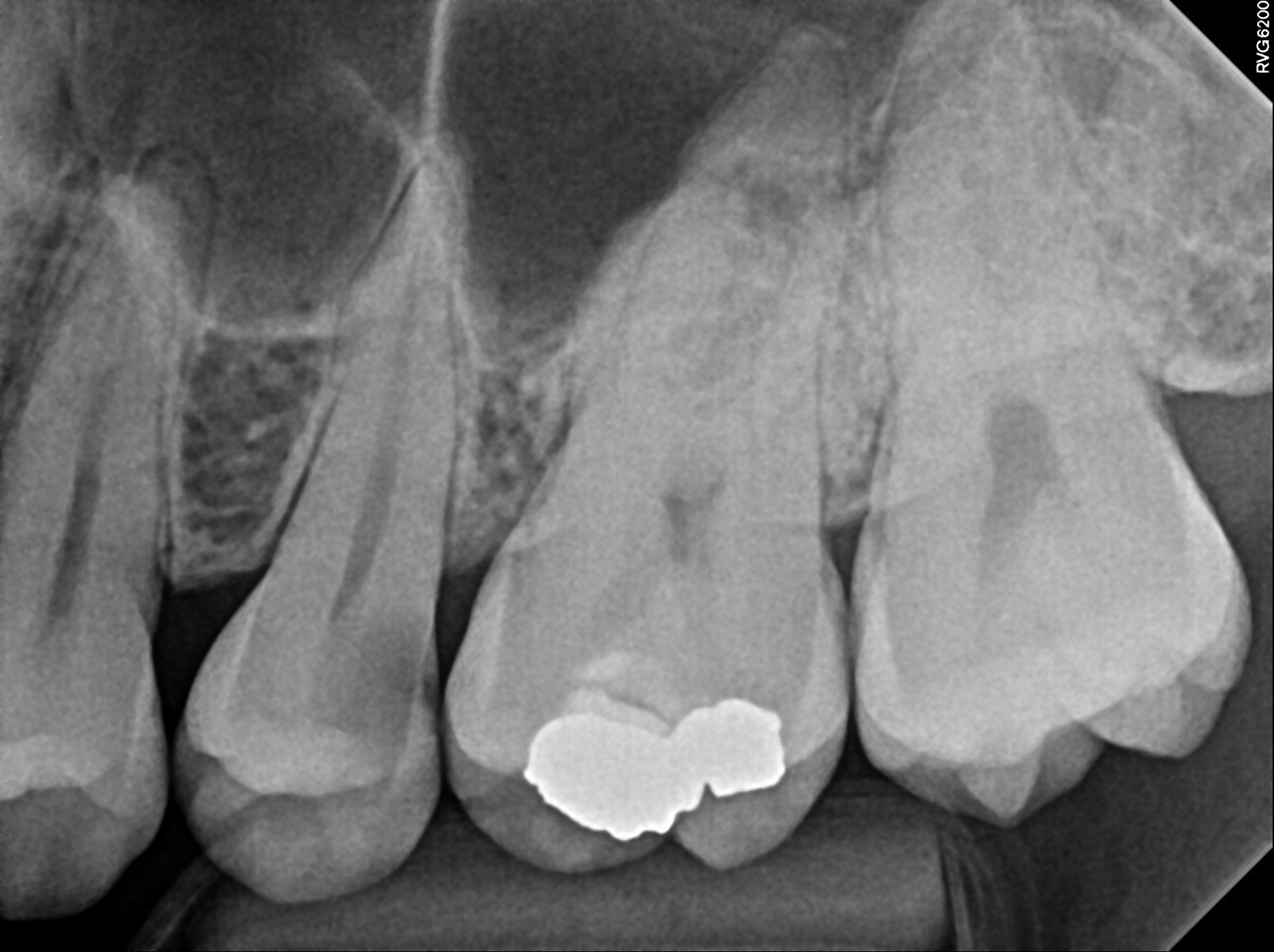

Supplement: Supplementary file 1 — Additional file 1: Test Dataset 1: Digital radiograph of upper posterior teeth. Test Dataset 2: Digital radiograph of upper posterior teeth, Test Dataset 3: Digital radiograph of upper posterior teeth, Test Dataset 4: Digital radiograph of upper posterior teeth, Test Dataset 5: Digital radiograph of upper anterior teeth, Test Dataset 6: Digital radiograph of upper anterior teeth, Test Dataset 7: Digital radiograph of lower posterior teeth, Test Dataset 8: Digital radiograph of upper posterior teeth, Test Dataset 9: Digital radiograph of lower anterior teeth, Test Dataset 10: Digital radiograph of lower anterior teeth, Test Dataset 11: Digital radiograph of lower posterior teeth, Test Dataset 12: Digital radiograph of lower anterior teeth, Test Dataset 13: Digital radiograph of upper posterior teeth, Test Dataset 14: Digital radiograph of lower teeth, Test Dataset 15: Digital radiograph of lower deciduous teeth, Test Dataset 16: Digital radiograph of lower deciduous teeth, Test Dataset 17: Digital radiograph of lower posterior teeth, Test Dataset 18: Digital radiograph of lower deciduous posterior teeth, Test Dataset 19: Digital radiograph of upper posterior teeth, Test Dataset 20: Digital radiograph of lower posterior teeth, Test Dataset 21: Digital radiograph of lower posterior teeth, Test Dataset 22: Digital radiograph of upper posterior teeth, Test Dataset 23: Digital radiograph of upper posterior teeth, Test Dataset 24: Digital radiograph of lower posterior teeth, Test Dataset 25: Digital radiograph of upper posterior teeth, Test Dataset 26: Digital radiograph of lower deciduous posterior teeth, Test Dataset 27: Digital radiograph of lower deciduous posterior teeth, Test Dataset 28: Digital radiograph of lower posterior teeth, Test Dataset 29: Digital radiograph of lower posterior teeth, Test Dataset 30: Digital radiograph of upper deciduous posterior teeth, Test Dataset 31: Digital radiograph of upper anterior teeth, Test Dataset 32: Digital radiograph of lower [file 12903_2023_3251_MOESM1_ESM.zip › Test Dataset 3.jpg]

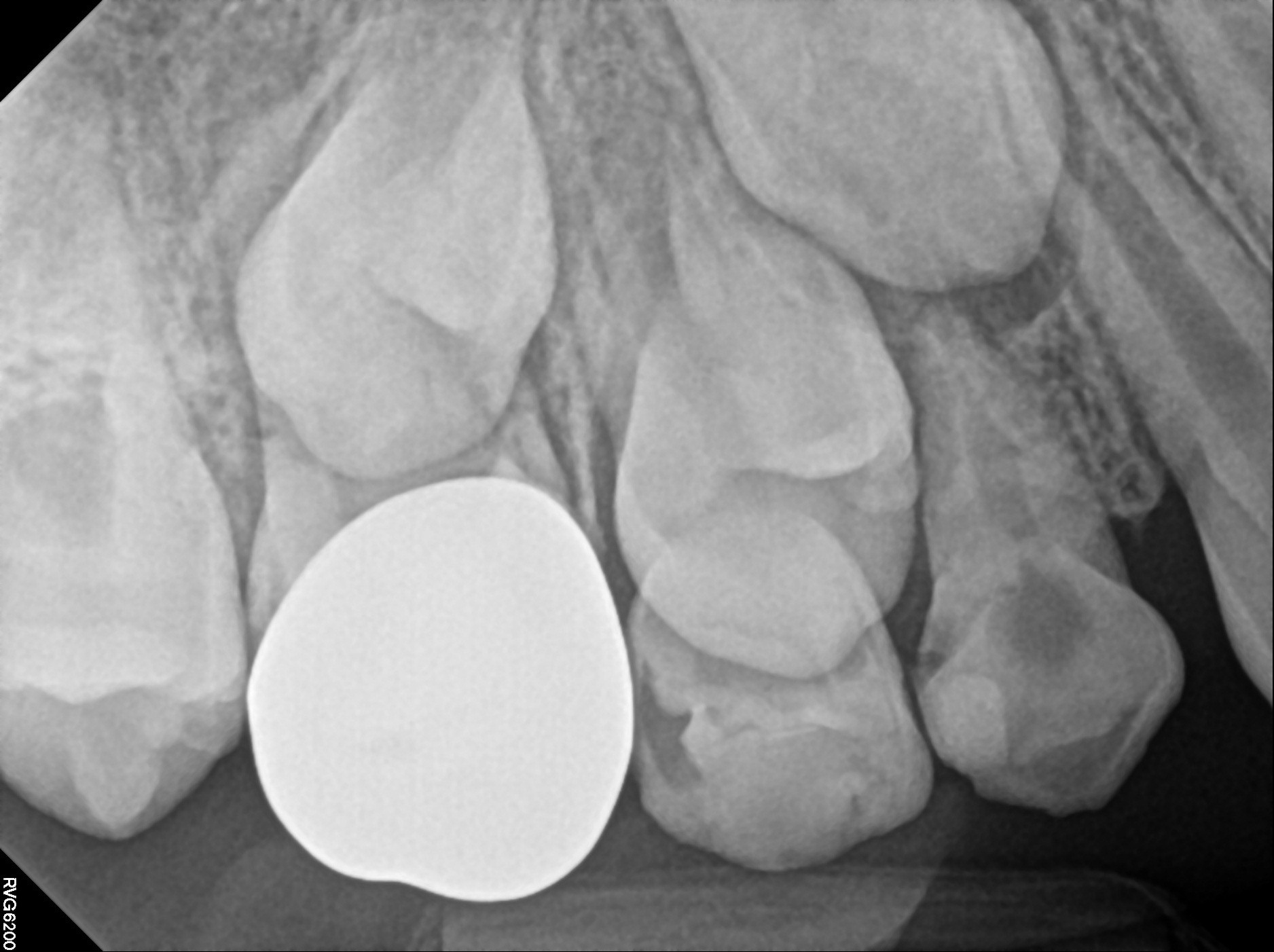

Supplement: Supplementary file 1 — Additional file 1: Test Dataset 1: Digital radiograph of upper posterior teeth. Test Dataset 2: Digital radiograph of upper posterior teeth, Test Dataset 3: Digital radiograph of upper posterior teeth, Test Dataset 4: Digital radiograph of upper posterior teeth, Test Dataset 5: Digital radiograph of upper anterior teeth, Test Dataset 6: Digital radiograph of upper anterior teeth, Test Dataset 7: Digital radiograph of lower posterior teeth, Test Dataset 8: Digital radiograph of upper posterior teeth, Test Dataset 9: Digital radiograph of lower anterior teeth, Test Dataset 10: Digital radiograph of lower anterior teeth, Test Dataset 11: Digital radiograph of lower posterior teeth, Test Dataset 12: Digital radiograph of lower anterior teeth, Test Dataset 13: Digital radiograph of upper posterior teeth, Test Dataset 14: Digital radiograph of lower teeth, Test Dataset 15: Digital radiograph of lower deciduous teeth, Test Dataset 16: Digital radiograph of lower deciduous teeth, Test Dataset 17: Digital radiograph of lower posterior teeth, Test Dataset 18: Digital radiograph of lower deciduous posterior teeth, Test Dataset 19: Digital radiograph of upper posterior teeth, Test Dataset 20: Digital radiograph of lower posterior teeth, Test Dataset 21: Digital radiograph of lower posterior teeth, Test Dataset 22: Digital radiograph of upper posterior teeth, Test Dataset 23: Digital radiograph of upper posterior teeth, Test Dataset 24: Digital radiograph of lower posterior teeth, Test Dataset 25: Digital radiograph of upper posterior teeth, Test Dataset 26: Digital radiograph of lower deciduous posterior teeth, Test Dataset 27: Digital radiograph of lower deciduous posterior teeth, Test Dataset 28: Digital radiograph of lower posterior teeth, Test Dataset 29: Digital radiograph of lower posterior teeth, Test Dataset 30: Digital radiograph of upper deciduous posterior teeth, Test Dataset 31: Digital radiograph of upper anterior teeth, Test Dataset 32: Digital radiograph of lower [file 12903_2023_3251_MOESM1_ESM.zip › Test Dataset 30.jpg]

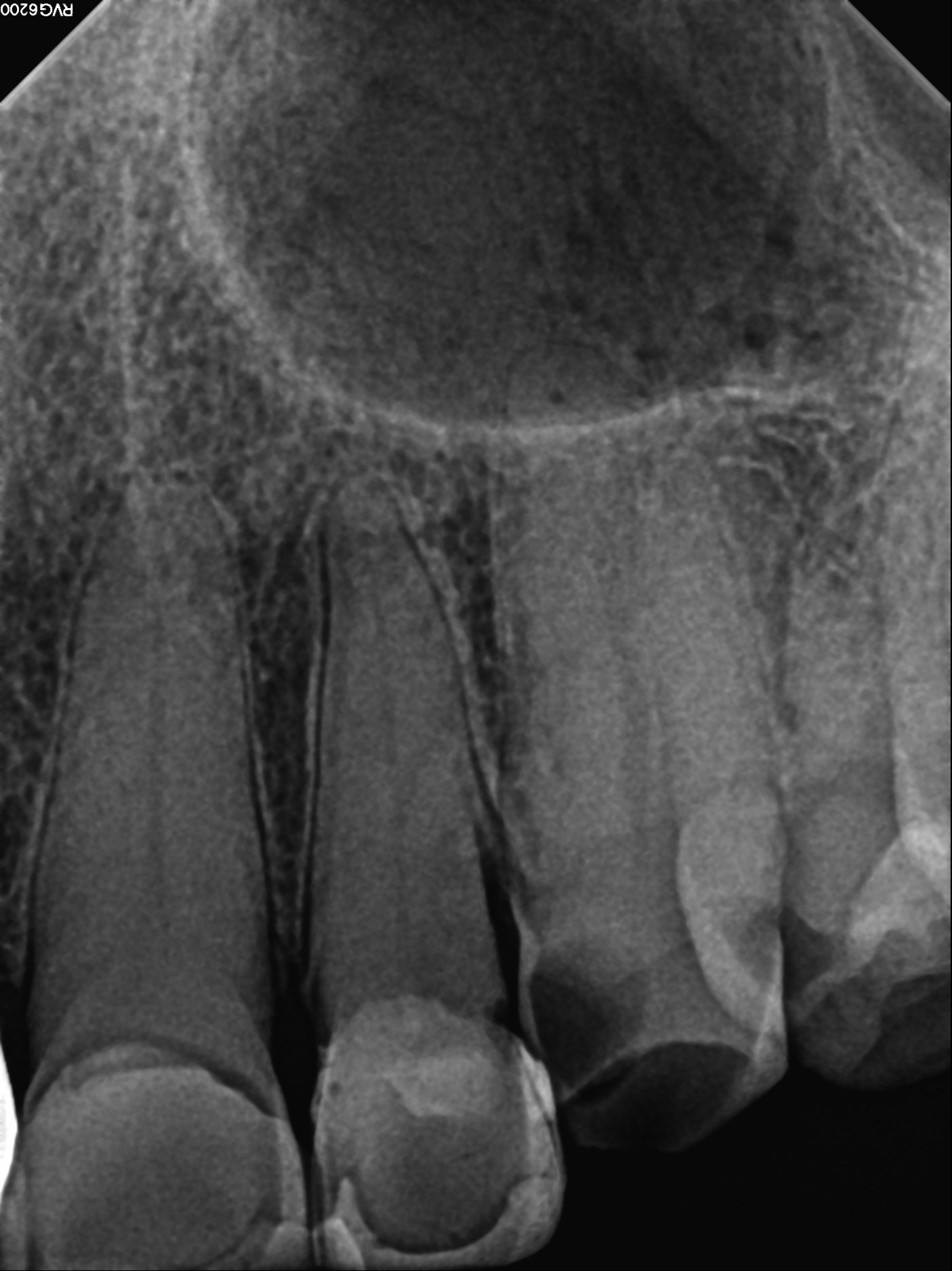

Supplement: Supplementary file 1 — Additional file 1: Test Dataset 1: Digital radiograph of upper posterior teeth. Test Dataset 2: Digital radiograph of upper posterior teeth, Test Dataset 3: Digital radiograph of upper posterior teeth, Test Dataset 4: Digital radiograph of upper posterior teeth, Test Dataset 5: Digital radiograph of upper anterior teeth, Test Dataset 6: Digital radiograph of upper anterior teeth, Test Dataset 7: Digital radiograph of lower posterior teeth, Test Dataset 8: Digital radiograph of upper posterior teeth, Test Dataset 9: Digital radiograph of lower anterior teeth, Test Dataset 10: Digital radiograph of lower anterior teeth, Test Dataset 11: Digital radiograph of lower posterior teeth, Test Dataset 12: Digital radiograph of lower anterior teeth, Test Dataset 13: Digital radiograph of upper posterior teeth, Test Dataset 14: Digital radiograph of lower teeth, Test Dataset 15: Digital radiograph of lower deciduous teeth, Test Dataset 16: Digital radiograph of lower deciduous teeth, Test Dataset 17: Digital radiograph of lower posterior teeth, Test Dataset 18: Digital radiograph of lower deciduous posterior teeth, Test Dataset 19: Digital radiograph of upper posterior teeth, Test Dataset 20: Digital radiograph of lower posterior teeth, Test Dataset 21: Digital radiograph of lower posterior teeth, Test Dataset 22: Digital radiograph of upper posterior teeth, Test Dataset 23: Digital radiograph of upper posterior teeth, Test Dataset 24: Digital radiograph of lower posterior teeth, Test Dataset 25: Digital radiograph of upper posterior teeth, Test Dataset 26: Digital radiograph of lower deciduous posterior teeth, Test Dataset 27: Digital radiograph of lower deciduous posterior teeth, Test Dataset 28: Digital radiograph of lower posterior teeth, Test Dataset 29: Digital radiograph of lower posterior teeth, Test Dataset 30: Digital radiograph of upper deciduous posterior teeth, Test Dataset 31: Digital radiograph of upper anterior teeth, Test Dataset 32: Digital radiograph of lower [file 12903_2023_3251_MOESM1_ESM.zip › Test Dataset 31.jpg]

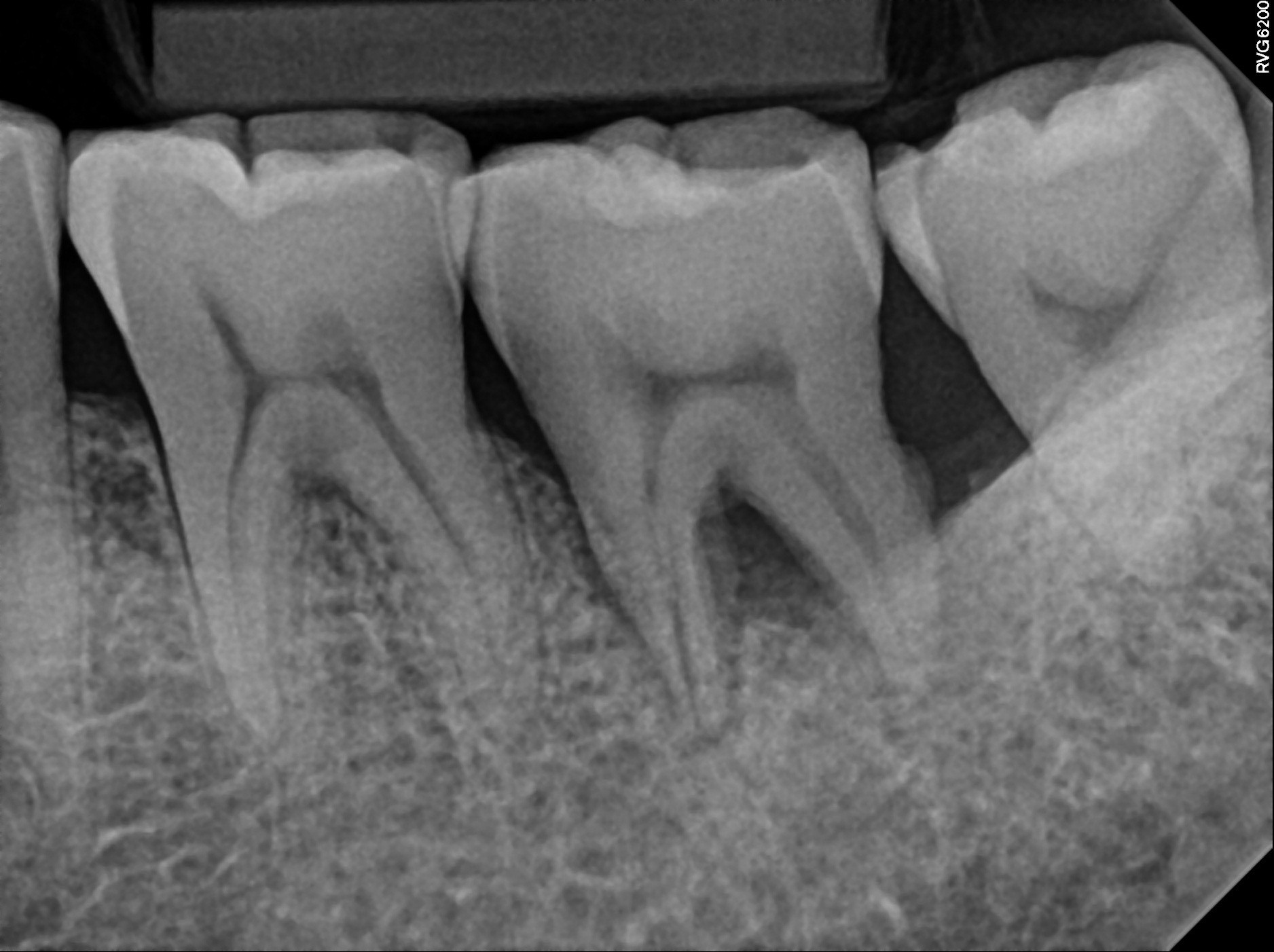

Supplement: Supplementary file 1 — Additional file 1: Test Dataset 1: Digital radiograph of upper posterior teeth. Test Dataset 2: Digital radiograph of upper posterior teeth, Test Dataset 3: Digital radiograph of upper posterior teeth, Test Dataset 4: Digital radiograph of upper posterior teeth, Test Dataset 5: Digital radiograph of upper anterior teeth, Test Dataset 6: Digital radiograph of upper anterior teeth, Test Dataset 7: Digital radiograph of lower posterior teeth, Test Dataset 8: Digital radiograph of upper posterior teeth, Test Dataset 9: Digital radiograph of lower anterior teeth, Test Dataset 10: Digital radiograph of lower anterior teeth, Test Dataset 11: Digital radiograph of lower posterior teeth, Test Dataset 12: Digital radiograph of lower anterior teeth, Test Dataset 13: Digital radiograph of upper posterior teeth, Test Dataset 14: Digital radiograph of lower teeth, Test Dataset 15: Digital radiograph of lower deciduous teeth, Test Dataset 16: Digital radiograph of lower deciduous teeth, Test Dataset 17: Digital radiograph of lower posterior teeth, Test Dataset 18: Digital radiograph of lower deciduous posterior teeth, Test Dataset 19: Digital radiograph of upper posterior teeth, Test Dataset 20: Digital radiograph of lower posterior teeth, Test Dataset 21: Digital radiograph of lower posterior teeth, Test Dataset 22: Digital radiograph of upper posterior teeth, Test Dataset 23: Digital radiograph of upper posterior teeth, Test Dataset 24: Digital radiograph of lower posterior teeth, Test Dataset 25: Digital radiograph of upper posterior teeth, Test Dataset 26: Digital radiograph of lower deciduous posterior teeth, Test Dataset 27: Digital radiograph of lower deciduous posterior teeth, Test Dataset 28: Digital radiograph of lower posterior teeth, Test Dataset 29: Digital radiograph of lower posterior teeth, Test Dataset 30: Digital radiograph of upper deciduous posterior teeth, Test Dataset 31: Digital radiograph of upper anterior teeth, Test Dataset 32: Digital radiograph of lower [file 12903_2023_3251_MOESM1_ESM.zip › Test Dataset 32.jpg]

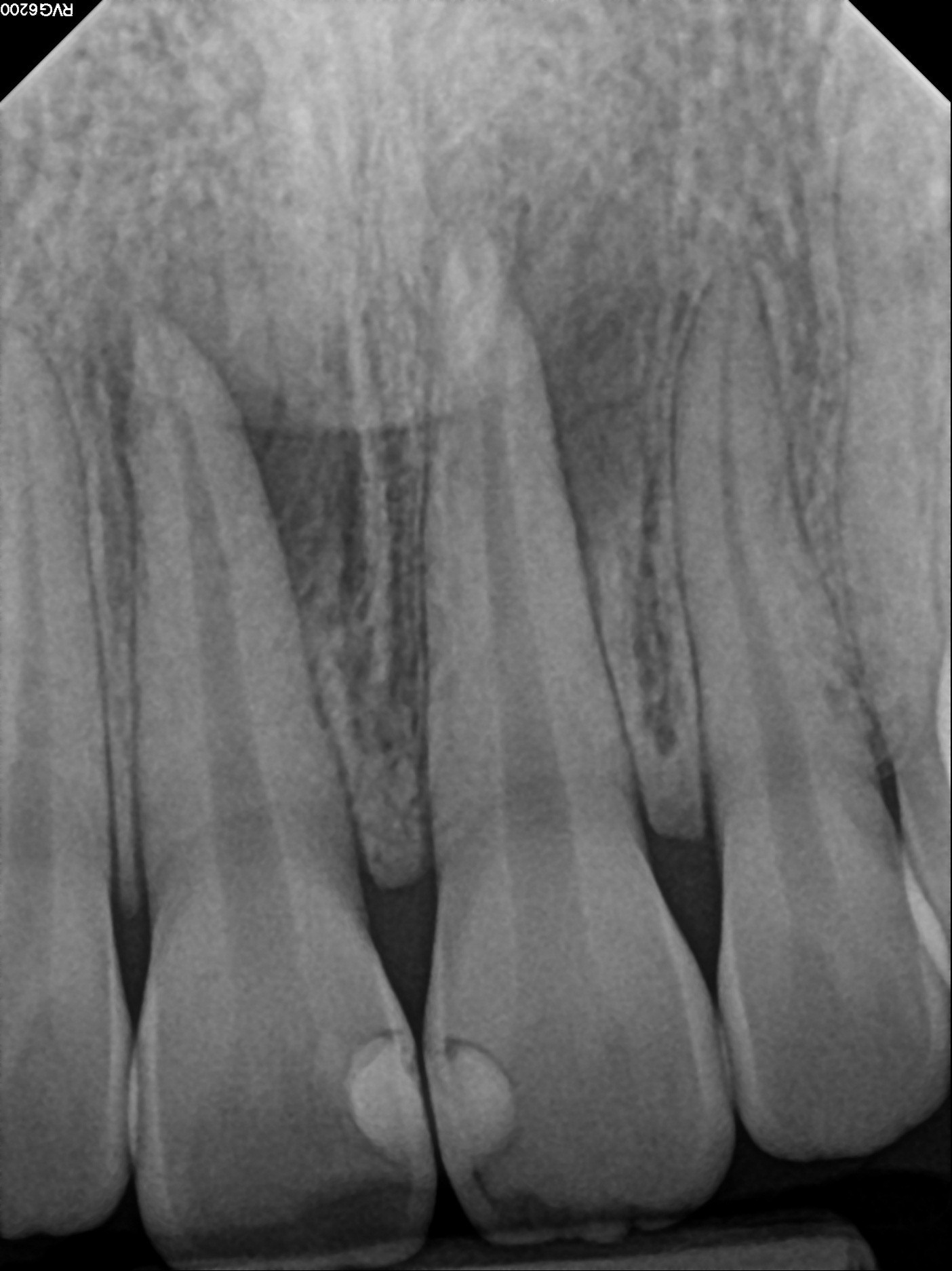

Supplement: Supplementary file 1 — Additional file 1: Test Dataset 1: Digital radiograph of upper posterior teeth. Test Dataset 2: Digital radiograph of upper posterior teeth, Test Dataset 3: Digital radiograph of upper posterior teeth, Test Dataset 4: Digital radiograph of upper posterior teeth, Test Dataset 5: Digital radiograph of upper anterior teeth, Test Dataset 6: Digital radiograph of upper anterior teeth, Test Dataset 7: Digital radiograph of lower posterior teeth, Test Dataset 8: Digital radiograph of upper posterior teeth, Test Dataset 9: Digital radiograph of lower anterior teeth, Test Dataset 10: Digital radiograph of lower anterior teeth, Test Dataset 11: Digital radiograph of lower posterior teeth, Test Dataset 12: Digital radiograph of lower anterior teeth, Test Dataset 13: Digital radiograph of upper posterior teeth, Test Dataset 14: Digital radiograph of lower teeth, Test Dataset 15: Digital radiograph of lower deciduous teeth, Test Dataset 16: Digital radiograph of lower deciduous teeth, Test Dataset 17: Digital radiograph of lower posterior teeth, Test Dataset 18: Digital radiograph of lower deciduous posterior teeth, Test Dataset 19: Digital radiograph of upper posterior teeth, Test Dataset 20: Digital radiograph of lower posterior teeth, Test Dataset 21: Digital radiograph of lower posterior teeth, Test Dataset 22: Digital radiograph of upper posterior teeth, Test Dataset 23: Digital radiograph of upper posterior teeth, Test Dataset 24: Digital radiograph of lower posterior teeth, Test Dataset 25: Digital radiograph of upper posterior teeth, Test Dataset 26: Digital radiograph of lower deciduous posterior teeth, Test Dataset 27: Digital radiograph of lower deciduous posterior teeth, Test Dataset 28: Digital radiograph of lower posterior teeth, Test Dataset 29: Digital radiograph of lower posterior teeth, Test Dataset 30: Digital radiograph of upper deciduous posterior teeth, Test Dataset 31: Digital radiograph of upper anterior teeth, Test Dataset 32: Digital radiograph of lower [file 12903_2023_3251_MOESM1_ESM.zip › Test Dataset 33.jpg]

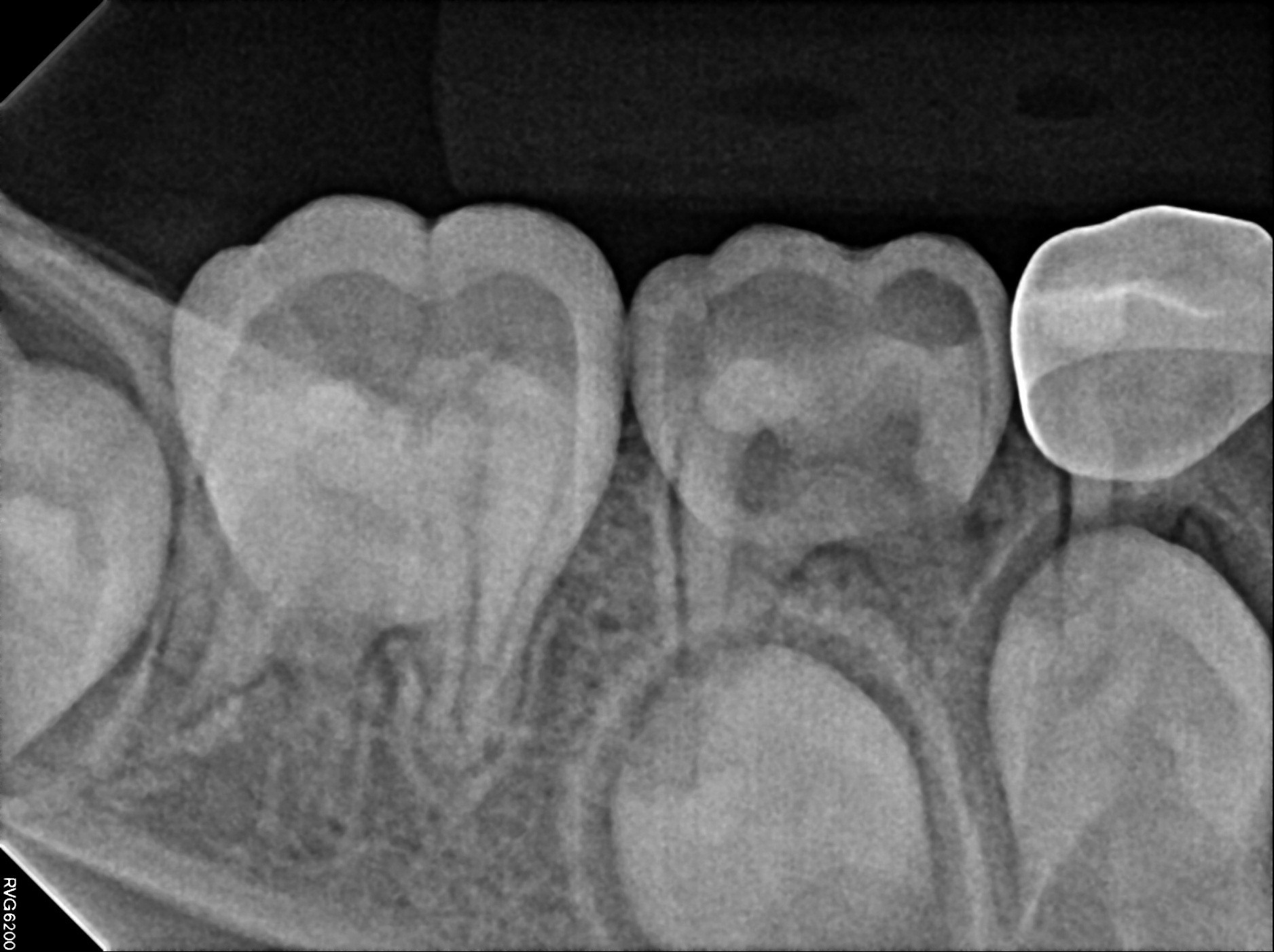

Supplement: Supplementary file 1 — Additional file 1: Test Dataset 1: Digital radiograph of upper posterior teeth. Test Dataset 2: Digital radiograph of upper posterior teeth, Test Dataset 3: Digital radiograph of upper posterior teeth, Test Dataset 4: Digital radiograph of upper posterior teeth, Test Dataset 5: Digital radiograph of upper anterior teeth, Test Dataset 6: Digital radiograph of upper anterior teeth, Test Dataset 7: Digital radiograph of lower posterior teeth, Test Dataset 8: Digital radiograph of upper posterior teeth, Test Dataset 9: Digital radiograph of lower anterior teeth, Test Dataset 10: Digital radiograph of lower anterior teeth, Test Dataset 11: Digital radiograph of lower posterior teeth, Test Dataset 12: Digital radiograph of lower anterior teeth, Test Dataset 13: Digital radiograph of upper posterior teeth, Test Dataset 14: Digital radiograph of lower teeth, Test Dataset 15: Digital radiograph of lower deciduous teeth, Test Dataset 16: Digital radiograph of lower deciduous teeth, Test Dataset 17: Digital radiograph of lower posterior teeth, Test Dataset 18: Digital radiograph of lower deciduous posterior teeth, Test Dataset 19: Digital radiograph of upper posterior teeth, Test Dataset 20: Digital radiograph of lower posterior teeth, Test Dataset 21: Digital radiograph of lower posterior teeth, Test Dataset 22: Digital radiograph of upper posterior teeth, Test Dataset 23: Digital radiograph of upper posterior teeth, Test Dataset 24: Digital radiograph of lower posterior teeth, Test Dataset 25: Digital radiograph of upper posterior teeth, Test Dataset 26: Digital radiograph of lower deciduous posterior teeth, Test Dataset 27: Digital radiograph of lower deciduous posterior teeth, Test Dataset 28: Digital radiograph of lower posterior teeth, Test Dataset 29: Digital radiograph of lower posterior teeth, Test Dataset 30: Digital radiograph of upper deciduous posterior teeth, Test Dataset 31: Digital radiograph of upper anterior teeth, Test Dataset 32: Digital radiograph of lower [file 12903_2023_3251_MOESM1_ESM.zip › Test Dataset 34.jpg]

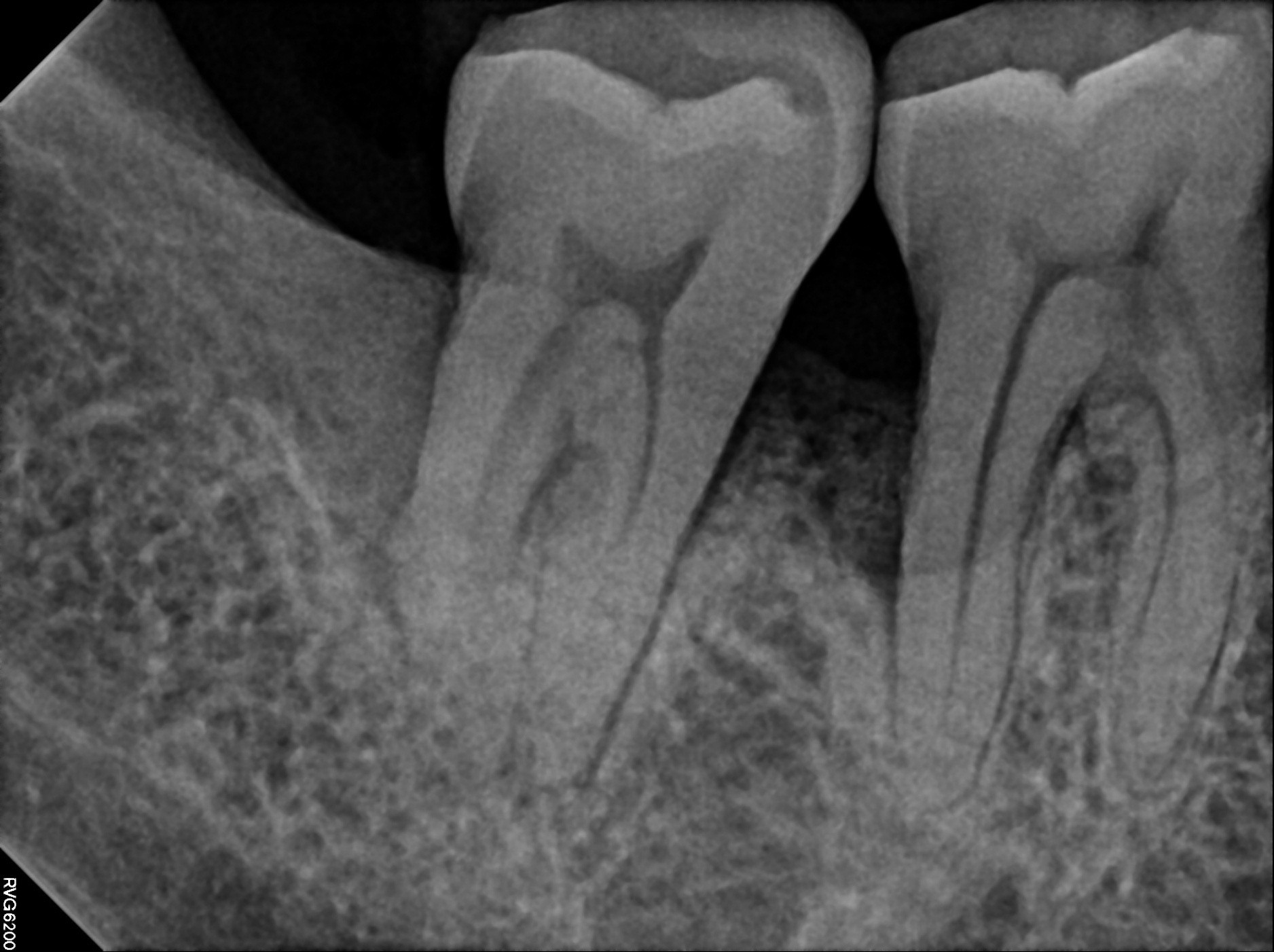

Supplement: Supplementary file 1 — Additional file 1: Test Dataset 1: Digital radiograph of upper posterior teeth. Test Dataset 2: Digital radiograph of upper posterior teeth, Test Dataset 3: Digital radiograph of upper posterior teeth, Test Dataset 4: Digital radiograph of upper posterior teeth, Test Dataset 5: Digital radiograph of upper anterior teeth, Test Dataset 6: Digital radiograph of upper anterior teeth, Test Dataset 7: Digital radiograph of lower posterior teeth, Test Dataset 8: Digital radiograph of upper posterior teeth, Test Dataset 9: Digital radiograph of lower anterior teeth, Test Dataset 10: Digital radiograph of lower anterior teeth, Test Dataset 11: Digital radiograph of lower posterior teeth, Test Dataset 12: Digital radiograph of lower anterior teeth, Test Dataset 13: Digital radiograph of upper posterior teeth, Test Dataset 14: Digital radiograph of lower teeth, Test Dataset 15: Digital radiograph of lower deciduous teeth, Test Dataset 16: Digital radiograph of lower deciduous teeth, Test Dataset 17: Digital radiograph of lower posterior teeth, Test Dataset 18: Digital radiograph of lower deciduous posterior teeth, Test Dataset 19: Digital radiograph of upper posterior teeth, Test Dataset 20: Digital radiograph of lower posterior teeth, Test Dataset 21: Digital radiograph of lower posterior teeth, Test Dataset 22: Digital radiograph of upper posterior teeth, Test Dataset 23: Digital radiograph of upper posterior teeth, Test Dataset 24: Digital radiograph of lower posterior teeth, Test Dataset 25: Digital radiograph of upper posterior teeth, Test Dataset 26: Digital radiograph of lower deciduous posterior teeth, Test Dataset 27: Digital radiograph of lower deciduous posterior teeth, Test Dataset 28: Digital radiograph of lower posterior teeth, Test Dataset 29: Digital radiograph of lower posterior teeth, Test Dataset 30: Digital radiograph of upper deciduous posterior teeth, Test Dataset 31: Digital radiograph of upper anterior teeth, Test Dataset 32: Digital radiograph of lower [file 12903_2023_3251_MOESM1_ESM.zip › Test Dataset 35.jpg]

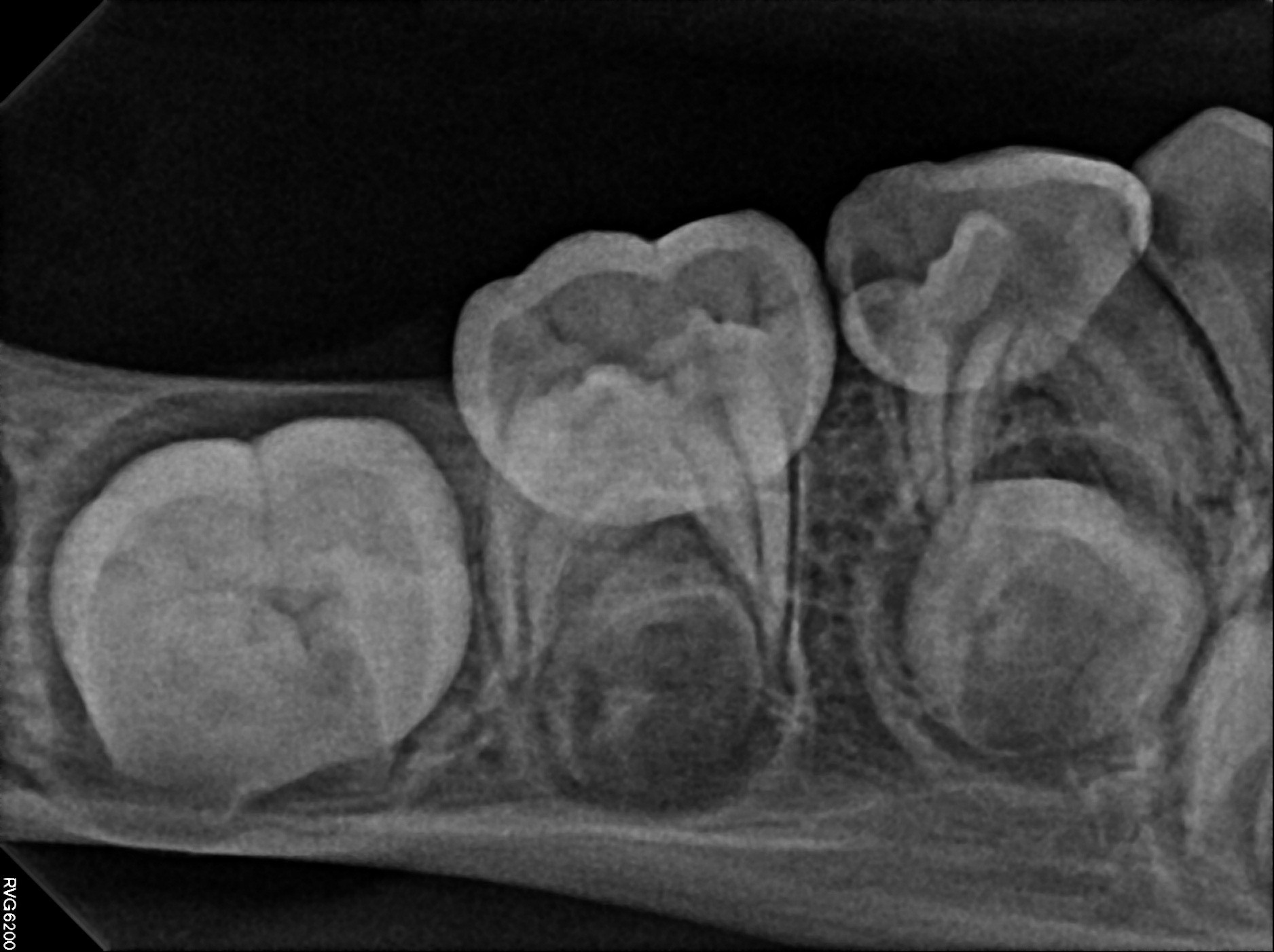

Supplement: Supplementary file 1 — Additional file 1: Test Dataset 1: Digital radiograph of upper posterior teeth. Test Dataset 2: Digital radiograph of upper posterior teeth, Test Dataset 3: Digital radiograph of upper posterior teeth, Test Dataset 4: Digital radiograph of upper posterior teeth, Test Dataset 5: Digital radiograph of upper anterior teeth, Test Dataset 6: Digital radiograph of upper anterior teeth, Test Dataset 7: Digital radiograph of lower posterior teeth, Test Dataset 8: Digital radiograph of upper posterior teeth, Test Dataset 9: Digital radiograph of lower anterior teeth, Test Dataset 10: Digital radiograph of lower anterior teeth, Test Dataset 11: Digital radiograph of lower posterior teeth, Test Dataset 12: Digital radiograph of lower anterior teeth, Test Dataset 13: Digital radiograph of upper posterior teeth, Test Dataset 14: Digital radiograph of lower teeth, Test Dataset 15: Digital radiograph of lower deciduous teeth, Test Dataset 16: Digital radiograph of lower deciduous teeth, Test Dataset 17: Digital radiograph of lower posterior teeth, Test Dataset 18: Digital radiograph of lower deciduous posterior teeth, Test Dataset 19: Digital radiograph of upper posterior teeth, Test Dataset 20: Digital radiograph of lower posterior teeth, Test Dataset 21: Digital radiograph of lower posterior teeth, Test Dataset 22: Digital radiograph of upper posterior teeth, Test Dataset 23: Digital radiograph of upper posterior teeth, Test Dataset 24: Digital radiograph of lower posterior teeth, Test Dataset 25: Digital radiograph of upper posterior teeth, Test Dataset 26: Digital radiograph of lower deciduous posterior teeth, Test Dataset 27: Digital radiograph of lower deciduous posterior teeth, Test Dataset 28: Digital radiograph of lower posterior teeth, Test Dataset 29: Digital radiograph of lower posterior teeth, Test Dataset 30: Digital radiograph of upper deciduous posterior teeth, Test Dataset 31: Digital radiograph of upper anterior teeth, Test Dataset 32: Digital radiograph of lower [file 12903_2023_3251_MOESM1_ESM.zip › Test Dataset 36.jpg]

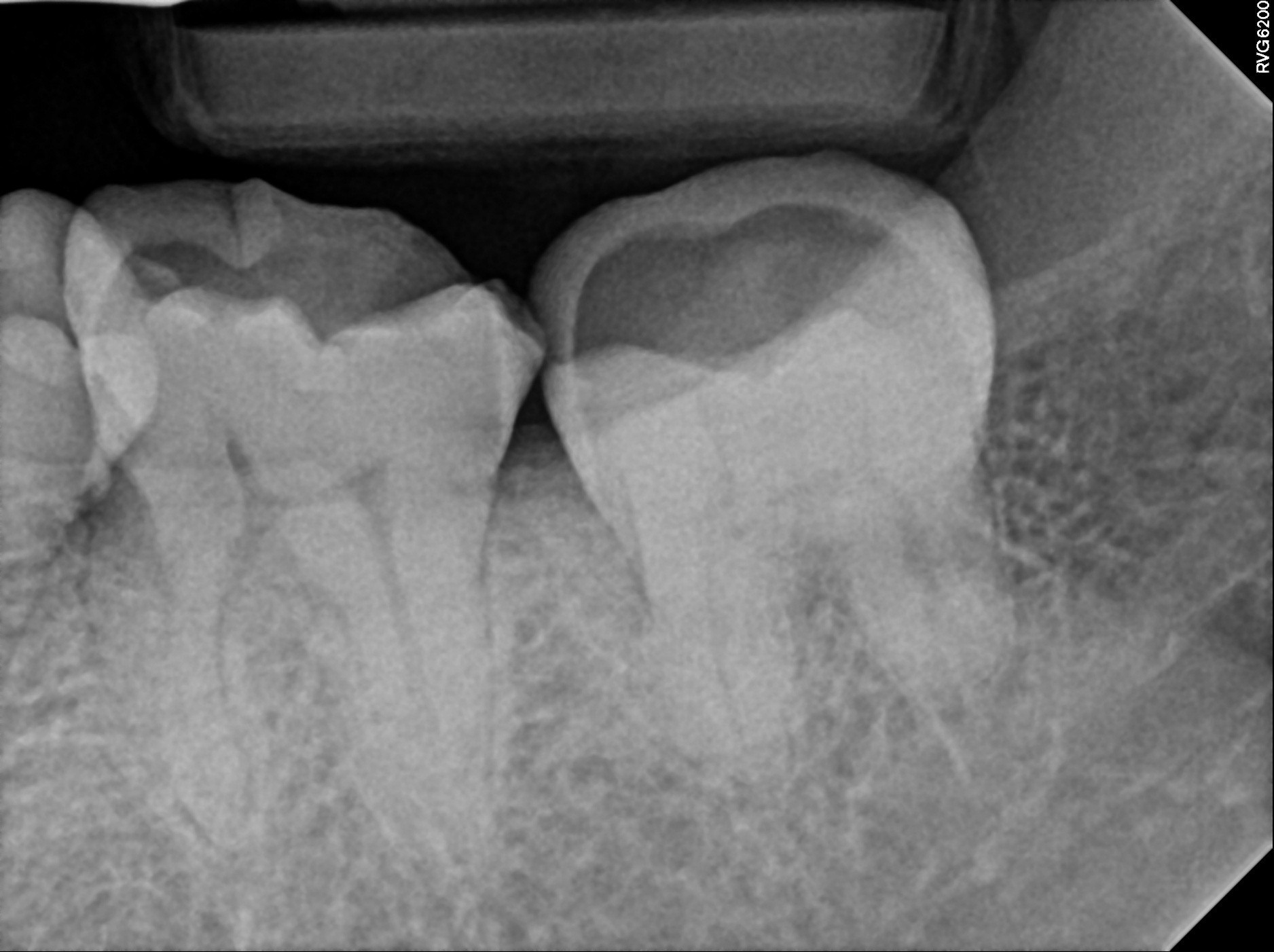

Supplement: Supplementary file 1 — Additional file 1: Test Dataset 1: Digital radiograph of upper posterior teeth. Test Dataset 2: Digital radiograph of upper posterior teeth, Test Dataset 3: Digital radiograph of upper posterior teeth, Test Dataset 4: Digital radiograph of upper posterior teeth, Test Dataset 5: Digital radiograph of upper anterior teeth, Test Dataset 6: Digital radiograph of upper anterior teeth, Test Dataset 7: Digital radiograph of lower posterior teeth, Test Dataset 8: Digital radiograph of upper posterior teeth, Test Dataset 9: Digital radiograph of lower anterior teeth, Test Dataset 10: Digital radiograph of lower anterior teeth, Test Dataset 11: Digital radiograph of lower posterior teeth, Test Dataset 12: Digital radiograph of lower anterior teeth, Test Dataset 13: Digital radiograph of upper posterior teeth, Test Dataset 14: Digital radiograph of lower teeth, Test Dataset 15: Digital radiograph of lower deciduous teeth, Test Dataset 16: Digital radiograph of lower deciduous teeth, Test Dataset 17: Digital radiograph of lower posterior teeth, Test Dataset 18: Digital radiograph of lower deciduous posterior teeth, Test Dataset 19: Digital radiograph of upper posterior teeth, Test Dataset 20: Digital radiograph of lower posterior teeth, Test Dataset 21: Digital radiograph of lower posterior teeth, Test Dataset 22: Digital radiograph of upper posterior teeth, Test Dataset 23: Digital radiograph of upper posterior teeth, Test Dataset 24: Digital radiograph of lower posterior teeth, Test Dataset 25: Digital radiograph of upper posterior teeth, Test Dataset 26: Digital radiograph of lower deciduous posterior teeth, Test Dataset 27: Digital radiograph of lower deciduous posterior teeth, Test Dataset 28: Digital radiograph of lower posterior teeth, Test Dataset 29: Digital radiograph of lower posterior teeth, Test Dataset 30: Digital radiograph of upper deciduous posterior teeth, Test Dataset 31: Digital radiograph of upper anterior teeth, Test Dataset 32: Digital radiograph of lower [file 12903_2023_3251_MOESM1_ESM.zip › Test Dataset 37.jpg]

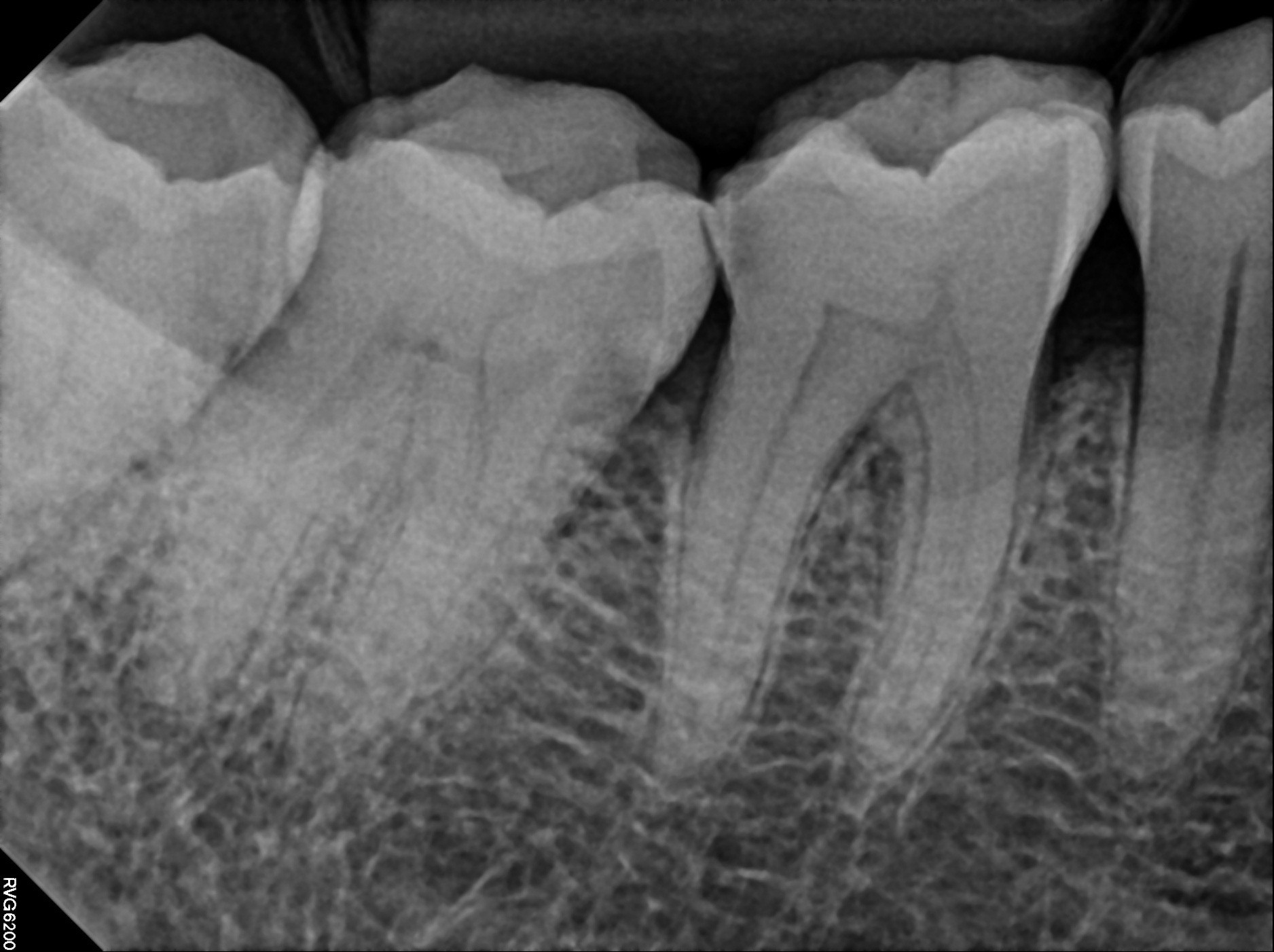

Supplement: Supplementary file 1 — Additional file 1: Test Dataset 1: Digital radiograph of upper posterior teeth. Test Dataset 2: Digital radiograph of upper posterior teeth, Test Dataset 3: Digital radiograph of upper posterior teeth, Test Dataset 4: Digital radiograph of upper posterior teeth, Test Dataset 5: Digital radiograph of upper anterior teeth, Test Dataset 6: Digital radiograph of upper anterior teeth, Test Dataset 7: Digital radiograph of lower posterior teeth, Test Dataset 8: Digital radiograph of upper posterior teeth, Test Dataset 9: Digital radiograph of lower anterior teeth, Test Dataset 10: Digital radiograph of lower anterior teeth, Test Dataset 11: Digital radiograph of lower posterior teeth, Test Dataset 12: Digital radiograph of lower anterior teeth, Test Dataset 13: Digital radiograph of upper posterior teeth, Test Dataset 14: Digital radiograph of lower teeth, Test Dataset 15: Digital radiograph of lower deciduous teeth, Test Dataset 16: Digital radiograph of lower deciduous teeth, Test Dataset 17: Digital radiograph of lower posterior teeth, Test Dataset 18: Digital radiograph of lower deciduous posterior teeth, Test Dataset 19: Digital radiograph of upper posterior teeth, Test Dataset 20: Digital radiograph of lower posterior teeth, Test Dataset 21: Digital radiograph of lower posterior teeth, Test Dataset 22: Digital radiograph of upper posterior teeth, Test Dataset 23: Digital radiograph of upper posterior teeth, Test Dataset 24: Digital radiograph of lower posterior teeth, Test Dataset 25: Digital radiograph of upper posterior teeth, Test Dataset 26: Digital radiograph of lower deciduous posterior teeth, Test Dataset 27: Digital radiograph of lower deciduous posterior teeth, Test Dataset 28: Digital radiograph of lower posterior teeth, Test Dataset 29: Digital radiograph of lower posterior teeth, Test Dataset 30: Digital radiograph of upper deciduous posterior teeth, Test Dataset 31: Digital radiograph of upper anterior teeth, Test Dataset 32: Digital radiograph of lower [file 12903_2023_3251_MOESM1_ESM.zip › Test Dataset 38.jpg]

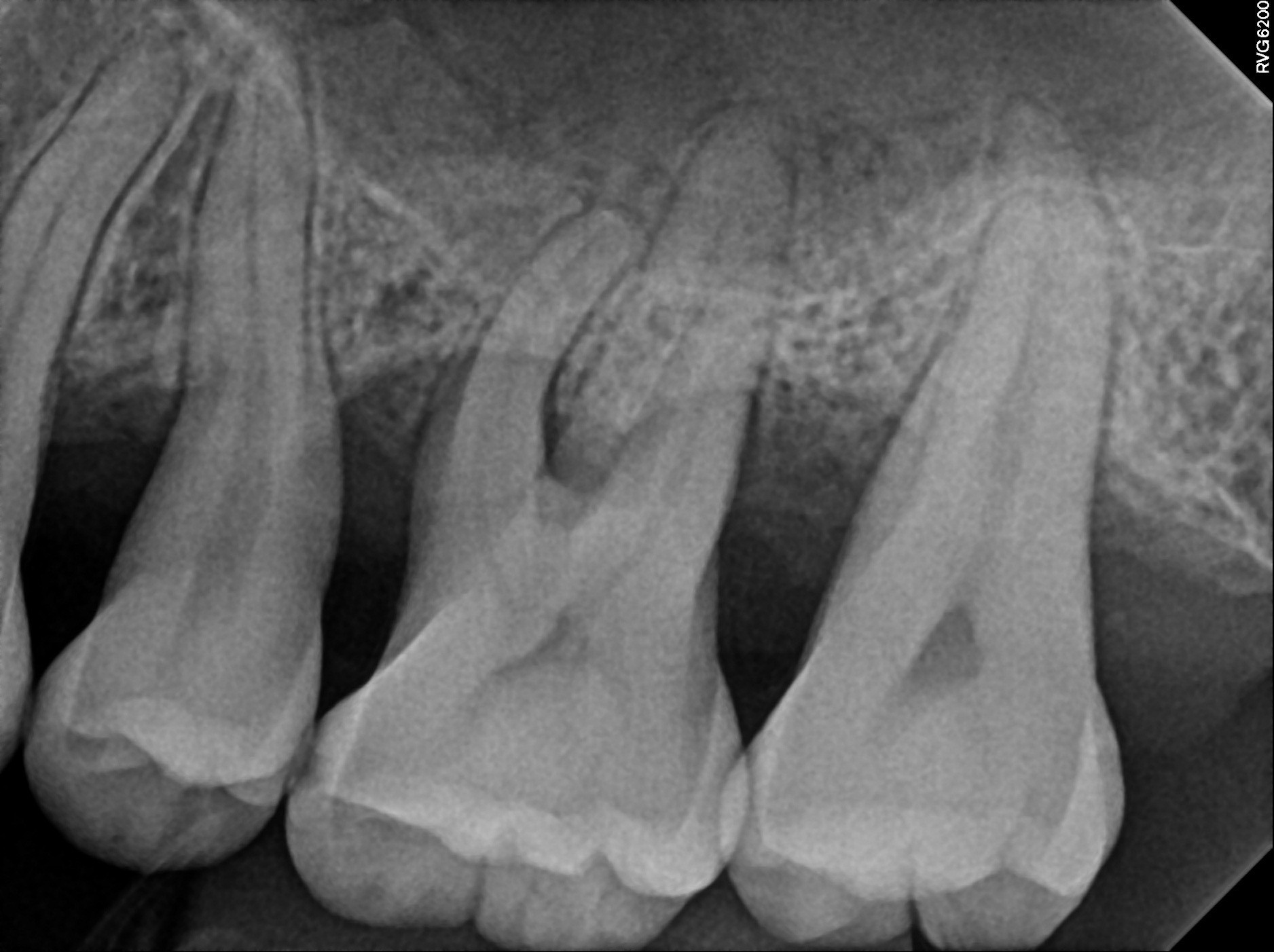

Supplement: Supplementary file 1 — Additional file 1: Test Dataset 1: Digital radiograph of upper posterior teeth. Test Dataset 2: Digital radiograph of upper posterior teeth, Test Dataset 3: Digital radiograph of upper posterior teeth, Test Dataset 4: Digital radiograph of upper posterior teeth, Test Dataset 5: Digital radiograph of upper anterior teeth, Test Dataset 6: Digital radiograph of upper anterior teeth, Test Dataset 7: Digital radiograph of lower posterior teeth, Test Dataset 8: Digital radiograph of upper posterior teeth, Test Dataset 9: Digital radiograph of lower anterior teeth, Test Dataset 10: Digital radiograph of lower anterior teeth, Test Dataset 11: Digital radiograph of lower posterior teeth, Test Dataset 12: Digital radiograph of lower anterior teeth, Test Dataset 13: Digital radiograph of upper posterior teeth, Test Dataset 14: Digital radiograph of lower teeth, Test Dataset 15: Digital radiograph of lower deciduous teeth, Test Dataset 16: Digital radiograph of lower deciduous teeth, Test Dataset 17: Digital radiograph of lower posterior teeth, Test Dataset 18: Digital radiograph of lower deciduous posterior teeth, Test Dataset 19: Digital radiograph of upper posterior teeth, Test Dataset 20: Digital radiograph of lower posterior teeth, Test Dataset 21: Digital radiograph of lower posterior teeth, Test Dataset 22: Digital radiograph of upper posterior teeth, Test Dataset 23: Digital radiograph of upper posterior teeth, Test Dataset 24: Digital radiograph of lower posterior teeth, Test Dataset 25: Digital radiograph of upper posterior teeth, Test Dataset 26: Digital radiograph of lower deciduous posterior teeth, Test Dataset 27: Digital radiograph of lower deciduous posterior teeth, Test Dataset 28: Digital radiograph of lower posterior teeth, Test Dataset 29: Digital radiograph of lower posterior teeth, Test Dataset 30: Digital radiograph of upper deciduous posterior teeth, Test Dataset 31: Digital radiograph of upper anterior teeth, Test Dataset 32: Digital radiograph of lower [file 12903_2023_3251_MOESM1_ESM.zip › Test Dataset 39.jpg]

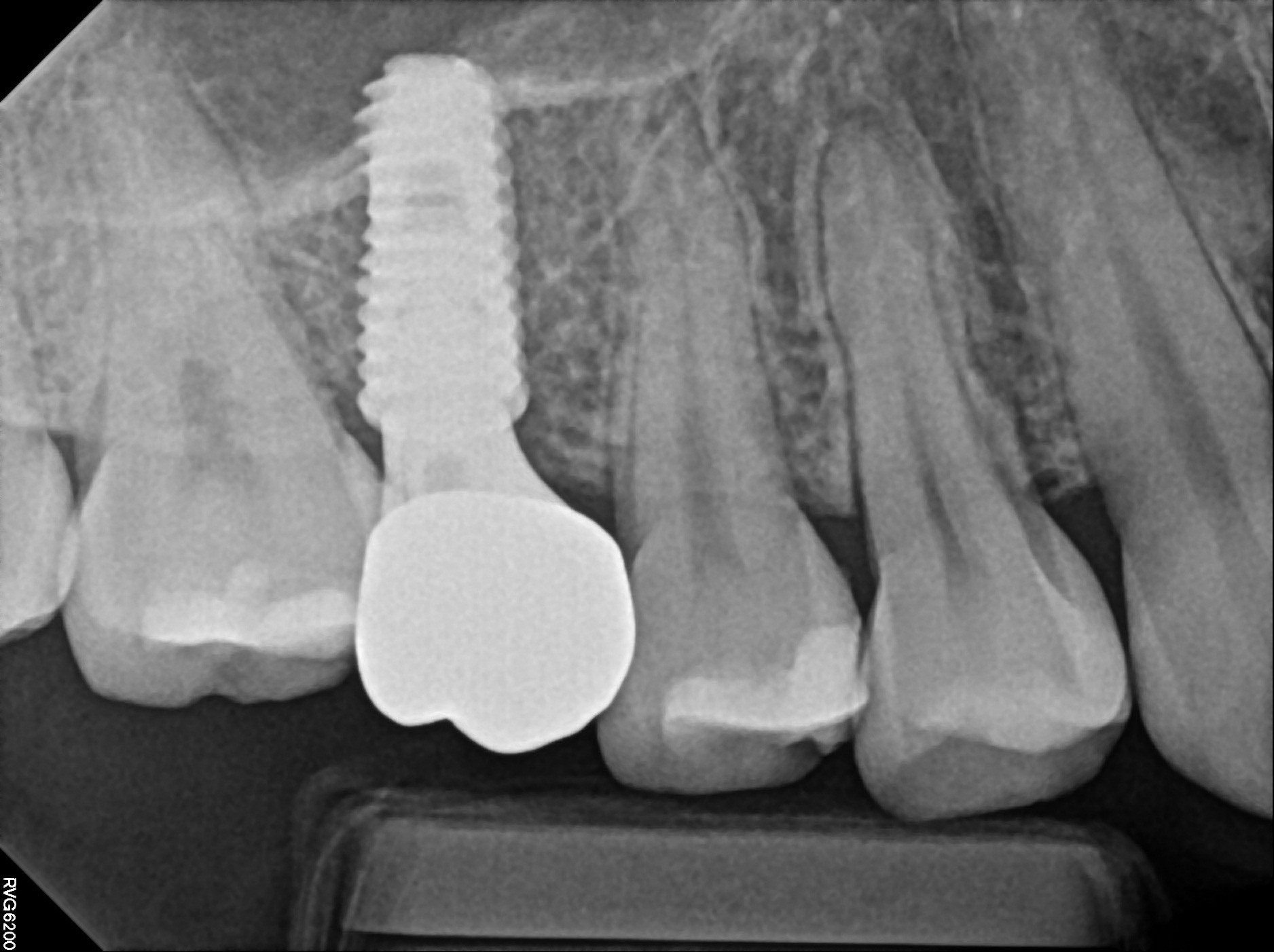

Supplement: Supplementary file 1 — Additional file 1: Test Dataset 1: Digital radiograph of upper posterior teeth. Test Dataset 2: Digital radiograph of upper posterior teeth, Test Dataset 3: Digital radiograph of upper posterior teeth, Test Dataset 4: Digital radiograph of upper posterior teeth, Test Dataset 5: Digital radiograph of upper anterior teeth, Test Dataset 6: Digital radiograph of upper anterior teeth, Test Dataset 7: Digital radiograph of lower posterior teeth, Test Dataset 8: Digital radiograph of upper posterior teeth, Test Dataset 9: Digital radiograph of lower anterior teeth, Test Dataset 10: Digital radiograph of lower anterior teeth, Test Dataset 11: Digital radiograph of lower posterior teeth, Test Dataset 12: Digital radiograph of lower anterior teeth, Test Dataset 13: Digital radiograph of upper posterior teeth, Test Dataset 14: Digital radiograph of lower teeth, Test Dataset 15: Digital radiograph of lower deciduous teeth, Test Dataset 16: Digital radiograph of lower deciduous teeth, Test Dataset 17: Digital radiograph of lower posterior teeth, Test Dataset 18: Digital radiograph of lower deciduous posterior teeth, Test Dataset 19: Digital radiograph of upper posterior teeth, Test Dataset 20: Digital radiograph of lower posterior teeth, Test Dataset 21: Digital radiograph of lower posterior teeth, Test Dataset 22: Digital radiograph of upper posterior teeth, Test Dataset 23: Digital radiograph of upper posterior teeth, Test Dataset 24: Digital radiograph of lower posterior teeth, Test Dataset 25: Digital radiograph of upper posterior teeth, Test Dataset 26: Digital radiograph of lower deciduous posterior teeth, Test Dataset 27: Digital radiograph of lower deciduous posterior teeth, Test Dataset 28: Digital radiograph of lower posterior teeth, Test Dataset 29: Digital radiograph of lower posterior teeth, Test Dataset 30: Digital radiograph of upper deciduous posterior teeth, Test Dataset 31: Digital radiograph of upper anterior teeth, Test Dataset 32: Digital radiograph of lower [file 12903_2023_3251_MOESM1_ESM.zip › Test Dataset 4.jpg]

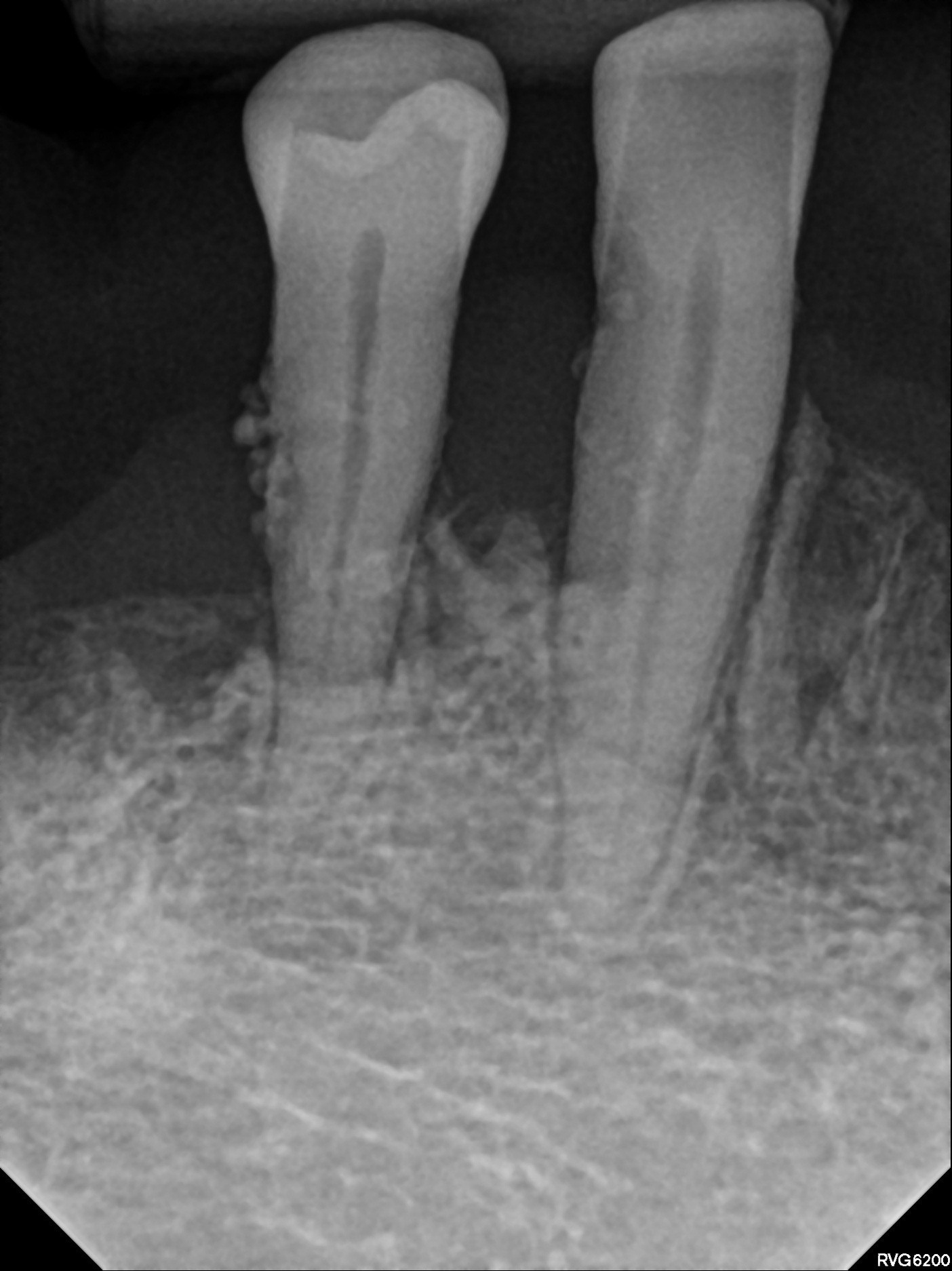

Supplement: Supplementary file 1 — Additional file 1: Test Dataset 1: Digital radiograph of upper posterior teeth. Test Dataset 2: Digital radiograph of upper posterior teeth, Test Dataset 3: Digital radiograph of upper posterior teeth, Test Dataset 4: Digital radiograph of upper posterior teeth, Test Dataset 5: Digital radiograph of upper anterior teeth, Test Dataset 6: Digital radiograph of upper anterior teeth, Test Dataset 7: Digital radiograph of lower posterior teeth, Test Dataset 8: Digital radiograph of upper posterior teeth, Test Dataset 9: Digital radiograph of lower anterior teeth, Test Dataset 10: Digital radiograph of lower anterior teeth, Test Dataset 11: Digital radiograph of lower posterior teeth, Test Dataset 12: Digital radiograph of lower anterior teeth, Test Dataset 13: Digital radiograph of upper posterior teeth, Test Dataset 14: Digital radiograph of lower teeth, Test Dataset 15: Digital radiograph of lower deciduous teeth, Test Dataset 16: Digital radiograph of lower deciduous teeth, Test Dataset 17: Digital radiograph of lower posterior teeth, Test Dataset 18: Digital radiograph of lower deciduous posterior teeth, Test Dataset 19: Digital radiograph of upper posterior teeth, Test Dataset 20: Digital radiograph of lower posterior teeth, Test Dataset 21: Digital radiograph of lower posterior teeth, Test Dataset 22: Digital radiograph of upper posterior teeth, Test Dataset 23: Digital radiograph of upper posterior teeth, Test Dataset 24: Digital radiograph of lower posterior teeth, Test Dataset 25: Digital radiograph of upper posterior teeth, Test Dataset 26: Digital radiograph of lower deciduous posterior teeth, Test Dataset 27: Digital radiograph of lower deciduous posterior teeth, Test Dataset 28: Digital radiograph of lower posterior teeth, Test Dataset 29: Digital radiograph of lower posterior teeth, Test Dataset 30: Digital radiograph of upper deciduous posterior teeth, Test Dataset 31: Digital radiograph of upper anterior teeth, Test Dataset 32: Digital radiograph of lower [file 12903_2023_3251_MOESM1_ESM.zip › Test Dataset 40.jpg]

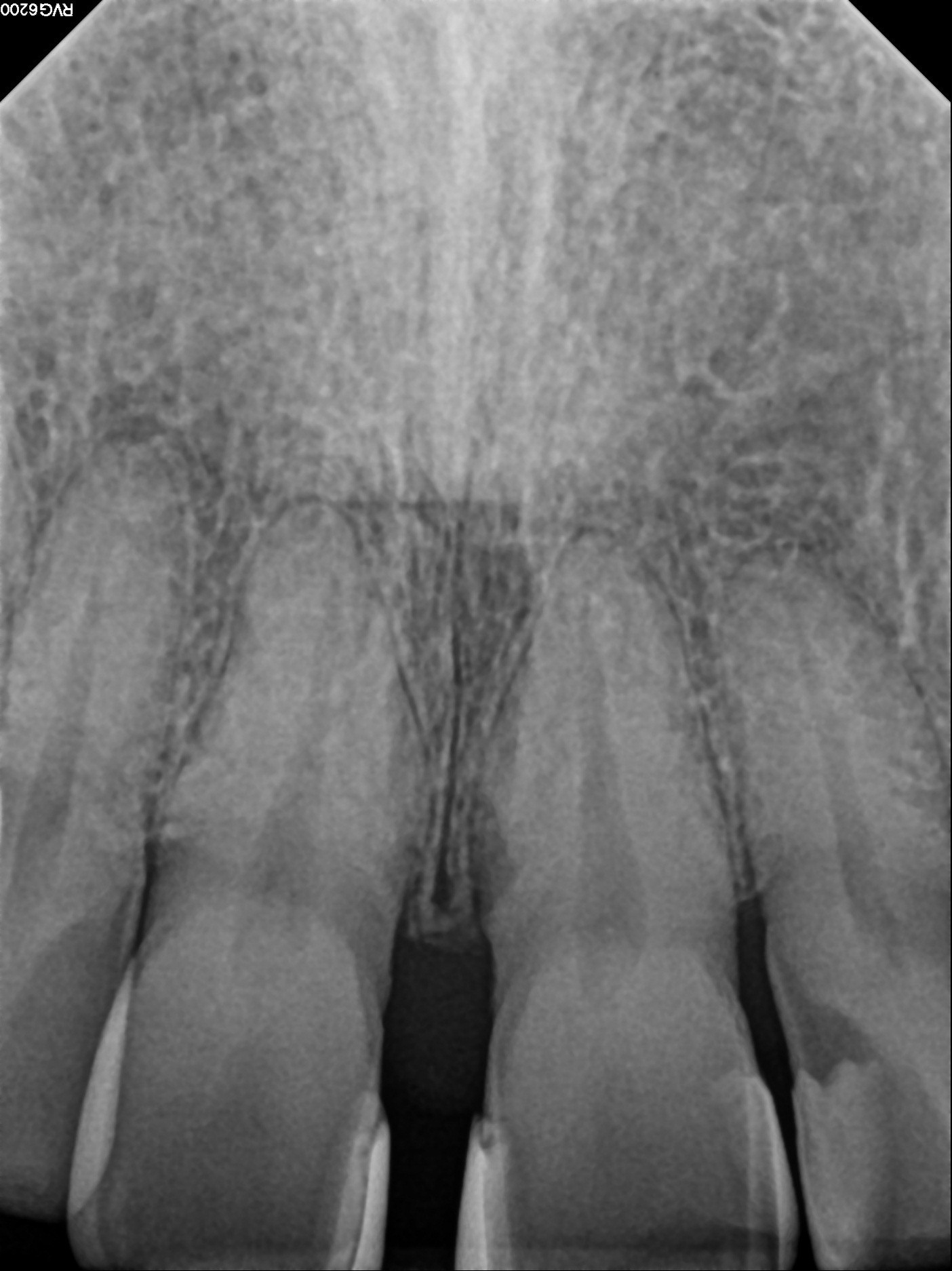

Supplement: Supplementary file 1 — Additional file 1: Test Dataset 1: Digital radiograph of upper posterior teeth. Test Dataset 2: Digital radiograph of upper posterior teeth, Test Dataset 3: Digital radiograph of upper posterior teeth, Test Dataset 4: Digital radiograph of upper posterior teeth, Test Dataset 5: Digital radiograph of upper anterior teeth, Test Dataset 6: Digital radiograph of upper anterior teeth, Test Dataset 7: Digital radiograph of lower posterior teeth, Test Dataset 8: Digital radiograph of upper posterior teeth, Test Dataset 9: Digital radiograph of lower anterior teeth, Test Dataset 10: Digital radiograph of lower anterior teeth, Test Dataset 11: Digital radiograph of lower posterior teeth, Test Dataset 12: Digital radiograph of lower anterior teeth, Test Dataset 13: Digital radiograph of upper posterior teeth, Test Dataset 14: Digital radiograph of lower teeth, Test Dataset 15: Digital radiograph of lower deciduous teeth, Test Dataset 16: Digital radiograph of lower deciduous teeth, Test Dataset 17: Digital radiograph of lower posterior teeth, Test Dataset 18: Digital radiograph of lower deciduous posterior teeth, Test Dataset 19: Digital radiograph of upper posterior teeth, Test Dataset 20: Digital radiograph of lower posterior teeth, Test Dataset 21: Digital radiograph of lower posterior teeth, Test Dataset 22: Digital radiograph of upper posterior teeth, Test Dataset 23: Digital radiograph of upper posterior teeth, Test Dataset 24: Digital radiograph of lower posterior teeth, Test Dataset 25: Digital radiograph of upper posterior teeth, Test Dataset 26: Digital radiograph of lower deciduous posterior teeth, Test Dataset 27: Digital radiograph of lower deciduous posterior teeth, Test Dataset 28: Digital radiograph of lower posterior teeth, Test Dataset 29: Digital radiograph of lower posterior teeth, Test Dataset 30: Digital radiograph of upper deciduous posterior teeth, Test Dataset 31: Digital radiograph of upper anterior teeth, Test Dataset 32: Digital radiograph of lower [file 12903_2023_3251_MOESM1_ESM.zip › Test Dataset 41.jpg]

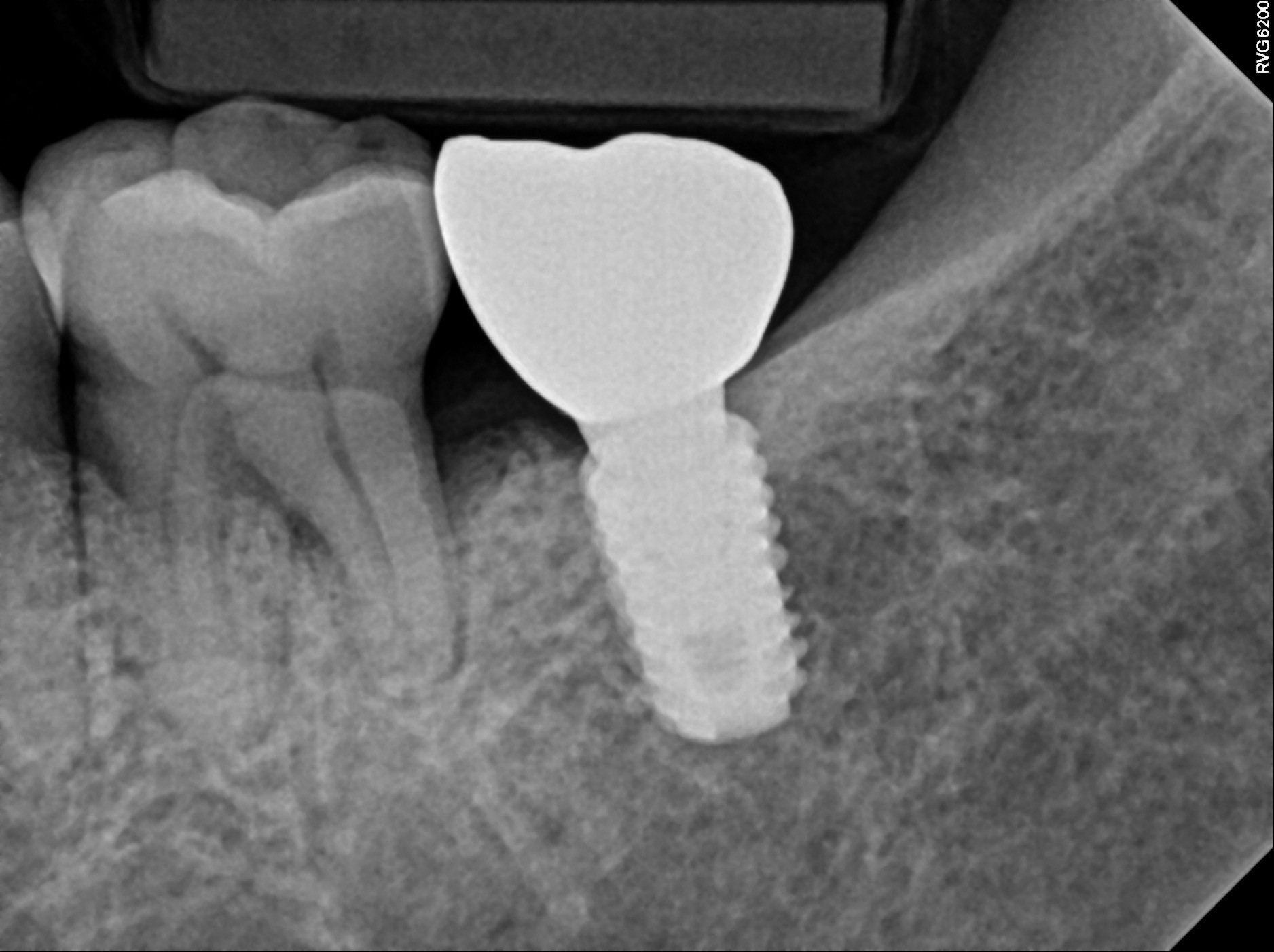

Supplement: Supplementary file 1 — Additional file 1: Test Dataset 1: Digital radiograph of upper posterior teeth. Test Dataset 2: Digital radiograph of upper posterior teeth, Test Dataset 3: Digital radiograph of upper posterior teeth, Test Dataset 4: Digital radiograph of upper posterior teeth, Test Dataset 5: Digital radiograph of upper anterior teeth, Test Dataset 6: Digital radiograph of upper anterior teeth, Test Dataset 7: Digital radiograph of lower posterior teeth, Test Dataset 8: Digital radiograph of upper posterior teeth, Test Dataset 9: Digital radiograph of lower anterior teeth, Test Dataset 10: Digital radiograph of lower anterior teeth, Test Dataset 11: Digital radiograph of lower posterior teeth, Test Dataset 12: Digital radiograph of lower anterior teeth, Test Dataset 13: Digital radiograph of upper posterior teeth, Test Dataset 14: Digital radiograph of lower teeth, Test Dataset 15: Digital radiograph of lower deciduous teeth, Test Dataset 16: Digital radiograph of lower deciduous teeth, Test Dataset 17: Digital radiograph of lower posterior teeth, Test Dataset 18: Digital radiograph of lower deciduous posterior teeth, Test Dataset 19: Digital radiograph of upper posterior teeth, Test Dataset 20: Digital radiograph of lower posterior teeth, Test Dataset 21: Digital radiograph of lower posterior teeth, Test Dataset 22: Digital radiograph of upper posterior teeth, Test Dataset 23: Digital radiograph of upper posterior teeth, Test Dataset 24: Digital radiograph of lower posterior teeth, Test Dataset 25: Digital radiograph of upper posterior teeth, Test Dataset 26: Digital radiograph of lower deciduous posterior teeth, Test Dataset 27: Digital radiograph of lower deciduous posterior teeth, Test Dataset 28: Digital radiograph of lower posterior teeth, Test Dataset 29: Digital radiograph of lower posterior teeth, Test Dataset 30: Digital radiograph of upper deciduous posterior teeth, Test Dataset 31: Digital radiograph of upper anterior teeth, Test Dataset 32: Digital radiograph of lower [file 12903_2023_3251_MOESM1_ESM.zip › Test Dataset 42.jpg]

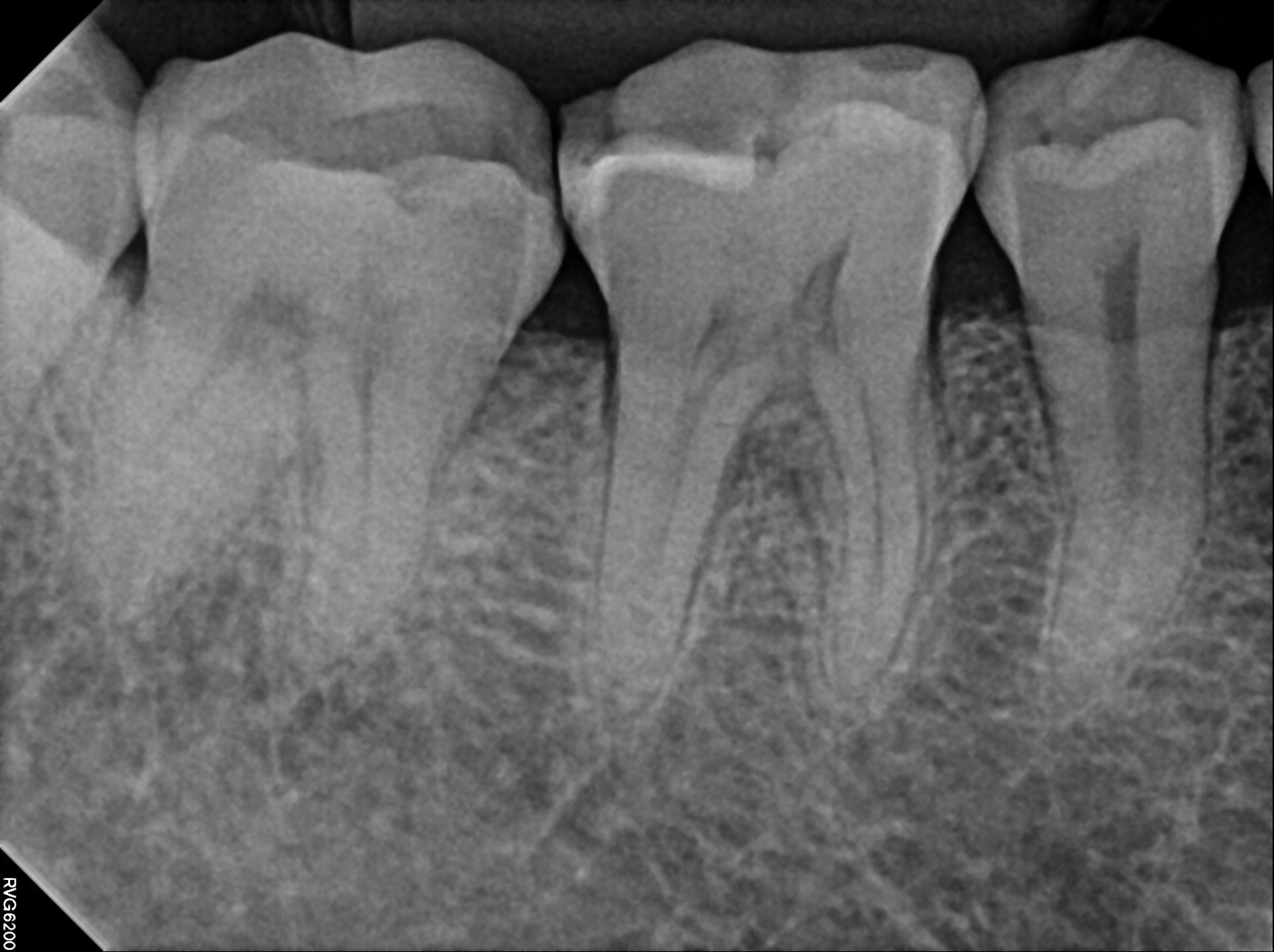

Supplement: Supplementary file 1 — Additional file 1: Test Dataset 1: Digital radiograph of upper posterior teeth. Test Dataset 2: Digital radiograph of upper posterior teeth, Test Dataset 3: Digital radiograph of upper posterior teeth, Test Dataset 4: Digital radiograph of upper posterior teeth, Test Dataset 5: Digital radiograph of upper anterior teeth, Test Dataset 6: Digital radiograph of upper anterior teeth, Test Dataset 7: Digital radiograph of lower posterior teeth, Test Dataset 8: Digital radiograph of upper posterior teeth, Test Dataset 9: Digital radiograph of lower anterior teeth, Test Dataset 10: Digital radiograph of lower anterior teeth, Test Dataset 11: Digital radiograph of lower posterior teeth, Test Dataset 12: Digital radiograph of lower anterior teeth, Test Dataset 13: Digital radiograph of upper posterior teeth, Test Dataset 14: Digital radiograph of lower teeth, Test Dataset 15: Digital radiograph of lower deciduous teeth, Test Dataset 16: Digital radiograph of lower deciduous teeth, Test Dataset 17: Digital radiograph of lower posterior teeth, Test Dataset 18: Digital radiograph of lower deciduous posterior teeth, Test Dataset 19: Digital radiograph of upper posterior teeth, Test Dataset 20: Digital radiograph of lower posterior teeth, Test Dataset 21: Digital radiograph of lower posterior teeth, Test Dataset 22: Digital radiograph of upper posterior teeth, Test Dataset 23: Digital radiograph of upper posterior teeth, Test Dataset 24: Digital radiograph of lower posterior teeth, Test Dataset 25: Digital radiograph of upper posterior teeth, Test Dataset 26: Digital radiograph of lower deciduous posterior teeth, Test Dataset 27: Digital radiograph of lower deciduous posterior teeth, Test Dataset 28: Digital radiograph of lower posterior teeth, Test Dataset 29: Digital radiograph of lower posterior teeth, Test Dataset 30: Digital radiograph of upper deciduous posterior teeth, Test Dataset 31: Digital radiograph of upper anterior teeth, Test Dataset 32: Digital radiograph of lower [file 12903_2023_3251_MOESM1_ESM.zip › Test Dataset 43.jpg]

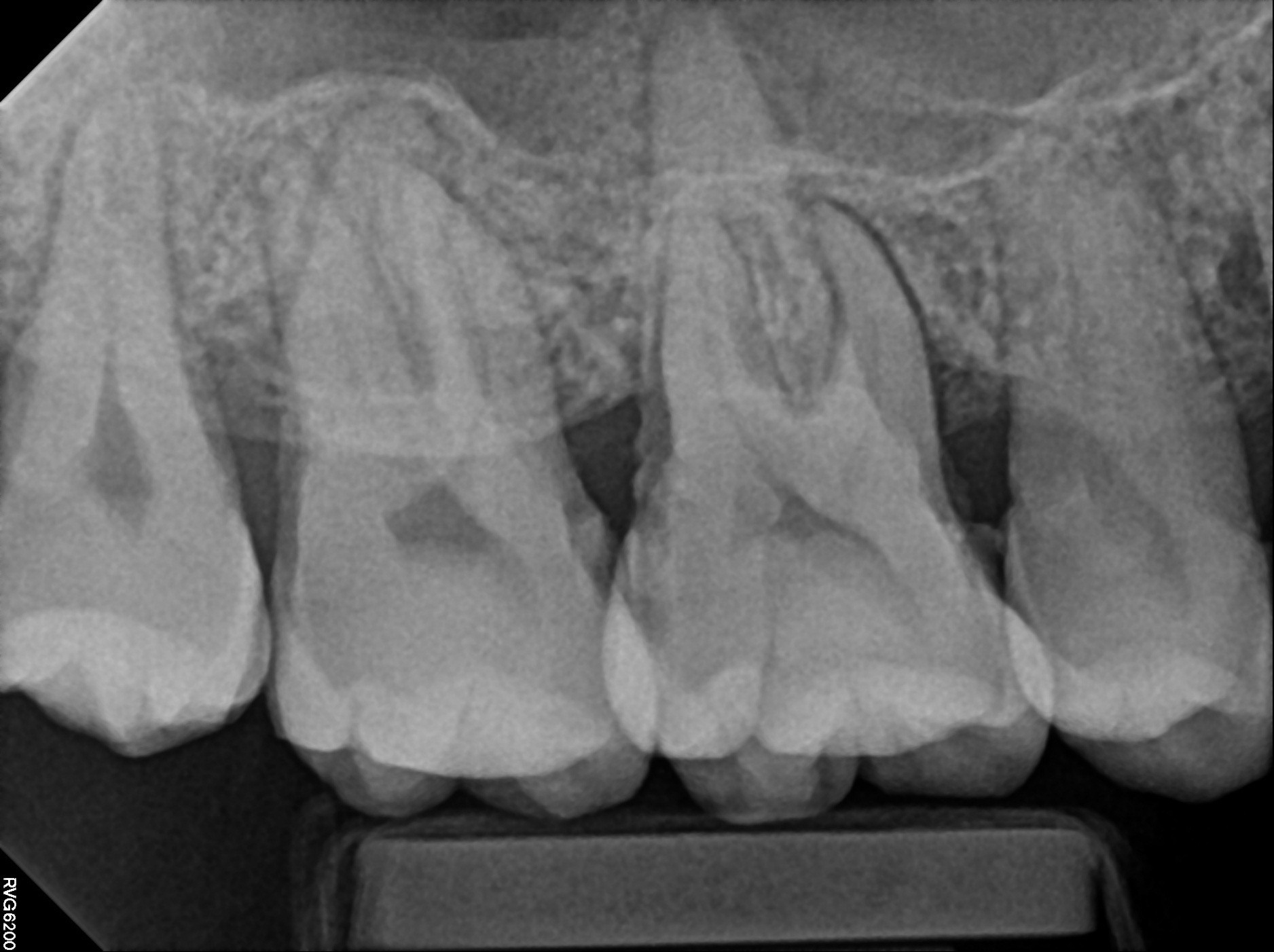

Supplement: Supplementary file 1 — Additional file 1: Test Dataset 1: Digital radiograph of upper posterior teeth. Test Dataset 2: Digital radiograph of upper posterior teeth, Test Dataset 3: Digital radiograph of upper posterior teeth, Test Dataset 4: Digital radiograph of upper posterior teeth, Test Dataset 5: Digital radiograph of upper anterior teeth, Test Dataset 6: Digital radiograph of upper anterior teeth, Test Dataset 7: Digital radiograph of lower posterior teeth, Test Dataset 8: Digital radiograph of upper posterior teeth, Test Dataset 9: Digital radiograph of lower anterior teeth, Test Dataset 10: Digital radiograph of lower anterior teeth, Test Dataset 11: Digital radiograph of lower posterior teeth, Test Dataset 12: Digital radiograph of lower anterior teeth, Test Dataset 13: Digital radiograph of upper posterior teeth, Test Dataset 14: Digital radiograph of lower teeth, Test Dataset 15: Digital radiograph of lower deciduous teeth, Test Dataset 16: Digital radiograph of lower deciduous teeth, Test Dataset 17: Digital radiograph of lower posterior teeth, Test Dataset 18: Digital radiograph of lower deciduous posterior teeth, Test Dataset 19: Digital radiograph of upper posterior teeth, Test Dataset 20: Digital radiograph of lower posterior teeth, Test Dataset 21: Digital radiograph of lower posterior teeth, Test Dataset 22: Digital radiograph of upper posterior teeth, Test Dataset 23: Digital radiograph of upper posterior teeth, Test Dataset 24: Digital radiograph of lower posterior teeth, Test Dataset 25: Digital radiograph of upper posterior teeth, Test Dataset 26: Digital radiograph of lower deciduous posterior teeth, Test Dataset 27: Digital radiograph of lower deciduous posterior teeth, Test Dataset 28: Digital radiograph of lower posterior teeth, Test Dataset 29: Digital radiograph of lower posterior teeth, Test Dataset 30: Digital radiograph of upper deciduous posterior teeth, Test Dataset 31: Digital radiograph of upper anterior teeth, Test Dataset 32: Digital radiograph of lower [file 12903_2023_3251_MOESM1_ESM.zip › Test Dataset 44.jpg]

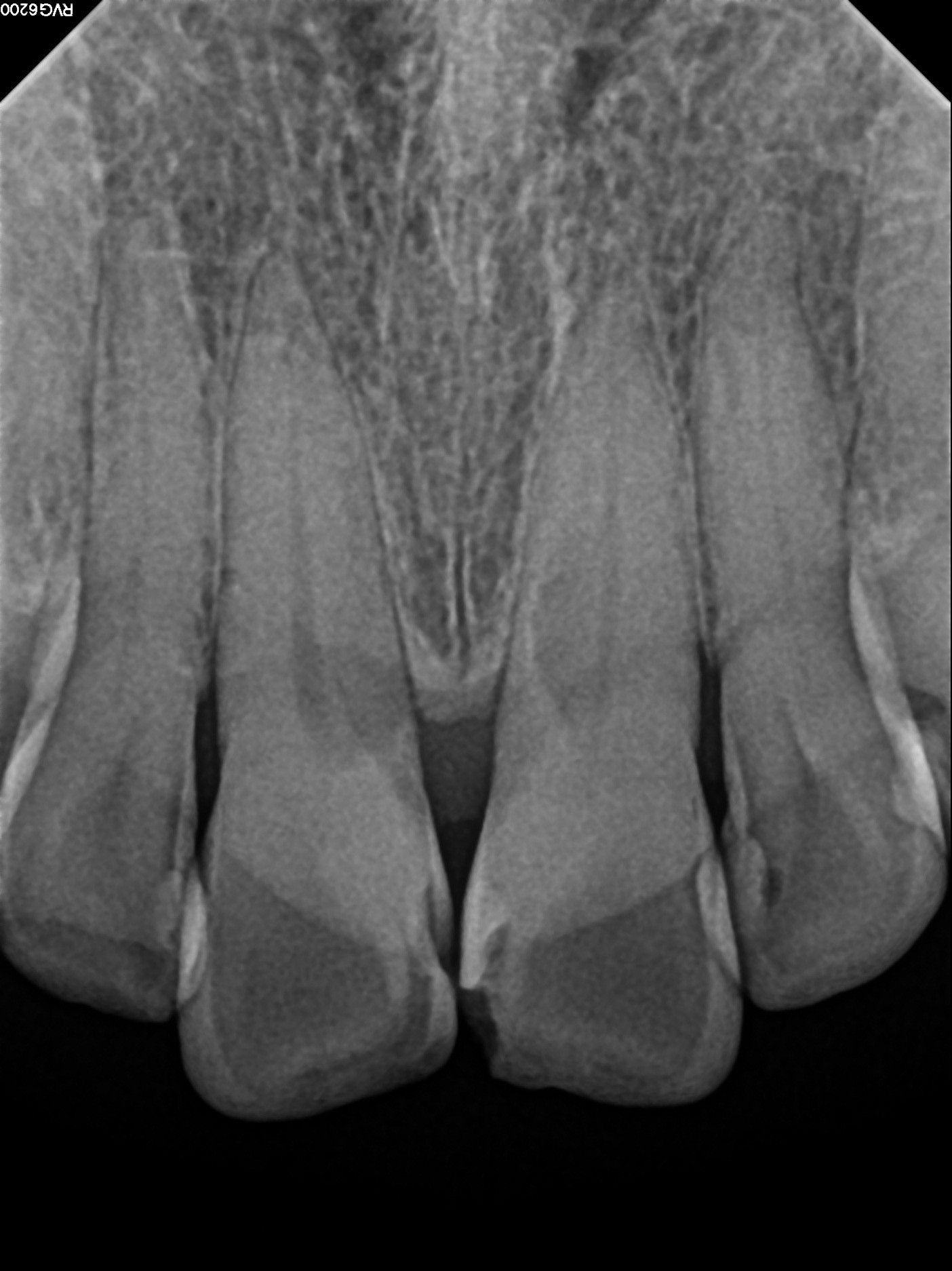

Supplement: Supplementary file 1 — Additional file 1: Test Dataset 1: Digital radiograph of upper posterior teeth. Test Dataset 2: Digital radiograph of upper posterior teeth, Test Dataset 3: Digital radiograph of upper posterior teeth, Test Dataset 4: Digital radiograph of upper posterior teeth, Test Dataset 5: Digital radiograph of upper anterior teeth, Test Dataset 6: Digital radiograph of upper anterior teeth, Test Dataset 7: Digital radiograph of lower posterior teeth, Test Dataset 8: Digital radiograph of upper posterior teeth, Test Dataset 9: Digital radiograph of lower anterior teeth, Test Dataset 10: Digital radiograph of lower anterior teeth, Test Dataset 11: Digital radiograph of lower posterior teeth, Test Dataset 12: Digital radiograph of lower anterior teeth, Test Dataset 13: Digital radiograph of upper posterior teeth, Test Dataset 14: Digital radiograph of lower teeth, Test Dataset 15: Digital radiograph of lower deciduous teeth, Test Dataset 16: Digital radiograph of lower deciduous teeth, Test Dataset 17: Digital radiograph of lower posterior teeth, Test Dataset 18: Digital radiograph of lower deciduous posterior teeth, Test Dataset 19: Digital radiograph of upper posterior teeth, Test Dataset 20: Digital radiograph of lower posterior teeth, Test Dataset 21: Digital radiograph of lower posterior teeth, Test Dataset 22: Digital radiograph of upper posterior teeth, Test Dataset 23: Digital radiograph of upper posterior teeth, Test Dataset 24: Digital radiograph of lower posterior teeth, Test Dataset 25: Digital radiograph of upper posterior teeth, Test Dataset 26: Digital radiograph of lower deciduous posterior teeth, Test Dataset 27: Digital radiograph of lower deciduous posterior teeth, Test Dataset 28: Digital radiograph of lower posterior teeth, Test Dataset 29: Digital radiograph of lower posterior teeth, Test Dataset 30: Digital radiograph of upper deciduous posterior teeth, Test Dataset 31: Digital radiograph of upper anterior teeth, Test Dataset 32: Digital radiograph of lower [file 12903_2023_3251_MOESM1_ESM.zip › Test Dataset 45.jpg]

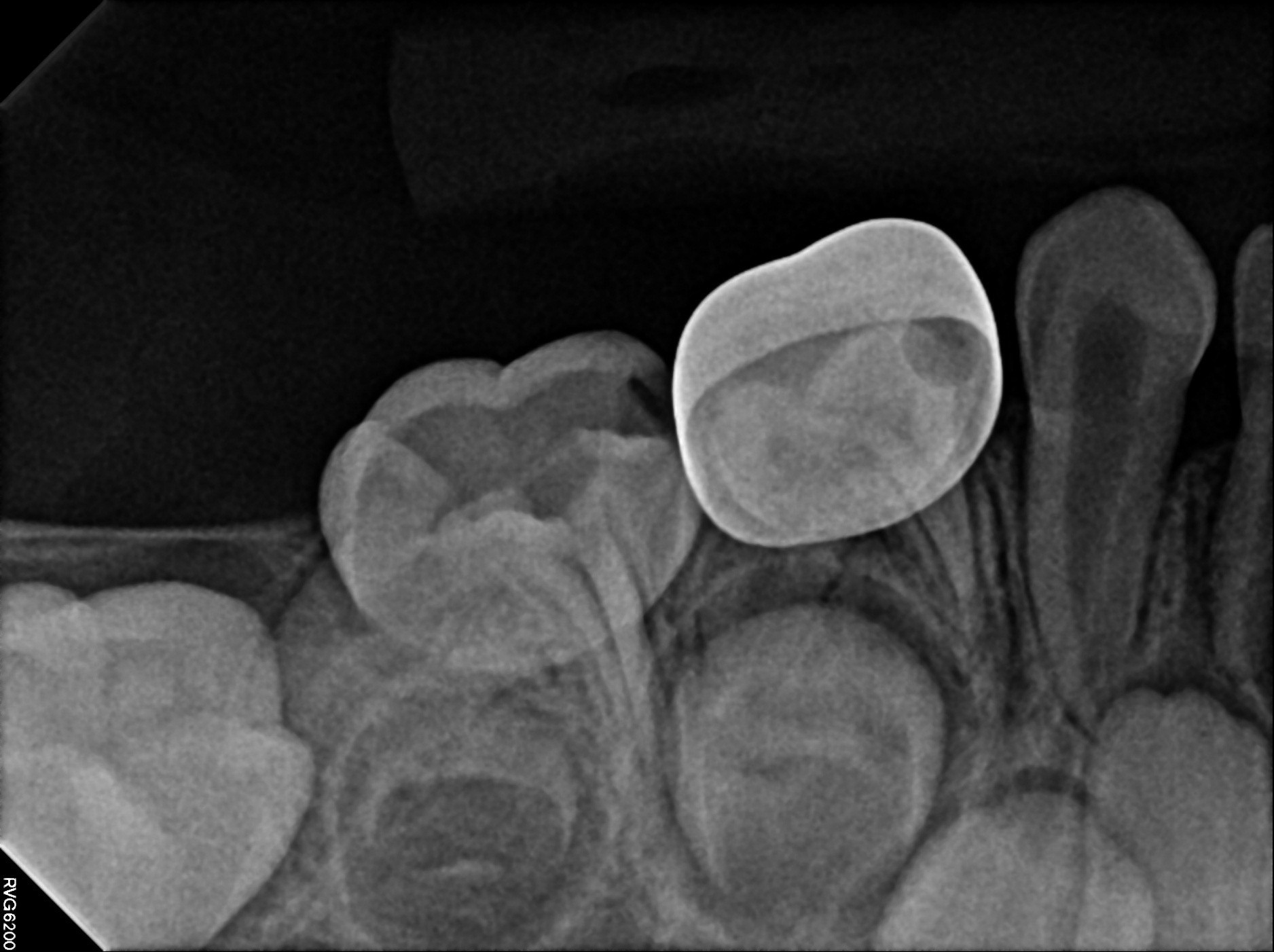

Supplement: Supplementary file 1 — Additional file 1: Test Dataset 1: Digital radiograph of upper posterior teeth. Test Dataset 2: Digital radiograph of upper posterior teeth, Test Dataset 3: Digital radiograph of upper posterior teeth, Test Dataset 4: Digital radiograph of upper posterior teeth, Test Dataset 5: Digital radiograph of upper anterior teeth, Test Dataset 6: Digital radiograph of upper anterior teeth, Test Dataset 7: Digital radiograph of lower posterior teeth, Test Dataset 8: Digital radiograph of upper posterior teeth, Test Dataset 9: Digital radiograph of lower anterior teeth, Test Dataset 10: Digital radiograph of lower anterior teeth, Test Dataset 11: Digital radiograph of lower posterior teeth, Test Dataset 12: Digital radiograph of lower anterior teeth, Test Dataset 13: Digital radiograph of upper posterior teeth, Test Dataset 14: Digital radiograph of lower teeth, Test Dataset 15: Digital radiograph of lower deciduous teeth, Test Dataset 16: Digital radiograph of lower deciduous teeth, Test Dataset 17: Digital radiograph of lower posterior teeth, Test Dataset 18: Digital radiograph of lower deciduous posterior teeth, Test Dataset 19: Digital radiograph of upper posterior teeth, Test Dataset 20: Digital radiograph of lower posterior teeth, Test Dataset 21: Digital radiograph of lower posterior teeth, Test Dataset 22: Digital radiograph of upper posterior teeth, Test Dataset 23: Digital radiograph of upper posterior teeth, Test Dataset 24: Digital radiograph of lower posterior teeth, Test Dataset 25: Digital radiograph of upper posterior teeth, Test Dataset 26: Digital radiograph of lower deciduous posterior teeth, Test Dataset 27: Digital radiograph of lower deciduous posterior teeth, Test Dataset 28: Digital radiograph of lower posterior teeth, Test Dataset 29: Digital radiograph of lower posterior teeth, Test Dataset 30: Digital radiograph of upper deciduous posterior teeth, Test Dataset 31: Digital radiograph of upper anterior teeth, Test Dataset 32: Digital radiograph of lower [file 12903_2023_3251_MOESM1_ESM.zip › Test Dataset 47.jpg]

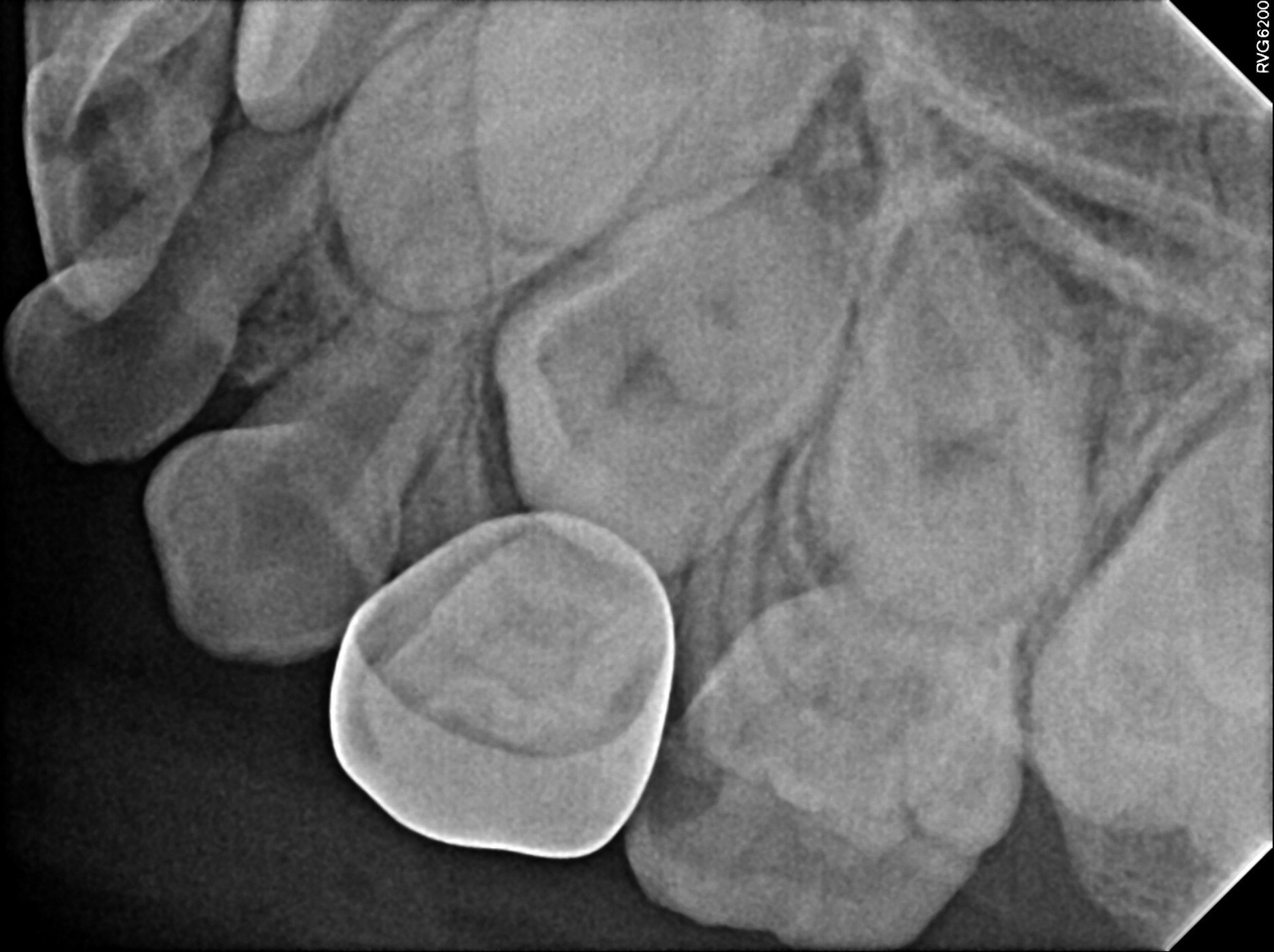

Supplement: Supplementary file 1 — Additional file 1: Test Dataset 1: Digital radiograph of upper posterior teeth. Test Dataset 2: Digital radiograph of upper posterior teeth, Test Dataset 3: Digital radiograph of upper posterior teeth, Test Dataset 4: Digital radiograph of upper posterior teeth, Test Dataset 5: Digital radiograph of upper anterior teeth, Test Dataset 6: Digital radiograph of upper anterior teeth, Test Dataset 7: Digital radiograph of lower posterior teeth, Test Dataset 8: Digital radiograph of upper posterior teeth, Test Dataset 9: Digital radiograph of lower anterior teeth, Test Dataset 10: Digital radiograph of lower anterior teeth, Test Dataset 11: Digital radiograph of lower posterior teeth, Test Dataset 12: Digital radiograph of lower anterior teeth, Test Dataset 13: Digital radiograph of upper posterior teeth, Test Dataset 14: Digital radiograph of lower teeth, Test Dataset 15: Digital radiograph of lower deciduous teeth, Test Dataset 16: Digital radiograph of lower deciduous teeth, Test Dataset 17: Digital radiograph of lower posterior teeth, Test Dataset 18: Digital radiograph of lower deciduous posterior teeth, Test Dataset 19: Digital radiograph of upper posterior teeth, Test Dataset 20: Digital radiograph of lower posterior teeth, Test Dataset 21: Digital radiograph of lower posterior teeth, Test Dataset 22: Digital radiograph of upper posterior teeth, Test Dataset 23: Digital radiograph of upper posterior teeth, Test Dataset 24: Digital radiograph of lower posterior teeth, Test Dataset 25: Digital radiograph of upper posterior teeth, Test Dataset 26: Digital radiograph of lower deciduous posterior teeth, Test Dataset 27: Digital radiograph of lower deciduous posterior teeth, Test Dataset 28: Digital radiograph of lower posterior teeth, Test Dataset 29: Digital radiograph of lower posterior teeth, Test Dataset 30: Digital radiograph of upper deciduous posterior teeth, Test Dataset 31: Digital radiograph of upper anterior teeth, Test Dataset 32: Digital radiograph of lower [file 12903_2023_3251_MOESM1_ESM.zip › Test Dataset 48.jpg]

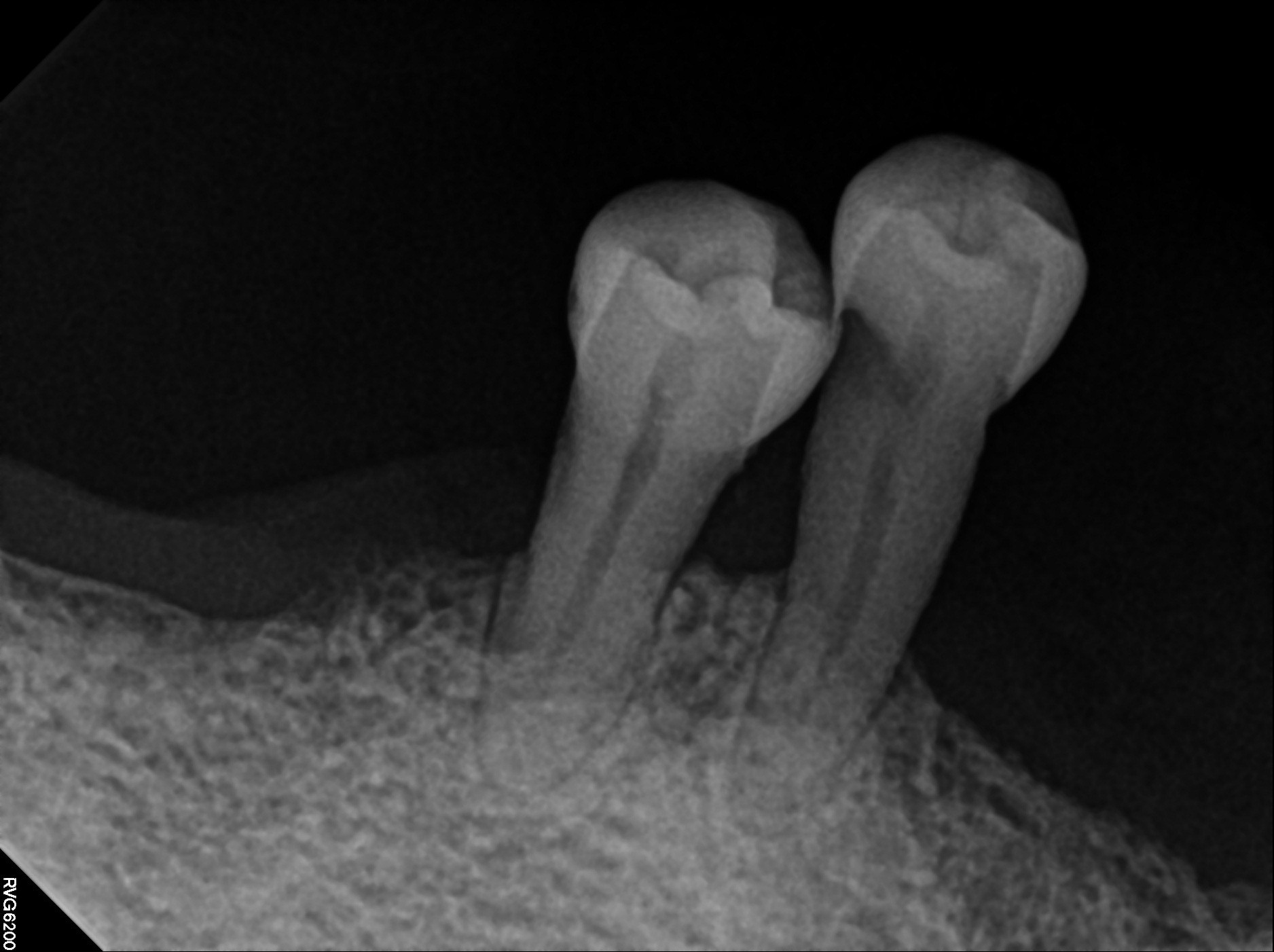

Supplement: Supplementary file 1 — Additional file 1: Test Dataset 1: Digital radiograph of upper posterior teeth. Test Dataset 2: Digital radiograph of upper posterior teeth, Test Dataset 3: Digital radiograph of upper posterior teeth, Test Dataset 4: Digital radiograph of upper posterior teeth, Test Dataset 5: Digital radiograph of upper anterior teeth, Test Dataset 6: Digital radiograph of upper anterior teeth, Test Dataset 7: Digital radiograph of lower posterior teeth, Test Dataset 8: Digital radiograph of upper posterior teeth, Test Dataset 9: Digital radiograph of lower anterior teeth, Test Dataset 10: Digital radiograph of lower anterior teeth, Test Dataset 11: Digital radiograph of lower posterior teeth, Test Dataset 12: Digital radiograph of lower anterior teeth, Test Dataset 13: Digital radiograph of upper posterior teeth, Test Dataset 14: Digital radiograph of lower teeth, Test Dataset 15: Digital radiograph of lower deciduous teeth, Test Dataset 16: Digital radiograph of lower deciduous teeth, Test Dataset 17: Digital radiograph of lower posterior teeth, Test Dataset 18: Digital radiograph of lower deciduous posterior teeth, Test Dataset 19: Digital radiograph of upper posterior teeth, Test Dataset 20: Digital radiograph of lower posterior teeth, Test Dataset 21: Digital radiograph of lower posterior teeth, Test Dataset 22: Digital radiograph of upper posterior teeth, Test Dataset 23: Digital radiograph of upper posterior teeth, Test Dataset 24: Digital radiograph of lower posterior teeth, Test Dataset 25: Digital radiograph of upper posterior teeth, Test Dataset 26: Digital radiograph of lower deciduous posterior teeth, Test Dataset 27: Digital radiograph of lower deciduous posterior teeth, Test Dataset 28: Digital radiograph of lower posterior teeth, Test Dataset 29: Digital radiograph of lower posterior teeth, Test Dataset 30: Digital radiograph of upper deciduous posterior teeth, Test Dataset 31: Digital radiograph of upper anterior teeth, Test Dataset 32: Digital radiograph of lower [file 12903_2023_3251_MOESM1_ESM.zip › Test Dataset 49.jpg]

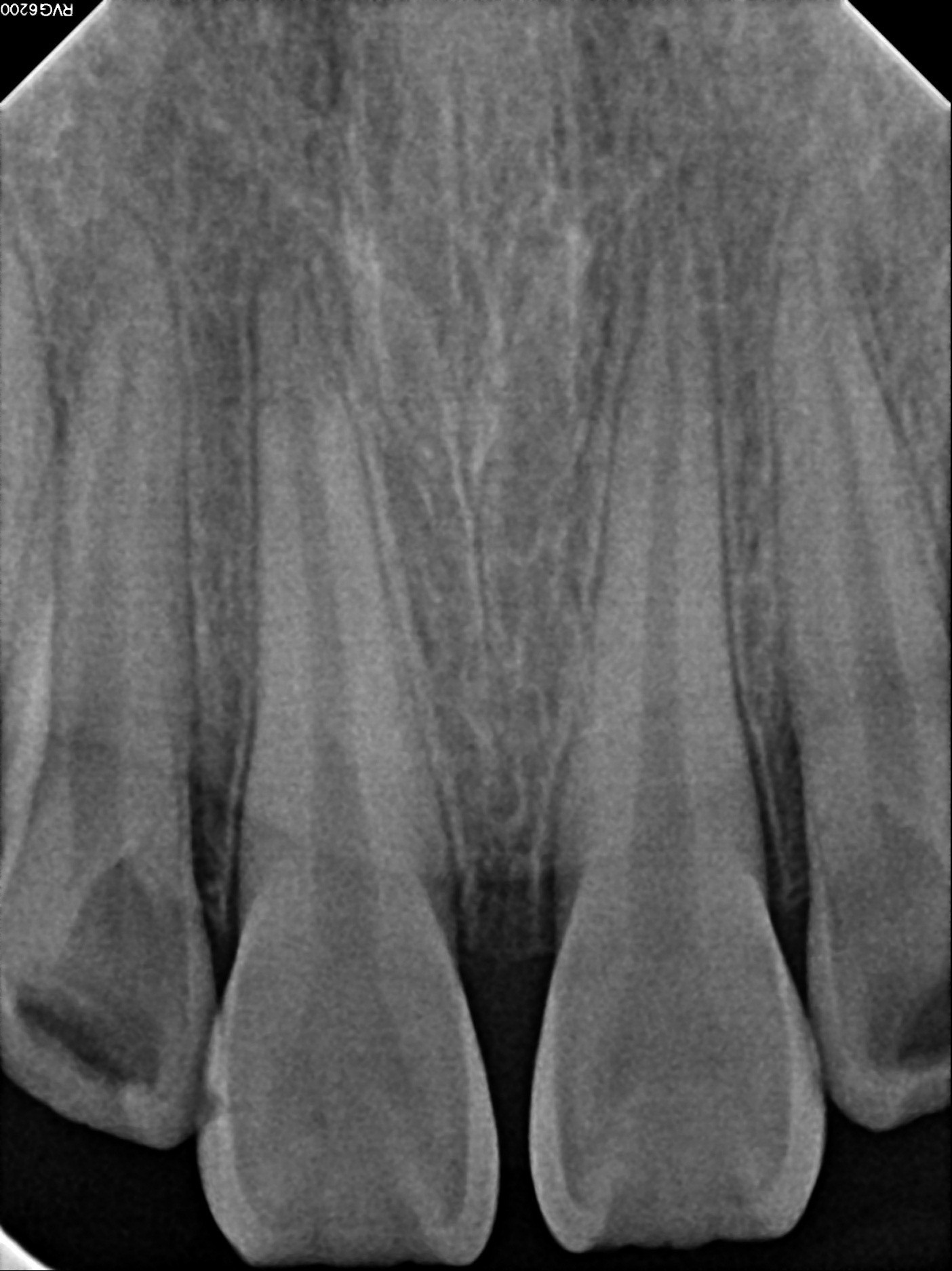

Supplement: Supplementary file 1 — Additional file 1: Test Dataset 1: Digital radiograph of upper posterior teeth. Test Dataset 2: Digital radiograph of upper posterior teeth, Test Dataset 3: Digital radiograph of upper posterior teeth, Test Dataset 4: Digital radiograph of upper posterior teeth, Test Dataset 5: Digital radiograph of upper anterior teeth, Test Dataset 6: Digital radiograph of upper anterior teeth, Test Dataset 7: Digital radiograph of lower posterior teeth, Test Dataset 8: Digital radiograph of upper posterior teeth, Test Dataset 9: Digital radiograph of lower anterior teeth, Test Dataset 10: Digital radiograph of lower anterior teeth, Test Dataset 11: Digital radiograph of lower posterior teeth, Test Dataset 12: Digital radiograph of lower anterior teeth, Test Dataset 13: Digital radiograph of upper posterior teeth, Test Dataset 14: Digital radiograph of lower teeth, Test Dataset 15: Digital radiograph of lower deciduous teeth, Test Dataset 16: Digital radiograph of lower deciduous teeth, Test Dataset 17: Digital radiograph of lower posterior teeth, Test Dataset 18: Digital radiograph of lower deciduous posterior teeth, Test Dataset 19: Digital radiograph of upper posterior teeth, Test Dataset 20: Digital radiograph of lower posterior teeth, Test Dataset 21: Digital radiograph of lower posterior teeth, Test Dataset 22: Digital radiograph of upper posterior teeth, Test Dataset 23: Digital radiograph of upper posterior teeth, Test Dataset 24: Digital radiograph of lower posterior teeth, Test Dataset 25: Digital radiograph of upper posterior teeth, Test Dataset 26: Digital radiograph of lower deciduous posterior teeth, Test Dataset 27: Digital radiograph of lower deciduous posterior teeth, Test Dataset 28: Digital radiograph of lower posterior teeth, Test Dataset 29: Digital radiograph of lower posterior teeth, Test Dataset 30: Digital radiograph of upper deciduous posterior teeth, Test Dataset 31: Digital radiograph of upper anterior teeth, Test Dataset 32: Digital radiograph of lower [file 12903_2023_3251_MOESM1_ESM.zip › Test Dataset 5.jpg]

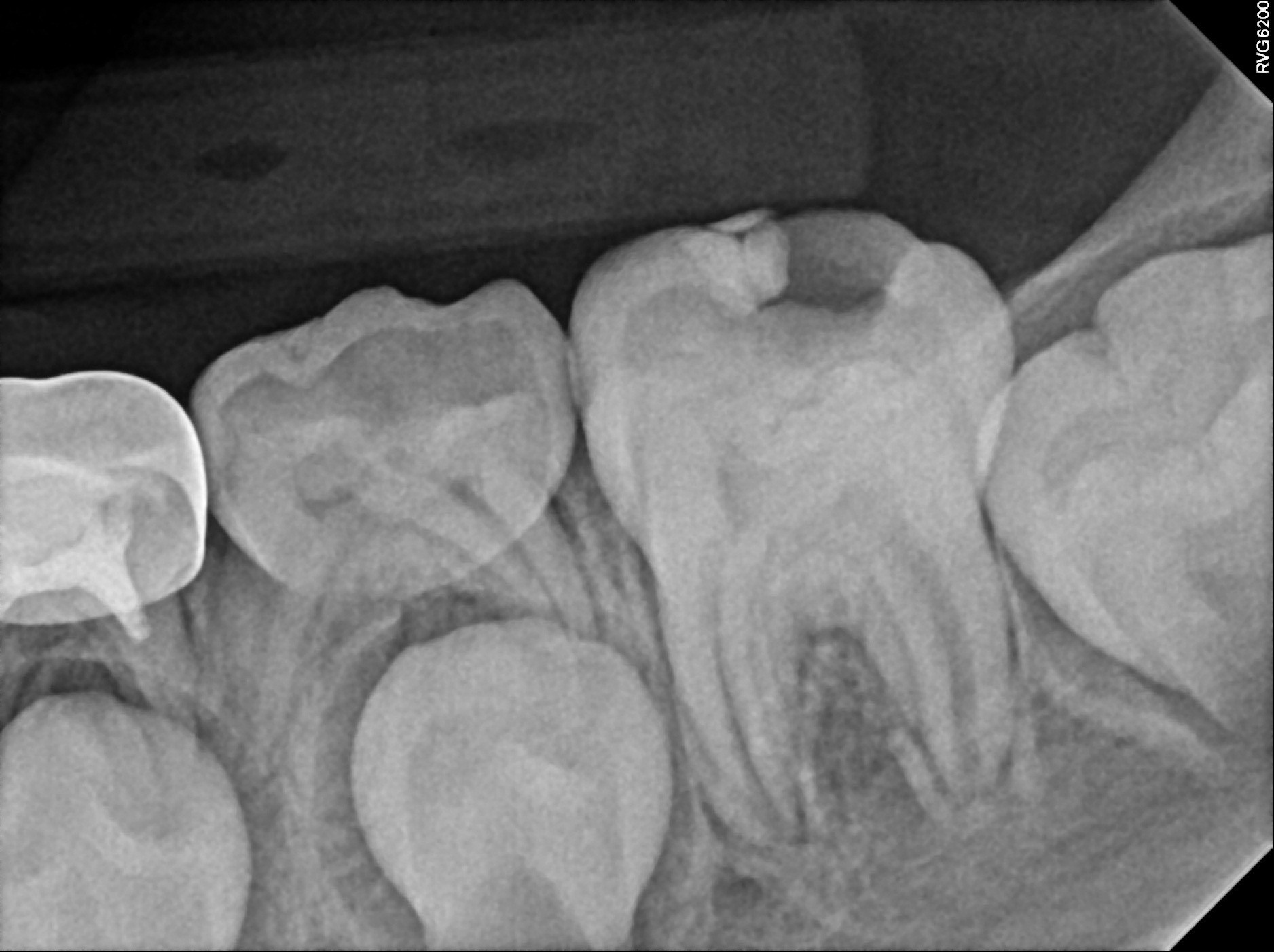

Supplement: Supplementary file 1 — Additional file 1: Test Dataset 1: Digital radiograph of upper posterior teeth. Test Dataset 2: Digital radiograph of upper posterior teeth, Test Dataset 3: Digital radiograph of upper posterior teeth, Test Dataset 4: Digital radiograph of upper posterior teeth, Test Dataset 5: Digital radiograph of upper anterior teeth, Test Dataset 6: Digital radiograph of upper anterior teeth, Test Dataset 7: Digital radiograph of lower posterior teeth, Test Dataset 8: Digital radiograph of upper posterior teeth, Test Dataset 9: Digital radiograph of lower anterior teeth, Test Dataset 10: Digital radiograph of lower anterior teeth, Test Dataset 11: Digital radiograph of lower posterior teeth, Test Dataset 12: Digital radiograph of lower anterior teeth, Test Dataset 13: Digital radiograph of upper posterior teeth, Test Dataset 14: Digital radiograph of lower teeth, Test Dataset 15: Digital radiograph of lower deciduous teeth, Test Dataset 16: Digital radiograph of lower deciduous teeth, Test Dataset 17: Digital radiograph of lower posterior teeth, Test Dataset 18: Digital radiograph of lower deciduous posterior teeth, Test Dataset 19: Digital radiograph of upper posterior teeth, Test Dataset 20: Digital radiograph of lower posterior teeth, Test Dataset 21: Digital radiograph of lower posterior teeth, Test Dataset 22: Digital radiograph of upper posterior teeth, Test Dataset 23: Digital radiograph of upper posterior teeth, Test Dataset 24: Digital radiograph of lower posterior teeth, Test Dataset 25: Digital radiograph of upper posterior teeth, Test Dataset 26: Digital radiograph of lower deciduous posterior teeth, Test Dataset 27: Digital radiograph of lower deciduous posterior teeth, Test Dataset 28: Digital radiograph of lower posterior teeth, Test Dataset 29: Digital radiograph of lower posterior teeth, Test Dataset 30: Digital radiograph of upper deciduous posterior teeth, Test Dataset 31: Digital radiograph of upper anterior teeth, Test Dataset 32: Digital radiograph of lower [file 12903_2023_3251_MOESM1_ESM.zip › Test Dataset 50.jpg]

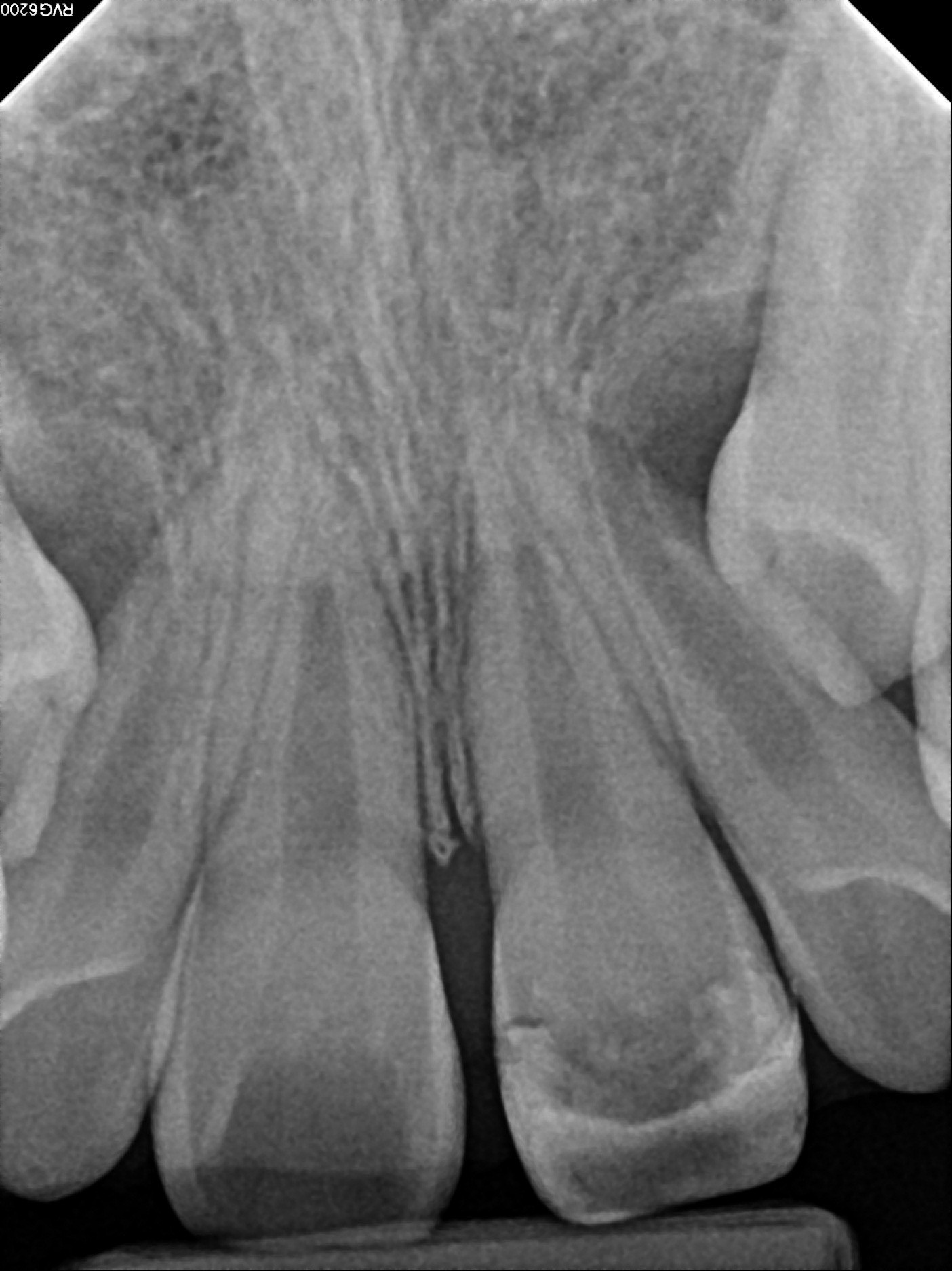

Supplement: Supplementary file 1 — Additional file 1: Test Dataset 1: Digital radiograph of upper posterior teeth. Test Dataset 2: Digital radiograph of upper posterior teeth, Test Dataset 3: Digital radiograph of upper posterior teeth, Test Dataset 4: Digital radiograph of upper posterior teeth, Test Dataset 5: Digital radiograph of upper anterior teeth, Test Dataset 6: Digital radiograph of upper anterior teeth, Test Dataset 7: Digital radiograph of lower posterior teeth, Test Dataset 8: Digital radiograph of upper posterior teeth, Test Dataset 9: Digital radiograph of lower anterior teeth, Test Dataset 10: Digital radiograph of lower anterior teeth, Test Dataset 11: Digital radiograph of lower posterior teeth, Test Dataset 12: Digital radiograph of lower anterior teeth, Test Dataset 13: Digital radiograph of upper posterior teeth, Test Dataset 14: Digital radiograph of lower teeth, Test Dataset 15: Digital radiograph of lower deciduous teeth, Test Dataset 16: Digital radiograph of lower deciduous teeth, Test Dataset 17: Digital radiograph of lower posterior teeth, Test Dataset 18: Digital radiograph of lower deciduous posterior teeth, Test Dataset 19: Digital radiograph of upper posterior teeth, Test Dataset 20: Digital radiograph of lower posterior teeth, Test Dataset 21: Digital radiograph of lower posterior teeth, Test Dataset 22: Digital radiograph of upper posterior teeth, Test Dataset 23: Digital radiograph of upper posterior teeth, Test Dataset 24: Digital radiograph of lower posterior teeth, Test Dataset 25: Digital radiograph of upper posterior teeth, Test Dataset 26: Digital radiograph of lower deciduous posterior teeth, Test Dataset 27: Digital radiograph of lower deciduous posterior teeth, Test Dataset 28: Digital radiograph of lower posterior teeth, Test Dataset 29: Digital radiograph of lower posterior teeth, Test Dataset 30: Digital radiograph of upper deciduous posterior teeth, Test Dataset 31: Digital radiograph of upper anterior teeth, Test Dataset 32: Digital radiograph of lower [file 12903_2023_3251_MOESM1_ESM.zip › Test Dataset 6.jpg]

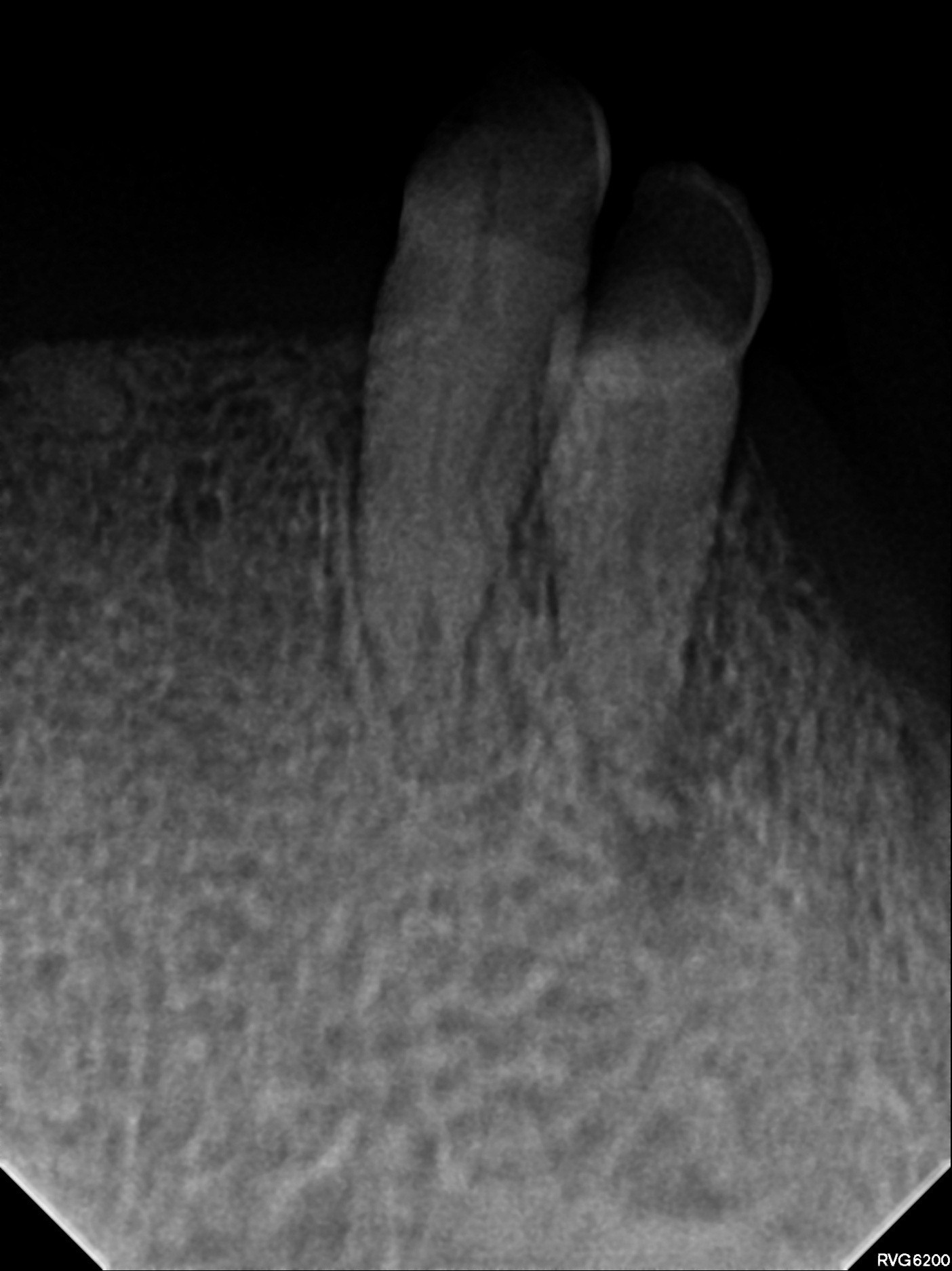

Supplement: Supplementary file 1 — Additional file 1: Test Dataset 1: Digital radiograph of upper posterior teeth. Test Dataset 2: Digital radiograph of upper posterior teeth, Test Dataset 3: Digital radiograph of upper posterior teeth, Test Dataset 4: Digital radiograph of upper posterior teeth, Test Dataset 5: Digital radiograph of upper anterior teeth, Test Dataset 6: Digital radiograph of upper anterior teeth, Test Dataset 7: Digital radiograph of lower posterior teeth, Test Dataset 8: Digital radiograph of upper posterior teeth, Test Dataset 9: Digital radiograph of lower anterior teeth, Test Dataset 10: Digital radiograph of lower anterior teeth, Test Dataset 11: Digital radiograph of lower posterior teeth, Test Dataset 12: Digital radiograph of lower anterior teeth, Test Dataset 13: Digital radiograph of upper posterior teeth, Test Dataset 14: Digital radiograph of lower teeth, Test Dataset 15: Digital radiograph of lower deciduous teeth, Test Dataset 16: Digital radiograph of lower deciduous teeth, Test Dataset 17: Digital radiograph of lower posterior teeth, Test Dataset 18: Digital radiograph of lower deciduous posterior teeth, Test Dataset 19: Digital radiograph of upper posterior teeth, Test Dataset 20: Digital radiograph of lower posterior teeth, Test Dataset 21: Digital radiograph of lower posterior teeth, Test Dataset 22: Digital radiograph of upper posterior teeth, Test Dataset 23: Digital radiograph of upper posterior teeth, Test Dataset 24: Digital radiograph of lower posterior teeth, Test Dataset 25: Digital radiograph of upper posterior teeth, Test Dataset 26: Digital radiograph of lower deciduous posterior teeth, Test Dataset 27: Digital radiograph of lower deciduous posterior teeth, Test Dataset 28: Digital radiograph of lower posterior teeth, Test Dataset 29: Digital radiograph of lower posterior teeth, Test Dataset 30: Digital radiograph of upper deciduous posterior teeth, Test Dataset 31: Digital radiograph of upper anterior teeth, Test Dataset 32: Digital radiograph of lower [file 12903_2023_3251_MOESM1_ESM.zip › Test Dataset 7.jpg]

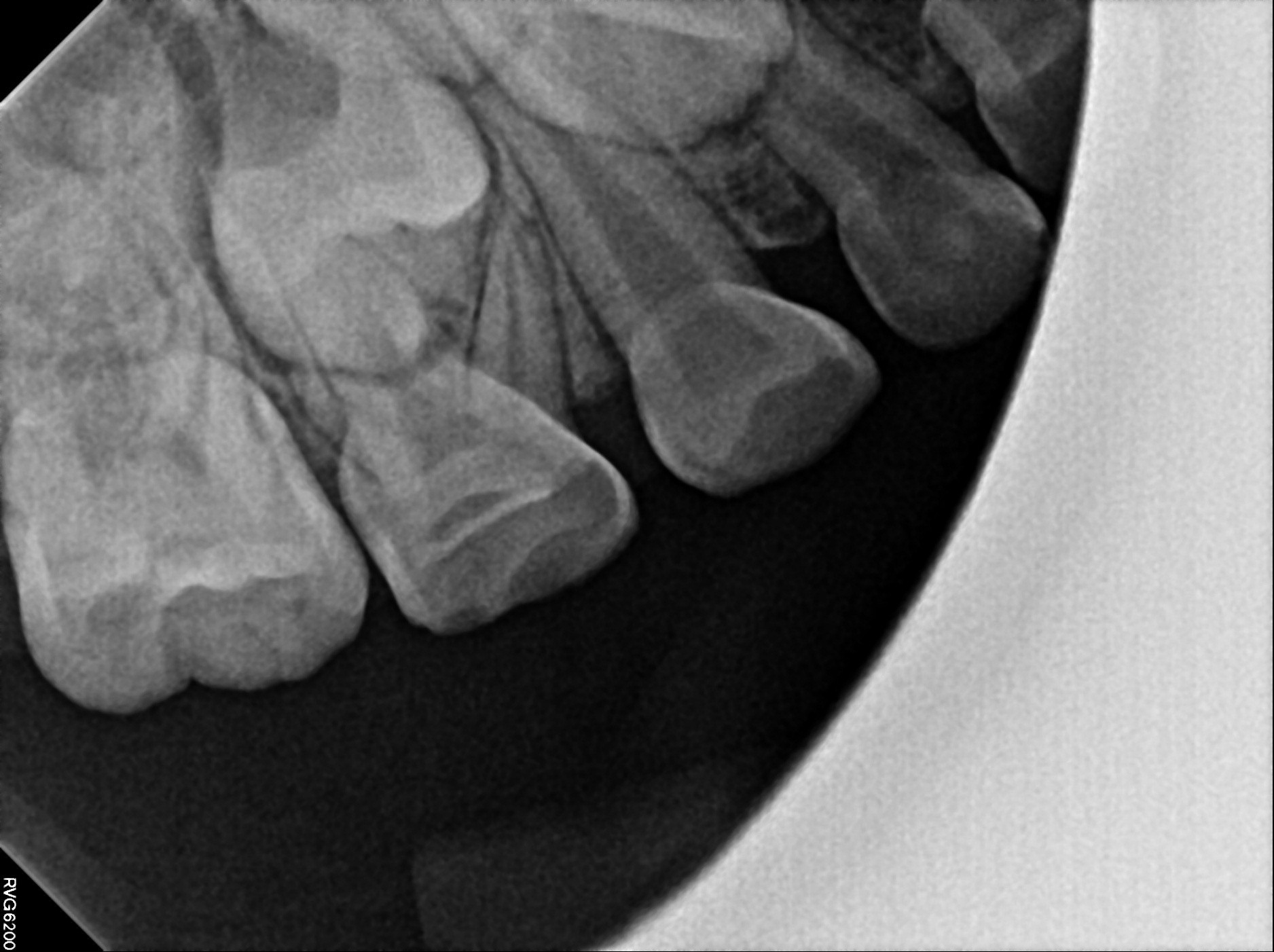

Supplement: Supplementary file 1 — Additional file 1: Test Dataset 1: Digital radiograph of upper posterior teeth. Test Dataset 2: Digital radiograph of upper posterior teeth, Test Dataset 3: Digital radiograph of upper posterior teeth, Test Dataset 4: Digital radiograph of upper posterior teeth, Test Dataset 5: Digital radiograph of upper anterior teeth, Test Dataset 6: Digital radiograph of upper anterior teeth, Test Dataset 7: Digital radiograph of lower posterior teeth, Test Dataset 8: Digital radiograph of upper posterior teeth, Test Dataset 9: Digital radiograph of lower anterior teeth, Test Dataset 10: Digital radiograph of lower anterior teeth, Test Dataset 11: Digital radiograph of lower posterior teeth, Test Dataset 12: Digital radiograph of lower anterior teeth, Test Dataset 13: Digital radiograph of upper posterior teeth, Test Dataset 14: Digital radiograph of lower teeth, Test Dataset 15: Digital radiograph of lower deciduous teeth, Test Dataset 16: Digital radiograph of lower deciduous teeth, Test Dataset 17: Digital radiograph of lower posterior teeth, Test Dataset 18: Digital radiograph of lower deciduous posterior teeth, Test Dataset 19: Digital radiograph of upper posterior teeth, Test Dataset 20: Digital radiograph of lower posterior teeth, Test Dataset 21: Digital radiograph of lower posterior teeth, Test Dataset 22: Digital radiograph of upper posterior teeth, Test Dataset 23: Digital radiograph of upper posterior teeth, Test Dataset 24: Digital radiograph of lower posterior teeth, Test Dataset 25: Digital radiograph of upper posterior teeth, Test Dataset 26: Digital radiograph of lower deciduous posterior teeth, Test Dataset 27: Digital radiograph of lower deciduous posterior teeth, Test Dataset 28: Digital radiograph of lower posterior teeth, Test Dataset 29: Digital radiograph of lower posterior teeth, Test Dataset 30: Digital radiograph of upper deciduous posterior teeth, Test Dataset 31: Digital radiograph of upper anterior teeth, Test Dataset 32: Digital radiograph of lower [file 12903_2023_3251_MOESM1_ESM.zip › Test Dataset 8.jpg]

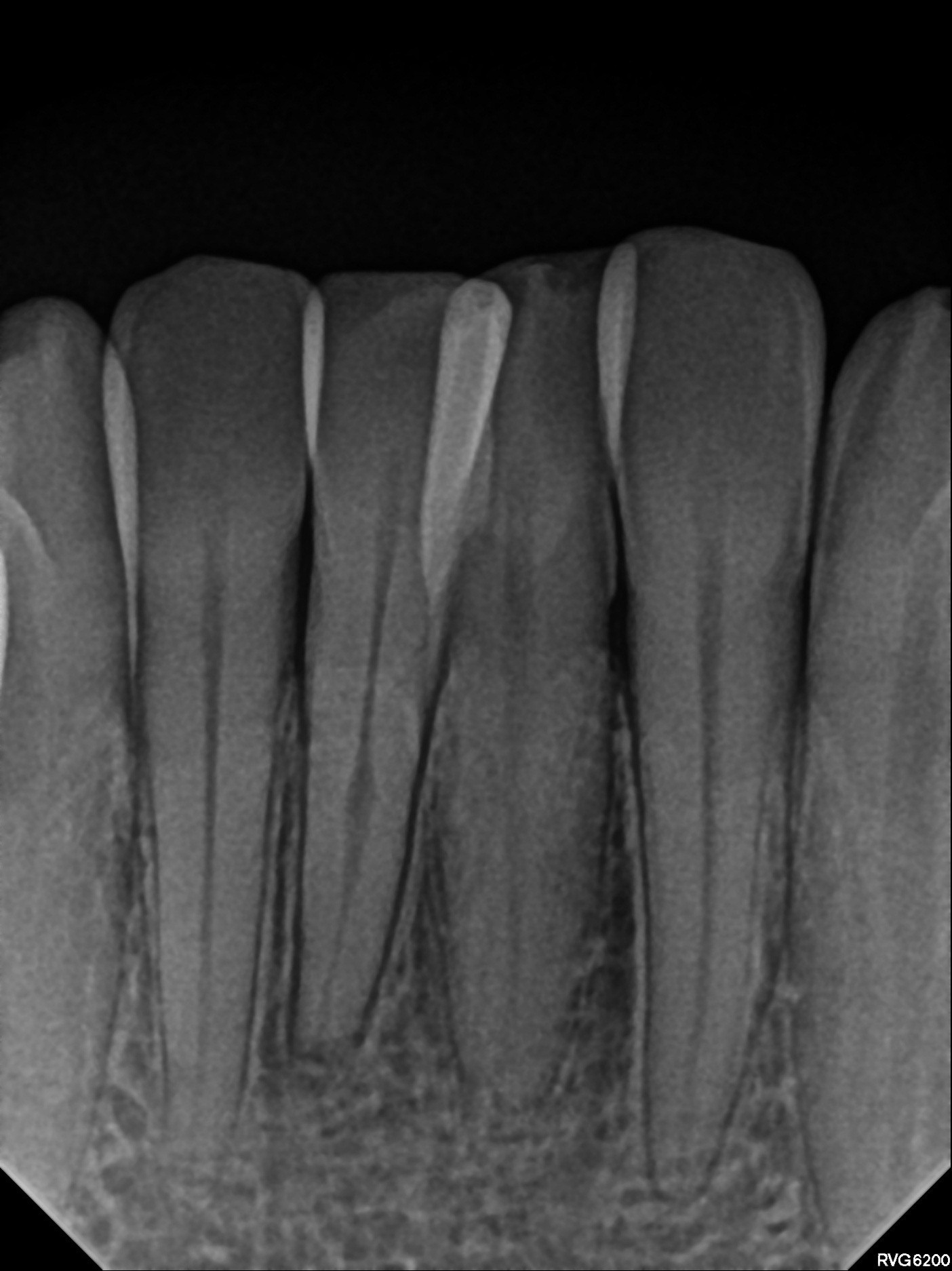

Supplement: Supplementary file 1 — Additional file 1: Test Dataset 1: Digital radiograph of upper posterior teeth. Test Dataset 2: Digital radiograph of upper posterior teeth, Test Dataset 3: Digital radiograph of upper posterior teeth, Test Dataset 4: Digital radiograph of upper posterior teeth, Test Dataset 5: Digital radiograph of upper anterior teeth, Test Dataset 6: Digital radiograph of upper anterior teeth, Test Dataset 7: Digital radiograph of lower posterior teeth, Test Dataset 8: Digital radiograph of upper posterior teeth, Test Dataset 9: Digital radiograph of lower anterior teeth, Test Dataset 10: Digital radiograph of lower anterior teeth, Test Dataset 11: Digital radiograph of lower posterior teeth, Test Dataset 12: Digital radiograph of lower anterior teeth, Test Dataset 13: Digital radiograph of upper posterior teeth, Test Dataset 14: Digital radiograph of lower teeth, Test Dataset 15: Digital radiograph of lower deciduous teeth, Test Dataset 16: Digital radiograph of lower deciduous teeth, Test Dataset 17: Digital radiograph of lower posterior teeth, Test Dataset 18: Digital radiograph of lower deciduous posterior teeth, Test Dataset 19: Digital radiograph of upper posterior teeth, Test Dataset 20: Digital radiograph of lower posterior teeth, Test Dataset 21: Digital radiograph of lower posterior teeth, Test Dataset 22: Digital radiograph of upper posterior teeth, Test Dataset 23: Digital radiograph of upper posterior teeth, Test Dataset 24: Digital radiograph of lower posterior teeth, Test Dataset 25: Digital radiograph of upper posterior teeth, Test Dataset 26: Digital radiograph of lower deciduous posterior teeth, Test Dataset 27: Digital radiograph of lower deciduous posterior teeth, Test Dataset 28: Digital radiograph of lower posterior teeth, Test Dataset 29: Digital radiograph of lower posterior teeth, Test Dataset 30: Digital radiograph of upper deciduous posterior teeth, Test Dataset 31: Digital radiograph of upper anterior teeth, Test Dataset 32: Digital radiograph of lower [file 12903_2023_3251_MOESM1_ESM.zip › Test Dataset 9.jpg]
