# Supplementary material for: Detection of the pathological exposure of pulp using an artificial intelligence tool: a multicentric study over periapical radiographs
Source: BMC Oral Health. 2023 Aug 11;23:553. doi: 10.1186/s12903-023-03251-0 (PMC10416487; doi:10.1186/s12903-023-03251-0)
Supplement: Supplementary file 2 — Additional file 2: STARD 2015 Guidelines. [file 12903_2023_3251_MOESM2_ESM.docx]

| Section  **STARD 2015 Guidelines** | Item No. | STARD 2015 Item | STARD 2015 Outcome | Text Associated |
| --- | --- | --- | --- | --- |
| Title or Abstract | | | | |
|  | 1 | Identification as a study of diagnostic accuracy using at least one measure of accuracy (such as sensitivity, specificity, predictive values or AUC) | - | Detection of the Pathological Exposure of Pulp Using an Artificial Intelligence Tool: A Multicentric Study over Periapical Radiograph The performance metrics used to test MSc performance included mean average precision (mAP), precision, F1 score, recall, and area under receiver operating characteristic curve (AUC). |
| Abstract | | | | |
|  | 2 | Structured summary of study design, methods, results and conclusions (for specific guidance, see STARD for Abstracts) | - | Background: Introducing artificial intelligence (AI) into the medical field proved beneficial in automating tasks and streamlining the practitioners’ lives. Hence, this study was conducted to design and evaluate an AI tool called **Make Sure Caries Detector and Classifier (MSc)** for detecting pathological exposure of pulp on digital periapical radiographs and to compare its performance with dentists. Methods: This study was a diagnostic multi-centric study, with over 3461 digital periapical radiographs from different countries’ and centers. MSc was built using Yolov5-x model, which was used for exposed and unexposed pulp caries detection. The dataset was split into a train, validate, and test dataset; the ratio was 8-1-1 to prevent overfitting. 345 images with 752 labels were randomly allocated to test MSc. The performance metrics used to test MSc performance included mean average precision (mAP), precision, F1 score, recall, and area under receiver operating characteristic curve (AUC). The metrics used to compare the performance with that of 10 certified dentists were: right diagnosis exposed, right diagnosis not exposed, false diagnosis exposed, false diagnosis not exposed, missed diagnosis, and over diagnosis. Results: MSc achieved a performance of more than 90% in all metrics examined: an average precision of 0.928, recall of 0.918, F1-score of 0.922, and an [mAP@ .5](about:blank) (AUC) of 0.956 (P<.05). The results showed a higher mean for all right (correct) diagnosis parameters in MSc group while a higher mean for all wrong diagnosis parameters in the dentists group (P<.05).  Conclusions: The designed MSc tool proved itself reliable in the detection and differentiating between exposed and unexposed pulp. It also showed a better performance when compared to the 10 dentists. |
| Introduction | | | | |
|  | 3 | Scientific and clinical background, including the intended use and clinical role of the index test |  | Lines 67-131 |
|  | 4 | Study objectives and hypotheses |  | Hence, this study was conducted to design and evaluate an AI tool called **Make Sure Caries Detector and Classifier (MSc)** for detecting pathological exposure of pulp on digital periapical radiographs and to compare how correct is the diagnosis between MSc and Dentists. The study was testing the hypothesis to evaluate if the designed AI tool was able to detect exposed/unexposed pulp caries correctly as compared to dentists. |
| Methods | | | | |
| Study Design | 5 | Whether data was collected before or after index test and reference standard were performed |  | This study was designed as a retrospective, diagnostic, and multi-centric study |
| Participants | 6 | Eligibility criteria |  | The research included all sizes of digital radiograph and all carious teeth, including those with periapical or periodontal issues. Additionally, radiographs with noticeable caries by human eyes were included, whether permanent or deciduous, anterior or posterior teeth. Digital periapical radiographs with root caries, restorations (intra-coronal, crowns, and bridges), orthodontic brackets, and wires affecting the interpretation of the carious teeth were excluded. Also, radiographs with more than half of the film missing or unclear, or that are difficult to discern due to extreme distortion, artificial noise, blur, and poor image quality, were omitted. |
|  | 7 | On what basis potentially eligible participants were identified (such as symptoms, results from previous tests, inclusion in registry) |  | Results from other tests |
|  | 8 | Where and when potentially eligible participants were identified (setting, location and dates) |  | were selected between April 2021 and November 2021 from different centers in different countries, including Saudi Arabia *(Specialized Dental Center, Aohd Dental Center, and Alhijra Dental Center), (Faculty of Dentistry, Taibah University)*, Spain *(Faculty of Dentistry, Complutense University of Madrid)*, and Korea *(Faculty of Dentistry Daejeon Dental Hospital)*. The periapical radiographs for this research were retrospectively selected from 18,000 collected periapical radiographs. |
|  | 9 | Whether participants formed a consecutive, random or convenience series |  | By randomly distributing all collected labeled data |
| Test Methods | 10 a | Index test, in sufficient detail to allow replication |  | Lines 256-286 |
|  | 10 b | Reference standard, in sufficient detail to allow replication |  | Line 209-229 |
|  | 11 | Rationale for choosing the reference standard (if alternatives exist) |  | To establish the gold standard |
|  | 12 a | Definition of and rationale for test positivity cut-offs or result categories of the index test, distinguishing pre-specified from exploratory |  | Dichotomous (Exposed or unexposed) |
|  | 12 b | Definition of and rationale for test positivity cut-offs or result categories of the reference standard, distinguishing pre-specified from exploratory |  | Dichotomous (Exposed or unexposed) |
|  | 13 a | Whether clinical information and reference standard results were available to the performers/readers of the index test |  | Data was labeled at different intervals since they were collected at different intervals. While utilizing MSc, the reference standards were available to the performers. |
|  | 13 b | Whether clinical information and index test results were available to the assessors of the reference standard |  | The programmers had full access to all labeled data. |
| Analysis | 14 | Methods for estimating or comparing measures of diagnostic accuracy |  | Lines 304-335 |
|  | 15 | How indeterminate index test or reference standard results were handled |  | The data from each hospital or dental center was cleaned and labeled internally by collaborated, qualified endodontists with more than two years of experience before being sent to the principal investigator (PI) (Specialist of Restorative Dentistry) through an electronic cloud (Google Drive). |
|  | 16 | How missing data on the index test and reference standard were handled |  | In addition, further 16 images were excluded because of inter-rater disagreement |
|  | 17 | Any analyses of variability in diagnostic accuracy, distinguishing pre-specified from exploratory |  | N/A |
|  | 18 | Intended sample size and how it was determined |  | Based on the results of the study, ^(3)^ which aimed to estimate optimal deep CNN algorithm weight factors for training and validation dataset of both carious and non-carious molars and premolars teeth, at diagnostic accuracy 82.0%, sensitivity 81.0%, specificity 83.0%, PPV 82.7%, and NPV 81.4%, and with an alfa error of 5% and a confidence interval of 95%, a sample size of 3000 periapical radiographs in total were chosen. To achieve higher diagnostic performance metrics, the teeth were not classified based on tooth position, and 3445 digital periapical radiographs were selected in this study. |
| Results | | | | |
| Participants | 19 | Flow of participants, using a diagram |  | Line 381 |
|  | 20 | Baseline demographic and clinical characteristics of participants |  | N/A |
|  | 21 a |  |  | The total data assessed for eligibility consisted of 18000 images; however, 14539 images were excluded for not meeting the inclusion criteria and for technical reasons. Most images were excluded because they were caries free; therefore, only 3461 images were randomized. In addition, further 16 images were excluded because of inter-rater disagreement; hence, 3445 images were labeled and annotated (7718 labels in total). The data were split in a ratio of (8:1:1) for train, test, and validate respectively. The train set consisted of 2,755 images (6,171 labels), the validation set consisted of 345 images (795 labels), and the test set comprised of 345 images (752 labels). Lastly, the test dataset was used for analyzing MSc group and dentists group |
|  | 21 b |  |  | The total data assessed for eligibility consisted of 18000 images; however, 14539 images were excluded for not meeting the inclusion criteria and for technical reasons. Most images were excluded because they were caries free; therefore, only 3461 images were randomized. In addition, further 16 images were excluded because of inter-rater disagreement; hence, 3445 images were labeled and annotated (7718 labels in total). The data were split in a ratio of (8:1:1) for train, test, and validate respectively. The train set consisted of 2,755 images (6,171 labels), the validation set consisted of 345 images (795 labels), and the test set comprised of 345 images (752 labels). Lastly, the test dataset was used for analyzing MSc group and dentists group |
|  | 22 | Time interval and any clinical interventions between index test and reference standard |  | N/A |
| Test Results | 23 | Cross tabulation of the index test results (or their distribution) by the results of the reference standard |  | Tabulation at line 403 |
|  | 24 | Estimates of diagnostic accuracy and their precision (such as 95% confidence intervals) |  | The number of true positive (TP) exposed and unexposed pulp caries that were detected by MSc over the test dataset is 691 labels, the number of false positive (FP) exposed and unexposed pulp caries that were detected by MSc over the test dataset is 56 labels, and the number of false negative (FN) exposed and unexposed pulp caries that were detected by MSc over the test dataset is 61 labels. Therefore, the obtained results of our model showed a mAP<0.5 of 95.6%, precision of 92.8%, a recall of 91.8%, and an F1 score of 92.2% (P> .05) (Table 3). The Area Under Curve (AUC) value was 0.956. (p > .05) (Fig. 4). (Fig 5) |
|  | 25 | Any adverse events from performing the index test or the reference standard |  | N/A |
| Discussion | | | | |
|  | 26 | Study limitations, including sources of potential bias, statistical uncertainty, and generalisability |  | There were also more limitations in our study. Clinical parameters were not included, which is an aspect that should be taken into account to have a more accurate diagnosis. Also, Neural Networks, in general, including our tool MSc, are black boxes that cannot explain machine learning characteristics and the grounds for making decisions based on that learning. The limitations of the digital periapical radiographs, such as image magnification and distortion and the lack of three-dimensional information, may lower the MSc tool's diagnostic accuracy. This is because it’s challenging to diagnose critical cases, such as nearly exposed pulp with a small layer of dentine. Also, the dataset wasn’t divergent regarding the age and sex because it was collected without prior knowledge of the patients’ details. |
|  | 27 | Implications for practice, including the intended use and clinical role of the index test |  | The designed AI model proved itself reliable in the detection of pathological pulp exposure, and in the differentiation between exposed and unexposed pulp caries on digital periapical radiographs.  The designed AI model detected pathological pulp exposure on digital periapical radiographs more correctly and effectively than the10 dentists. |
| Other Information | | | | |
|  | 28 | Registration number and name of registry |  | The study protocol was approved by the Institutional Review Board (IRB) in the local committee for ethics of health and scientific research in health affairs in Medina region (IRB 25/2021), |
|  | 29 | Where the full study protocol can be accessed |  | Upon request |
|  | 30 | Sources of funding and other support; role of funders |  | This study was funded by SWC Company. |
