# Supplementary material for: Detection of the pathological exposure of pulp using an artificial intelligence tool: a multicentric study over periapical radiographs
Source: BMC Oral Health. 2023 Aug 11;23:553. doi: 10.1186/s12903-023-03251-0 (PMC10416487; doi:10.1186/s12903-023-03251-0)
Supplement: Supplementary file 3 — Additional file 3: Artificial intelligence in dental research: Checklist for authors, reviewers, readers. [file 12903_2023_3251_MOESM3_ESM.docx]

| Section  **Artificial intelligence in dental research: Checklist for authors, reviewers, readers** | Item No. | AI in Dental Research Item | AI reporting outcome | Text Associated |
| --- | --- | --- | --- | --- |
| Study Goal | 1 | Researchers should early on define the relevance, scope and meaning ofthe AI application they aim to develop or validate. | - | This study was conducted to design and evaluate an AI tool called **Make Sure Caries Detector and Classifier (MSc)** for detecting pathological exposure of pulp on digital periapical radiographs and to compare how correct is the diagnosis between MSc and Dentists. The study was testing the hypothesis to evaluate if the designed AI tool was able to detect exposed/unexposed pulp caries correctly as compared to dentists. |
| Study Focus | 2 | A clear focus on the study aims should be defined, e.g. what goal does the research have (developing a new or validating an existing model, diagnostics or prognostics etc.). | - | This study was established as a diagnostic, multi-centric study with the goal of proposing and evaluating an AI tool that was used for detection of pathological pulp exposure in digital periapical radiographs as well as to compare how correct is the diagnosis between MSc and dentists. |
| Data | 3 | A major aspect when planning AI studies is data; especially in dentistry, datasetsare oftentimes small and imbalanced |  | 3461 anonymized labeled digital periapical radiographs, with 3106 exposed pulp caries and 4612 unexposed pulp caries, were selected between April 2021 and November 2021 from different centers in different countries, including Saudi Arabia *(Specialized Dental Center, Aohd Dental Center, and Alhijra Dental Center), (Faculty of Dentistry, Taibah University)*, Spain *(Faculty of Dentistry, Complutense University of Madrid)*, and Korea *(Faculty of Dentistry Daejeon Dental Hospital)*. |
| Study Aim | 4 | Researchers should have a clear idea if their study is exploratory or hypothesis-testing |  | The study was testing the hypothesis to evaluate if the designed AI tool was able to detect exposed/unexposed pulp caries correctly as compared to dentists. |
| Reference test | 5 | A major difficulty is the construction of the reference test |  | Lines 209-229 |
| Clustering | 6 | When feeding annotated data into the model, it is relevant to consider thespecifics of dental data: Often, multiple datapoints (images, clinical assessment) areavailable from the same patient, either from the same time point |  | Dataset was broken down into three parts: Train, validate, and test, with an (8-1-1) ratio. |
| Test Dataset | 7 |  |  | During data processing, the labeled dataset will be randomly divided into train, validate, and test datasets using Python's random package. |
| Computational resource | 8 | Researchers should, before engaging into AI research,consider the computational resources |  | The MSc model employs multiple CNN algorithm optimization tactics, such as auto learning bounding box anchors, mosaic data augmentation, and the cross-stage partial network. It uses Yolo (You Only Look Once), which isan object detection algorithm. It divided images into cells. Each cell is responsible for detecting objects within it. Yolo uses a single neural network to process the entire picture and then separates it into parts and predicts the bounding boxes for each part. |
| Comparator: | 9 | The model should be compared against relevant alternatives. These couldbe an independent group of dental examiners, possibly of different experience (toreflect the usefulness of the model in different groups) or against other acceptedimagery or clinical tests. For such comparisons, relevant outcomes and outcomemetrics should be used. |  | The metrics used to compare the performance with that of 10 certified dentists were: right diagnosis exposed, right diagnosis not exposed, false diagnosis exposed, false diagnosis not exposed, missed diagnosis, and over diagnosis. The study was testing the hypothesis to evaluate if the designed AI tool was able to detect exposed/unexposed pulp caries correctly as compared to dentists. |
| Title: | 10 | The title should clearly lay out that any kind of AI (shallow or deep machinelearning, or more specifically the type of model like convolutional neural networks orrandom forests, among others) was used. |  | Detection of the Pathological Exposure of Pulp Using an Artificial Intelligence Tool: A Multicentric Study over Periapical Radiographs |
| Abstract: | 11 | The abstract should present a structured summary of the study’s aim,methods, results, and conclusion |  | Lines 41-63 |
| Introduction: | 12 | The introduction should briefly sum up the dental background of the study,if there is one, and deduce the need for an AI solution. It should be made clear if thereis a clinical, a research or a teaching problem. |  | Lines 67-154 |
| Study Design | 13 | It is advisable to provide a short overview about the study design toallow orientation for readers early on. |  | This study was established as a diagnostic, multi-centricstudy with the goal of proposing and evaluating an AI tool that was used for detection of pathological pulp exposure in digital periapical radiographs as well as to compare how correct is the diagnosis between MSc and dentists. Reporting follows the Checklists for STARD 2015^(25)^ and Artificial Intelligence in Dental Research ^(26)^. |
| Data | 14 | As data are the main component of any AI model, this section is particularlyrelevant. |  | The study protocol was approved by the Institutional Review Board (IRB) in the local committee for ethics of health and scientific research in health affairs in Medina region (IRB 25/2021), Daejeon Dental Hospital, Wonkwang University College of Dentistry, and Complutense University of Madrid, Spain. Informed consent was waived by the IRB in the local committee for ethics of health and scientific research in health affairs in Medina region (IRB 25/2021) due to retrospective nature of the study. All methods were performed in accordance with the Declaration of Helsinki.  3461 anonymized labeled digital periapical radiographs, with 3106 exposed pulp caries and 4612 unexposed pulp caries, were selected between April 2021 and November 2021 from different centers in different countries, including Saudi Arabia *(Specialized Dental Center, Aohd Dental Center, and Alhijra Dental Center), (Faculty of Dentistry, Taibah University)*, Spain *(Faculty of Dentistry, Complutense University of Madrid)*, and Korea *(Faculty of Dentistry Daejeon Dental Hospital)*. The periapical radiographs for this research were retrospectively selected from 18,000 collected periapical radiographs. |
| . Reference test: | 15 | A major difficulty in AI studies is the construction of the reference test.The case definition and any kind of grading schemes for sub-types should be defined.The test threshold (positive cutoff), if defined, needs to be explained and justified, as ithas an impact on the model and possibly also comparative dentists’ accuracy. |  | Lines 209-230 |
| Sample size | 16 | For hypothesis-testing studies, the sample size and how it wasdetermined (sample size estimation) needs to be fully explained. Specifically, for dentaldata, researchers should consider clustering effects (lesion being clustered in teeth,teeth in humans, in centers, and all of this often in repeated cross-sections). |  | Based on the results of the study, ^(3)^ which aimed to estimate optimal deep CNN algorithm weight factors for training and validation dataset of both carious and non-carious molars and premolars teeth, at diagnostic accuracy 82.0%, sensitivity 81.0%, specificity 83.0%, PPV 82.7%, and NPV 81.4%, and with an alfa error of 5% and a confidence interval of 95%, a sample size of 3000 periapical radiographs in total were chosen. To achieve higher diagnostic performance metrics, the teeth were not classified based on tooth position, and 3445 digital periapical radiographs were selected in this study. |
| Model | 17 | A complete and detailed description of the model is warranted, allowing toreplicate the employed methods. In particular for neural network models inputs,outputs, intermediate layers, pooling, normalization, regularization, and activationshould be reported. |  | Lines 256-290 |
| Training | 18 | Describe the training procedures in sufficient detail so that another researchercould reproduce the training process. |  | Pre-processing procedures were applied using CLAHE to create more contrasted black-and-white images with clipLimit = 5.0 (Fig 1). The dataset was then divided into three groups: train, validate, and test, using an 8-1-1 ratio. |
|  | 19 | Describe the method and model metric (e.g. accuracy, F1-score) to select the finalmodel and evaluate it against the test set. |  | Primary outcome  Mean Average Precision 0.5 (mAP@0.5): Mean average precisionmAP, calculated by taking the mean AP (accuracy of our AI tool) over all eExposed and uUnexposed pulp caries and/or overall 0.5 (IoU) thresholds  2.6.1.2 Secondary outcome  • Precision (Specificity): The ratio of correctly predicted positive eExposed/uUnexposed pulp caries to the total predicted eExposed/uUnexposed pulp caries: Prec. =TP/ TP + FP  • Recall (Sensitivity): Calculates how many actual eExposed/uUnexposed pulp caries true positives the model has captured, labeling them as positives. Recall = TP/TP + FN  • F1 Score: Defined as the function of precision and recall. It is calculated when a balance between precision and recall is needed. F1 = 2 × Precision × Recall/ Precision + Recall  • AUC: Area under the rReceiver oOperating cCharacteristic (ROC) cCurve (AUC). AUC integrated from (0, 0) to (1, 1) gave the aggregate measure of all possible eExposed and uUnexposed pulp caries detection and classification thresholds. |
| Evaluation | 20 | Researchers should describe the outcome and outcome metric(s) used tomeasure the model’s performance, defining the primary outcome and metric andrelating it to the outlined clinical/teaching/research problem. |  | Lines 304-335 |
|  | 21 | Lay out how uncertainty of the performance metrics values was assessed, how anycomparisons between groups were done and how robust these comparisons were, forexample by subgroup analyses of tooth groups, dentitions, patient risk groups, or datasources (from different centers or machinery). |  | All confirmed radiograph images were sent to 10 clinicians (ages 25-32, endodontists and general practitioners, two females and eight males, and from the organizations from which data was collected)  3461 anonymized labeled digital periapical radiographs with 3106 exposed pulp caries and 4612 unexposed pulp caries were selected between April 2021 and November 2021 from different centers in different countries, including Saudi Arabia *(Specialized Dental Center, Aohd Dental Center, Alhijra Dental Center, andFaculty of Dentistry, Taibah University)*, Spain *(Faculty of Dentistry, Complutense University of Madrid)*, and Korea *(Faculty of Dentistry Daejeon Dental Hospital)*. |
|  | 22 | If feasible, researchers should lay out how the explainability, trustworthiness, andtransparency of the model was assessed. |  | N/a |
| Results | 23 | The flow of data, including those in- and excluded, and data partitions intotraining, validation and test dataset should be clarified; a flowchart may be helpful. |  | Line 380 |
| Discussion | 24 | As mostly recommended, we also see four aspects which should beprovided; a summary, a strengths and limitations sections, a section on findings andtheir implications, and one on future directions. |  | Lines 406-531 |
| Other Information | 25 | Here, recommendations towards authorship and registrationaccording to the International Committee of Medical Journal Editors (ICMJE) should befollowed. |  | Lines 539-567 |
